# Genealogical Diversity of Endogenous Retrovirus in the Jawless Fish Genome

**DOI:** 10.4014/jmb.2306.06028

**Published:** 2023-07-28

**Authors:** Song Jing, Wei Jie, Ma Yongping, Sun Yan, Li Zhi

**Affiliations:** 1 College of Life Sciences, Shaanxi Normal University, Xi'an 710062, P.R. China; 2 College of Chemistry and Biological Engineering, Hechi University, Hechi 546300, P.R. China; 3 College of Environment and Life Sciences, Weinan Normal University, Weinan 714099, P. R. China; 4 College of Biological Sciences and Engineering, North Minzu University, Yinchuan 750021, P.R. China

**Keywords:** Endogenous retrovirus, jawless fish, foamy virus, codon usage, virus infection, co-evolution

## Abstract

Retroviral integration into ancient vertebrate genomes left traces that can shed light on the early history of viruses. In this study, we explored the early evolution of retroviruses by isolating nine Spuma endogenous retroviruses (ERVs) and one Epsilon ERV from the genomes of *Agnatha* and *Chondrichthyes*. Phylogenetic analysis of protein sequences revealed a striking pattern of co-evolution between jawless fish ERV and their host, while shark ERV underwent ancient cross-class viral transmission with jawless fish, ray-finned fish, and amphibians. Nucleotide sequence analysis showed that jawless fish ERV emerged in the Palaeozoic period, relatively later than ray-finned fish ERV. Moreover, codon analysis suggested that the jawless fish ERV employed an infection strategy that mimics the host codon. The genealogical diversity of ERVs in the jawless fish genome highlights the importance of studying different viral species. Overall, our findings provide valuable insights into the evolution of retroviruses and their interactions with their hosts.

## Introduction

The study of endogenous retroviruses (ERVs) is a critical area of research that can enhance our understanding of viral and genomic evolutionary processes. ERV research also has significant biomedical applications, including the development of effective therapeutic and preventive strategies against viruses such as HIV [1]. Retroviral infections result in the integration of the virus into the host germline cell genome, allowing transgenerational transmission of viral genetic elements [2]. However, identifying ancient retroviruses and obtaining adequate samples for analysis remain challenging, adding uncertainty to the origin of retroviruses.

Advancements in whole-animal genome sequencing have enabled the discovery of more ERVs, and offer useful insights into the history of retroviruses, which are estimated to have existed for around 100 million years [3]. Nevertheless, analysis of retroviruses with established evolutionary dynamics, such as foamy viruses, can provide more accurate knowledge on retroviruses, their evolution, and interactions with their hosts.

*Simiispumavirus* is a genus of exogenous viruses belonging to the foamy virus (FV) *Spumaretrovirinae* subfamily of the *Retroviridae* family. Endogenous elements that are similar to exogenous FVs can be found in the host genome and are named ERV-Spuma-host name abbreviation. The presence of FVs in mammalian hosts can be traced back to a century from the emergence of Eutherians [4]. Compared to other retroviruses, FVs have a lower rate of evolution and are conserved in different genera [5]. In addition, foamy viruses rarely undergo cross-species transmission, making their evolutionary history stable and well-documented [6]. FVs are not pathogenic to the host, and infected hosts can reproduce normally, making FV molecular data more available [7]. The unprecedented detail of the knowledge on the evolutionary dynamics of FVs makes this group of viruses one of the most important models for studying the macroevolution of retroviruses.

ERV-Spuma elements have been identified within a variety of vertebrate genomes. Four classes of mammalian endogenous and exogenous FVs have been described, including primate FV (*Pan troglodyte*, SFVcpz), brown greater galago FV (*Otolemur crassicaudatus*, PSFVgal), bovine FV (*Bos taurus*, BFV) and Hoffmann’s two-toed sloth FV (*Choloepus hoffmanni*, SloEFV) [7]. In addition, several FV-like ERVs were identified through sequence similarity in the genomes of amphibians, reptiles, and fish, including the two-lined caecilian endogenous retrovirus (*Rhinatrema bivittatum*, ERV-Spuma-Rbi), Tuatara endogenous retrovirus (*Sphenodon punctatus*, ERV-Spuma-Spu) [8], coelacanth endogenous retrovirus (*Latimeria chalumnae*; CoeEFV) [9], zebrafish endogenous retrovirus (*Danio rerio*; ZFERV) [10], cichlid FV-like ERV (*Amphilophus citrinellus*, AciFLERV) [11], and shark ERV (*Callorhinchus milii*, CmiERV) [12].

Phylogenetic analysis of these viruses showed that CoeEFV and ERV-Spuma-Rbi are located at the base of mammalian FVs, whereas AciFLERV and ZFERV form overlapping monophyletic branches. ERV-Spuma has been found to infect almost all large taxa of vertebrates (Fig. S1). However, despite being considered the ancestors of all vertebrate, no complete structure of ERV-Spuma has been discovered in the genomes of jawless fish (*Agnatha*), which comprises over 1,200 species of vertebrates. The absence of reports identifying ERVs in the genome of these ancient fish presents a significant gap in our understanding of the origin and early history of retroviruses. Therefore, analyzing ERV data from the genome of jawless fish is crucial in revealing vital information on the evolution of retroviruses.

Here, we report ten ERV genomes obtained from jawless and cartilaginous fish belonging to three Spuma sublineages and one Epsilon sublineage. How these retroviruses co-evolved with their vertebrate hosts is also explored. Interestingly, codon usage biases suggest that not all of these ERVs are virus-like. The molecular clock predicts that these retroviral sublineages emerged 281.3 million years ago, which is roughly two to three million years after the first appearance of jawless vertebrates.

## Materials and Methods

### ERV Mining and Consensus Sequence Reconstruction

The genomes of retroviruses typically contain two, long complete inverted repeats called "long terminal repeats" (LTRs), which are interspersed with sequences encoding proteins and non-coding RNAs. Retroviral POL proteins are highly conserved across various species, making it feasible to perform ERV mining by querying the POL protein sequences of known retroviruses. In the first step, the POL protein sequences of representative retroviruses were queried (Table S1). Next, genome-wide similarity searches were conducted on 12 species of *Chondrichthyes* and 5 species of *Agnatha* using the TBLASTN algorithm (Table S2). Based on the TBLASTN results, LTRs were detected upstream and downstream of the hit regions using EDTA software. Following this, phylogenetic analysis of candidate sequences was performed using ClustalW to construct consensus sequences for the temporary ERV genome [12].

In the next step of ERV mining and consensus sequence reconstruction, the same database was searched using POL protein sequences. Sequence fragments with more than 30% similarity and coverage are defined as endogenous retroviral elements (EREs). These EREs were then mapped to the temporary ERVs genome constructed in the first step, resulting in a new recombinant ERV genome sequence. For newly discovered ERVs in the genome, the nomenclature is "ERV-retrovirus genus name n-species name," where "retrovirus genus name" is the species name from the ClustalW phylogenetic analysis. "n" indicates the specific type, where n is 0 for recombinant ERV genomic sequences, and n is 1 or a larger number for different EREs from the same host. "Species name" is indicated by the Latin name abbreviation of the host [13] .This naming convention ensures that ERVs are labeled appropriately and aids in their identification and subsequent analysis.

This genome-wide screening identified 293, 91, 1, 94, 83, and 95 EREs in the small-spotted catshark (*Scyliorhinus canicula*), pacific lamprey (*Entosphenus tridentatus*), offshore stickleback (*Eptatretus burgeri*), arctic lamprey (*Lethenteron camtschaticum*), Far Eastern brook lamprey (*Lethenteron reissneri*), and sea lamprey (*Petromyzon marinus*), respectively (Table S3).

The complete consensus ERV genome sequences (including paired LTRs and three open reading frames, including Pol) of the small-spotted catshark, offshore stickleback, arctic lamprey, Far Eastern brook lamprey and sea lamprey are referred to as ERV-Spuma 0-Sca, ERV-Epsilon.0-Ebu, ERV-Spuma.0-Lca, ERV-Spuma.0-Lre and ERV-Spuma.0-Pma, respectively (Fig. S2). Recombinant genomic sequences of some lineages that failed to combine into a complete genomic structure or differed significantly from the consensus genome clustering were additionally combined and named as a or b lineage genomes, *i.e.*, ERV-Spuma.a-Etr, ERV-Spuma.a-Ebu, ERV-Spuma.a-Lca, ERV-Spuma.a-Lre, and ERV-Spuma.b-Lre. The genomes were annotated using Geneious 11.1.2 (Fig. S2 and Table S4).

To identify the conservativeness of proteins in recombinant ERVs, a Conserved Domain Database (CDD) search was performed, and the distribution of conserved domains was calculated using a Venn diagram (Fig. S3 and Table S5). Upon analysis, all ten consensus ERV genomes contained RT_Rtv(Reverse transcriptases (RTs) from retroviruses), RT_LTR(LTRs in RTs DNA copies), RVT_1(reverse transcriptase gene), and transpos_IS481(IS481 family transposase), while nine genomes contained RT_ZFREV_like(RTs similar to the intact endogenous retrovirus ZFERV from zebrafish). Eight genomes contained Integrase, and five complete recombinant genomes contained RNase H. In addition, ERV-Epsilon.0-Ebu contains the ENVV1-like_HR1-HR2 domain, which is also found in the envelope proteins of human endogenous retroviruses (HERVs). However, when analyzing the conserved domain structure of GAG, it was not found to be present in these genomes. In Ryan’s research [14] , the ERV retrieval results for species such as Rainbow trout and Tongue sole only detected POL protein sequences. In the same article, the gag and env genes of DrFV were found to lack detectable sequence homology with any known RVs, FVs, or other genes, as confirmed by BLAST searches, CD-HIT, and final protein homology screening using HMMER. These could be due to loss of conserved domain traits by ERV sequences during host evolution or insertions that are too old to match existing conserved domain databases.

### Phylogenetic Analysis of Jawless Fish ERVs

To achieve greater accuracy in identifying newly discovered ERV species, a phylogenetic tree was constructed using ten, newly recombinant sequences and other representative Pol amino acid sequences of exogenous and endogenous retroviruses. (Table S6). Phylogenetic analysis using conserved POL proteins is a commonly used algorithm for retroviral genus identification. The sequences were aligned using the default settings of PhyloSuite. MrBayes was used for performing phylogenetic analysis [15].

**Fig. 1:**
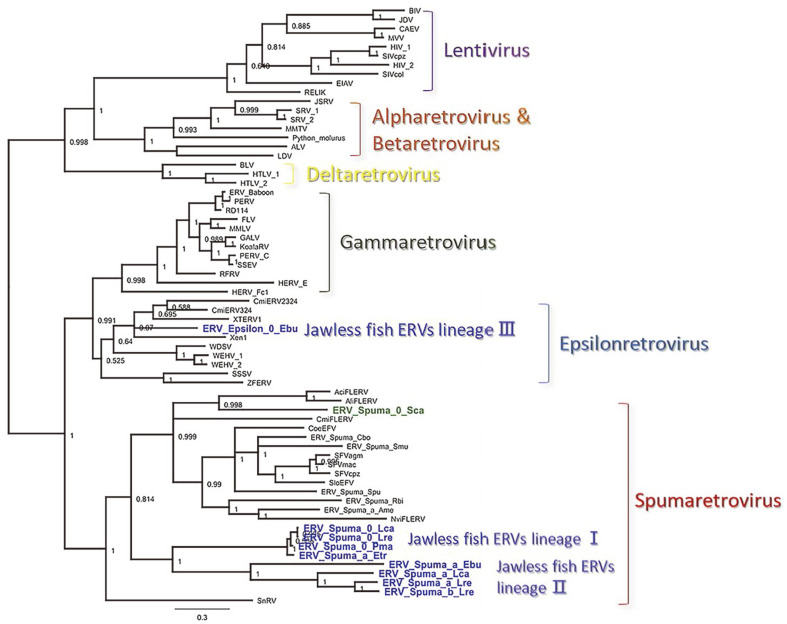
Phylogeny of small-spotted catshark and jawless fish ERVs and other representative retroviruses. The un-rooted phylogeny was reconstructed based on the retrovirus POL protein sequences with PhyloSuite v1.2.2. The numbers on the nodes are Bayesian posterior probability clade support values. Newly assembled ERVs are boldly colored in royal blue and light green. ERVs for jawless fish were divided into three lineages, I, II, and III.

Phylogenetic analysis revealed that the ERV-Spuma.0-Sca from the small-spotted catshark clustered in the FV clade. It grouped with the AciFLERV and AliFLERV FV-like ERVs isolated from ray-finned fish, such as Midas cichlid and killifish (Fig. 1) [11]. The nine newly identified ERVs from jawless fish belonged to three different lineages, with lineages I and II clustering with endogenous FVs. Lineage III ERV from inshore hagfish clustered with Epsilon retroviruses found in the walleye (*Sander vitreus*), amphibians, and sharks [12]. Non-clustering of the three ERV lineages from jawless fish suggests that they do not have a single origin. It is possible that these ERV lineages arose due to four different viral infections. These findings demonstrate that the ERVs in the jawless fish genome exhibit a wide retroviral diversity. The source of these infections could be ray-finned fish, cartilaginous fish, or amphibians inhabiting the same marine environment.

### Estimated Integration Time of ERVs based on LTR Replacement Rate

It is generally assumed that the LTR sequences upstream and downstream are identical during the formation of the invasion genome of ERVs. Furthermore, evolutionary rates of different regions in the genome were set to be the same. Hence, when the substitution rate of the host is known, the base differences between the upstream and downstream LTRs can be utilized to calculate the insertion time of the ERV [12]. The formula for this process is: T = (D/R)/2, where T represents the invasion time in million years (Myr), D denotes the base difference between the 5' and 3' LTRs, and R denotes the neutral evolutionary rate of the host (number of nucleotide substitutions per locus per year). The rate of molecular evolution is typically lower in cartilaginous fishes compared to subgroups of bony vertebrates, including finfishes, coelacanths, and tetrapods. In this study, a rate of 0.5 x 10^-9 substitutions/locus/year was assumed for *S. canicula* based on previous literature [16]. Moreover, the genome of jawless fish generally has a lower rate of base substitution than that of ray-finned fish. Therefore, it was assumed that jawless fish have 1.5 x 10^-9 substitutions/locus/year per locus [17]. To estimate dates, paired LTR sequences longer than 300 nt were used to avoid large computational errors arising from sequences that are too short. These calculations provide crucial insights into the timing of ERV invasions and their interactions with host organisms over time.

The earliest insertion time of the small-spotted catshark ERVs was estimated to be about 35.92 million years (Myr) ago (Table S7). For the jawless fish ERV-Spuma and Epsilon lines, the earliest insertion times were 19.21 and 11.84 Myr ago, respectively. Previous studies approximated that ERVs infected the elephant shark genome about 75 Myr ago, while ERVs invaded the lamprey genome approximately 27–34 Myr ago [12, 18]. Interestingly, a later date was calculated in this study for jawless fish, compared to that for cartilaginous and ray-finned fish. This suggests that the jawless fish ERV may have been transmitted from a more primitive ray-finned fish ERV ancestor. While estimating ERV insertion times based on LTR substitution rates can provide important insights into the evolutionary history of a genome, this approach is subject to limitations, such as mutation rates and selection pressure [19]. Therefore, results obtained through this approach should be interpreted and evaluated cautiously.

### Host-Virus Co-Evolution Analysis

Based on the available fossil record and molecular biology evidence, jawless fishes were among the first vertebrates to appear during the middle Cambrian period, approximately 520 million years ago. Vertebrates, on the other hand, appeared during the late Cambrian period and may have evolved from some jawless fishes. Moreover, there is strong evidence of symbiosis between mammals and their exogenous infectious FVs [14, 20]. Therefore, if the jawless fish ERV co-evolved with jawless fish, it could have significant implications for studying both the origin of retroviruses and the origin of mammals themselves. To gain further insights into the coevolutionary process, newly discovered jawless fish ERVs were combined with known continuous protein sequence data on ray-finned fish, sharks, amphibian, and reptile ERVs (Table S1). Tandem sequences based on GAG, POL, and ENV proteins can provide more information than a single protein. Additionally, host evolution tree information was obtained from the Timetree website, and sequences were aligned using default settings in PhyloSuite. Finally, phylogenetic analysis was performed using MrBayes to better understand these complex interactions and potential evolutionary pathways.

**Fig. 2:**
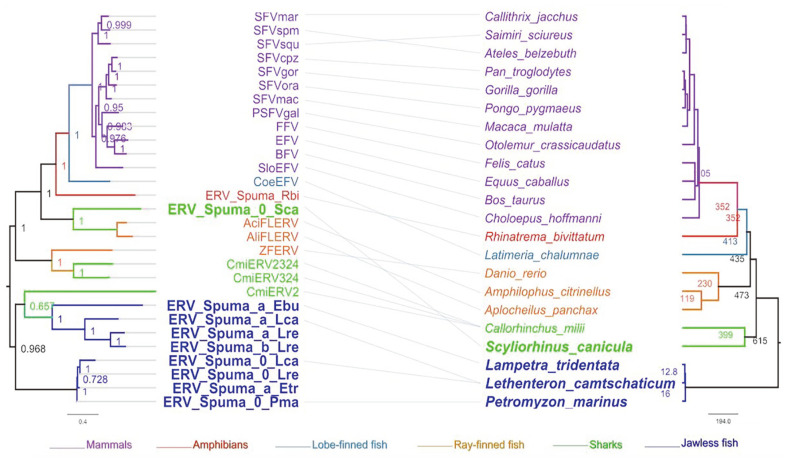
A Comparison of the evolutionary relationships among foamy viruses (left) and their vertebrate hosts (right). The bayesian strategy was employed in the Phylogenetic analysis of all viruses based on protein sequences. At the same time, the phylogenetic relationship of the hosts was retrieved from the Timetree website with previously characterized credible differentiation times. The scale bar indicates the branch lengths of each node on the tree, while for the hosts, it expressly represents the actual speciation time (million years ago, MYA) (right). The new assembled ERVs(left) are bold in Royal blue and light green, respectively. Their host(right) was highlighted using the same colors.

In contrast to the strict one-to-one correspondence of mammalian viruses to their hosts, cross-species transmission was observed in cartilaginous and bony fish ERVs (Fig. 2). Specifically, ERV-Spuma.0-Sca and CmiERVs did not cluster because of their cartilaginous hosts but instead clustered separately with different ray-finned fish ERVs. Furthermore, both jawless fish ERV I and II lineages were located at the root of the left FV tree, in the same position as their host *Agnatha* on the vertebrate tree of life. This reflects a trend of broad co-evolution of jawless fish ERVs with their hosts. Interestingly, the protein sequences of jawless fish ERVs appear to have preserved retroviral features of a more primitive lineage. One possible explanation for this is that they have undergone less evolutionary change compared to their more recently evolved counterparts in other lineages. Alternatively, these features could have been subjected to selective pressures that favored their retention in these lineages.

### Evolutionary Time of ERVs

The rate of virus evolution is influenced by several factors, including the complexity and stability of the genome, mode of transmission, the size and density of the host population, and environmental conditions. In mammals, the symbiotic history of exogenous FVs is very stable, allowing for the evolutionary time of FVs to be inferred from the evolution of mammals [21]. By assuming that cartilaginous and jawless fish follow the same dynamics as mammalian FVs, a molecular clock model can be created to estimate the evolutionary timing of ERVs in small-spotted catsharks and jawless fish based on host phylogenetic results. To construct the host phylogenetic tree, ERV genomic nucleotide data were used, and the molecular clock utilized fossil or inferred time nodes reported in the literature [18]. The ClustalW procedure was used to construct the phylogenetic tree, similar to the method used earlier.

**Fig. 3:**
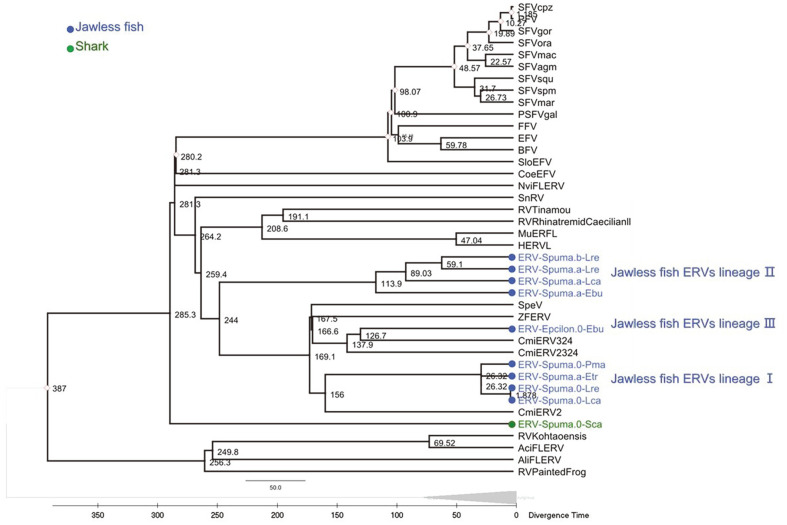
The time scale of FVs and ERVs evolution. A relaxed molecular clock estimated them based on FV and ERV genome nucleotide sequence data. The phylogenetic tree is framed by the Bayesian method. The known time node data marked by red diamonds are cited from the literature. The newly assembled ERVs are colored in blue and green, respectively. Clustal the ERV nucleotide sequences with Geneious 11.1.2 (Table S1). Phylogenetic trees were constructed using Geneious Tree Builder, Genetic Distance Model: Tamura-Nei, Tree Build Method. Build molecular clocks using MEGAX Clocks Compute TimeTree RelTime-ML.

The age of ERV-Spuma.0-Sca was estimated through the molecular clock to be approximately 285.3 Myr (Fig. 3). The shark and ray-finned fish ERVs clustered with the jawless fish ERVs, which separated from other vertebrate ERVs at around 281.3 Myr. Interestingly, the oldest lineages were the ray-finned fish AciFLERV and AliFLERV strains, with an estimated age of about 387 Myr. Additionally, the jawless fish ERV ancestors had a close phylogenetic relationship with ZFERVs and CmiERVs. Although jawless fish species emerged early, the earliest marine vertebrate hosts of retroviruses were more likely to be ray-finned fish. This finding suggests that FVs can readily become endogenous in vertebrates, which is supported by the fact that ERVs are present in the genomes of all major vertebrate species.

### Codon Preference Analysis Identifies Viral Host Specificity and Adaptation Strategies

Codon preference in viruses has been shown to impact viral expression and replication efficiency, as viruses adapt to different host cells, avoid host defense systems, and optimize protein folding [22]. Some viruses may prefer to use codons commonly used by the host cell to maximize the use of the host's transcriptional and translational machinery, while others may select codons with high translation speed and stability to ensure rapid synthesis and correct protein folding [23]. In this study, using the RSCU signature, a new way to identify virus-host specificity was developed. The method calculates the frequency of synonymous codons for each amino acid based on the frequency of codons, and heatmaps were calculated to group major retroviruses and their hosts into two clusters. RSCU values and codon usage frequencies were calculated using DAMBE software. Host CDS data were obtained from http://www.ncbi.nlm.nih.gov/assembly or /nuccore. A list of CDS information for viruses can be found in Table S1. Heatmaps were calculated using https://www.genescloud.cn/chart/HeatMap plotting.

**Fig. 4:**
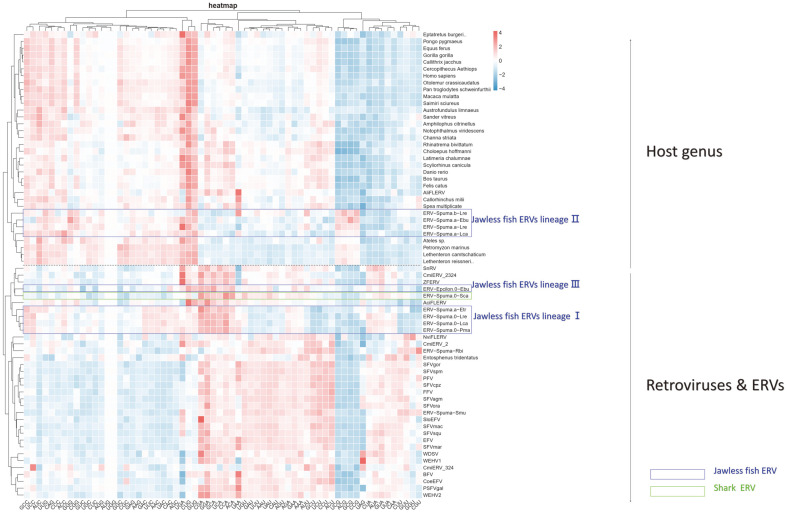
Cluster analysis (Heat map) of Retroviruses or ERVs and their host species' RSCU values. Based on the RSCU values, the heat map shows the values divided into 3 color ranges:< 0 (blue color), 0 (white color), and > 0 (distinctive red). Heatmaps were created using https://www.genescloud.cn/chart/ HeatMap. Default settings were used. RSCU values were displayed in column form by indicating red color for codons with a higher RSCU value, indicating frequent codon usage. The black dashed line indicates the boundary between the host and the virus cluster. There are two colors of frames on the newly assembled ERVs: royal blue and light green.

By analyzing codon preference data, the major retroviruses and their hosts can be grouped into two clusters (Fig. 4). The majority of retroviruses can be found in the cluster under the black dashed line, which is roughly arranged according to their phylogenetic relationships. Although there were some internal differences between the phylogeny-based and codon-based clusters, the vertebrate hosts that retroviruses can infect are all grouped above the black dashed line. This criterion allows for a clear distinction between hosts and virus groups based on their codon usage characteristics. Previous studies did not include ERVs in their analysis [23]. The ERVs in the jawless fish lineages I and III clustered as a separate group below the black line on the viral side. It has been suggested that codon usage features in ERVs contain phylogenetic information. However, it is clear that the nucleotide-based, protein-based and codon-use phylogenetic trees are not identical (Figs. 1-3). In the POL protein-constructed phylogenetic tree, jawless fish ERV lineage III (ERV-Epsilon.0-Ebu) cluster with WDSV, WEHV-1, and WEHV-2 (Epsilon viruses). In contrast, the codon-use phylogenetic tree revealed that jawless fish ERV lineage III (ERV-Epsilon.0-Ebu) and jawless fish ERV lineage I (ERV-Spuma) had similar codon usage.

The upper cluster was observed to have two host subgroups corresponding to Gnathostomata and *Agnatha*. However, it was fascinating to find that the jawless fish lineage II ERVs were clustered with their host in the host subgroup. This suggests that these viral groups could have altered their nucleotide content to utilize the host’s translation system more efficiently. During HIV-1, IAV, and Zika virus infections, several upregulated virus proteins exhibit codon usage patterns that are similar to human genes. This suggests that the virus proteins with host-like codon usage are more efficient in translation [23]. Alternately, viral codon preference could have been modified by host-specific pressures after integration into the genome. This unexpected and intriguing finding may be related to the fact that these viruses are the only strain of the jawless fish ERV family that does not have its complete genome in the host. The high adaptation and transcriptional activity of Pol may have resulted in the loss of Gag and Env open reading frames. Thus, a complete genome with three protein structures and paired LTRs could not be assembled.

It is speculated that the infection strategies of the jawless fish ERV lineage I (ERV-Spuma) and lineage II (ERV-Spuma) may be different. Lineage I have an enhanced survival efficiency by employing a more virus-like codon usage, while lineage II can escape from host defense mechanisms by mimicking host-specific codon usage patterns. Like lineage I , codon usage patterns of HIV-1 major structural proteins, such as GAG, POL, and ENV, deviate significantly from those of the host [24]. The usage of virus-like codons may be associated with fundamental viral functions such as packaging, integration, reverse transcription, and budding, which are essential for viral survival. Even synonymous codons may have significant effects on protein structure and function. Similar to lineage II, HMPV evades innate immunity by disguising itself as a host molecule through m6A modification. Synonymous mutations in m6A methylation of HMPV can lead to increased induction of type I interferon expression [25]. In HIV-1, the early expressed genes (*rev*, *tat*, and *nef*) of HIV-1 are more adapted to the normal human *t*_R_NA pool and have evolved codon usage patterns similar to highly expressed host genes [24]. However, the late genes (*gag*, *pol*, *env*) that primarily elicit immune responses have evolved distinct codon usage patterns.

### Codon Usage Patterns of ERVs and FVs Vary in Specificity Depending on the Host

Previous work on utilizing codon preference to cluster viruses and hosts was expanded upon in this research. Our goal was to determine if codon preference was sufficient to classify virus species according to their hosts. To achieve this, RSCUs were calculated based on the protein-coding sequences of ERVs and FVs (Table S1). PCA principal components were used as summary features of the codon usage patterns of each gene to simplify the data. By plotting principal components 1 (PC1) and 2 (PC2), PCA using RSCU plots could represent the RSCU of ERVs and FVs. The RSCU plots were then analyzed using default settings available at http://www.genescloud.cn/chart/IntePCA.

Fig. S4 demonstrates the relationship between ERVs and FVs with the same host taxa. The figures show that ERVs and FVs of mammals, amphibians and jawless fish can be completely separated based on viral codon usage. This indicates that the codon usage bias of viruses varies in specificity depending on the host. Interestingly, FVs or ERVs of both mammals and amphibians were concentrated in the right quadrant, while ERVs of jawless fish were concentrated in the left quadrant. Moreover, ERVs from cartilaginous fish and ERVs from ray-finned fish spanned multiple regions, suggesting possible cross-species transmission.

The jawless fish ERV lineage I showed similar principal component distributions, with all principal components below the PC2 line. Conversely, lineage II was located above the PC2 line. The respective aggregation of lineage I and II is consistent with the protein and nucleotide phylogenetic tree. However, an exception was found in lineage III, ERV-Epsilon-Ebu, which has a jawless fish host. Despite this, its composition pattern was very similar to that of the ray-finned fish ERV, indicating a close affinity with the latter. This finding is consistent with the results of the phylogenetic analysis of the conserved protein POL and further supports the close relationship between this viral group and the ray-finned fish ERV. Altogether, these findings demonstrate that the codon usage bias of viruses varies in specificity in a host-dependent manner.

## Conclusion

Animal genomics has shed light on retroviruses, which have received increased attention due to their lack of fossil evidence. The classification of retroviruses and retrotransposons needs to be revised based on the observed patterns of ERV distribution and abundance to facilitate studies on retrovirus evolution. In this study, we report the discovery of four new ERV lineages in the shark and jawless fish genomes, revealing their diversity and extending the history of stable co-existence of spumaretroviruses and their hosts beyond Gnathostomata. Although no Gag or Env domains were detected, the classification of shark and jawless fish ERVs as spumaretroviruses and epsilonretroviruses is appropriate due to the similarity in their POL protein sequences with mammalian FVs. The most primitive class of vertebrates infected by retroviruses is the jawless fish, with branching of jawless fish ERVs estimated to have occurred 281.3 Myr ago, according to nucleotide phylogeny. Many ERVs of jawless fish may have been acquired through cross-transmission with ERVs of other ray-finned fish or sharks. Codon preference analysis showed that ERV lineages I and III of jawless fish are closer to FVs, suggesting a strong association between new ERVs and ERV-Spuma. This genetic trait can also be used to differentiate ERVs from different hosts. Species identification and evolutionary relationship analysis of ERVs based on nucleotides, conserved proteins, tandem protein sequences and codon preferences can provide more comprehensive data support for future studies on retroviruses and retrotransposons.

Retroviruses invaded vertebrate genomes over a long evolutionary timescale. As jawless fish ERVs and their hosts evolved together, the integration of jawless fish ERVs should have predated bony fish ERVs. However, based on molecular clock evolution scales, jawless fish ERVs appeared later than ray-finned fish ERVs. This suggests that the ancestors of ray-finned fish ERVs may have infected jawless fish directly or indirectly via cartilaginous fish or amphibians through predation and parasitism. Even modern lampreys still maintain a direct parasitic relationship with sharks, highlighting the potential for cross-species transmission. Since the genome of jawless fish evolved at a slower rate (1.5 × 10^−9^ substitutions per locus per year) than that of some bony fish (1 × 10^−9^ to 2.5 × 10^−9^ substitutions per locus per year), more primitive ERV sequence features are likely preserved in the jawless fish genome. This highlights the importance of further research into ERVs, including their interactions with different host species and their impact on evolution.

## Supplemental Materials

Supplementary data for this paper are available on-line only at http://jmb.or.kr.
