# Supplementary material for: Genealogical Diversity of Endogenous Retrovirus in the Jawless Fish Genome
Source: J Microbiol Biotechnol. 2023 Jul 28;33(11):1412–9. doi: 10.4014/jmb.2306.06028 (PMC10699275; doi:10.4014/jmb.2306.06028)
Supplement: Supplementary file 1 [file jmb-33-11-1412-supple1.pdf]

https://doi.org/10.4014/jmb.2306.06028

Supplementary Figure

Supplementary Figure 1.

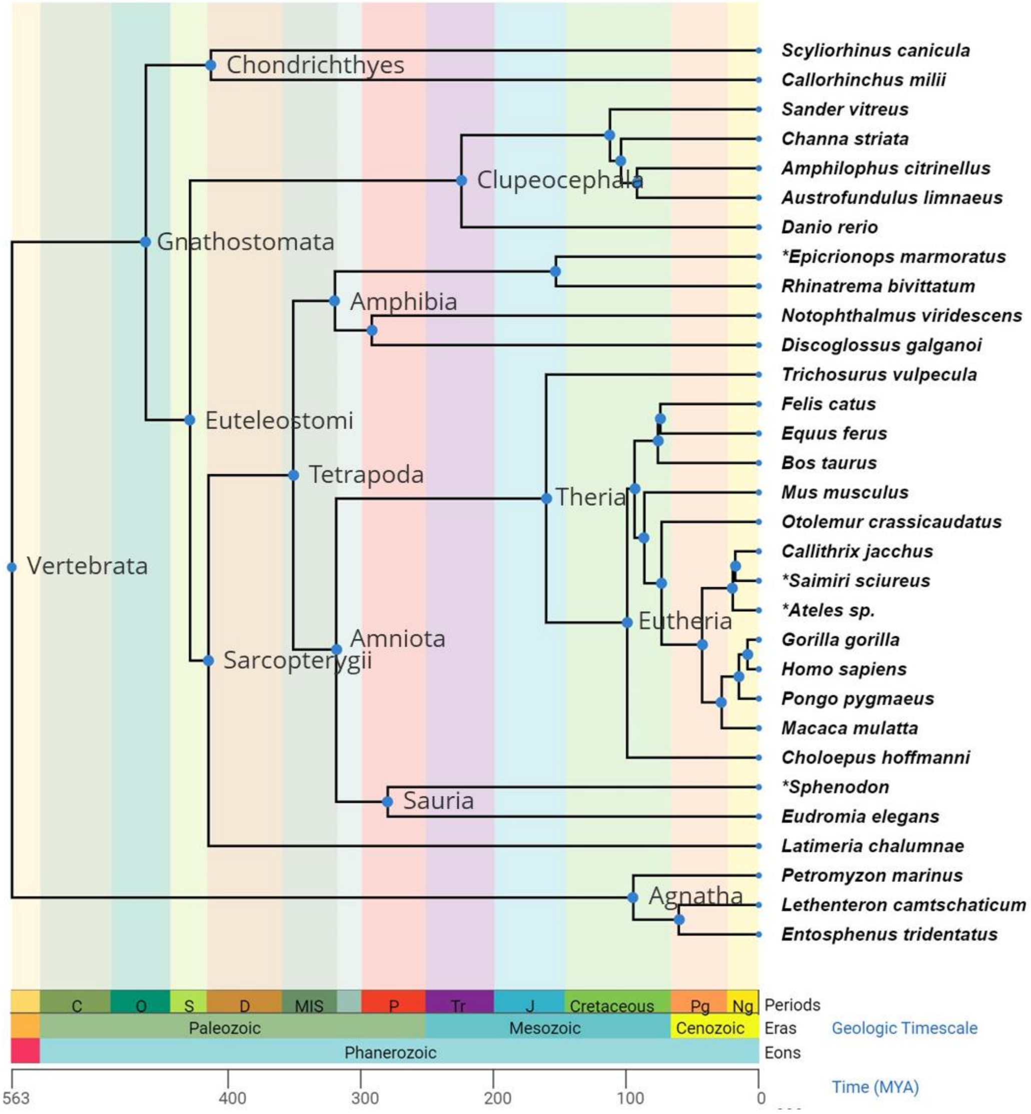

Supplementary Figure 1. Timescale for the vertebrate hosts those retroviruses can infect. The phylogenetic relationship of the hosts was retrieved from the Timetree website with previously characterized credible differentiation times. The timescale is shown in millions of years. Blue dots on nodes show the taxonomic rank. Asterisk (*) indicates substitution of the original species by a closely related species.

Supplementary Figure 2

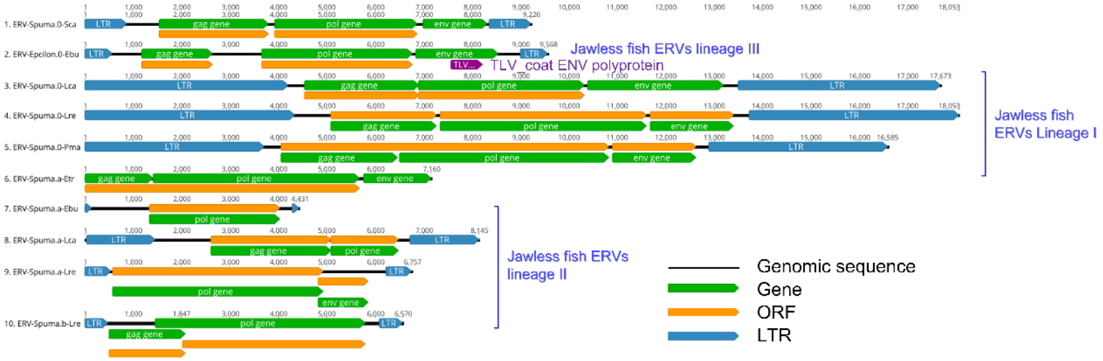

Supplementary Figure 2. Genomic organizations of small spotted catshark and jawless fish ERVs. ORF, open reading frame; LTR, long-terminal repeat. Genomes were annotated using Geneious 11.1.2(Supplementary Table S4). The complete consensus ERV genome sequence (containing paired LTRs and three open reading frames including POL) was named “ERV-retrovirus genus name. 0-species name”, uncomplete genome or lineage different from No.0 genome were named “ERV-retrovirus genus name. a (or b)-species name”. ERVs for jawless fish were divided into three lineages, I, II, and III.

Supplementary Figure 3

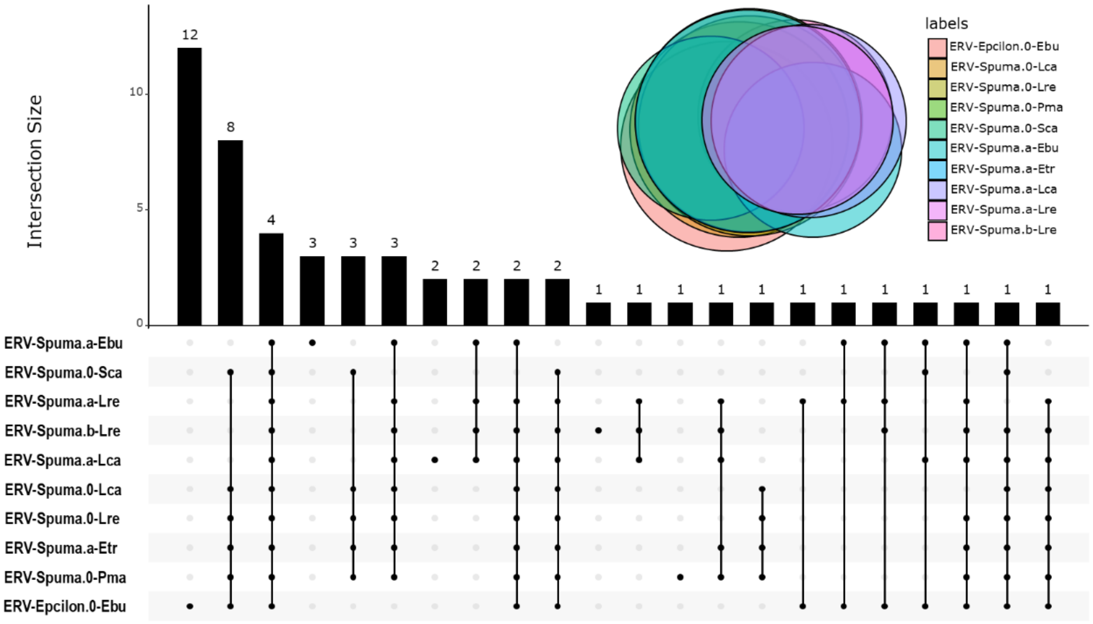

Supplementary Figure 3. Venn diagrams of conserved domains of ERVs in small spotted catshark and jawless fish. The conserved domains of ERV genomes are indicated in Supplementary Table S5a. The intersection dates of ERV genome conserved domains are shown in Supplementary Table S5b.

Supplementary Figure 4

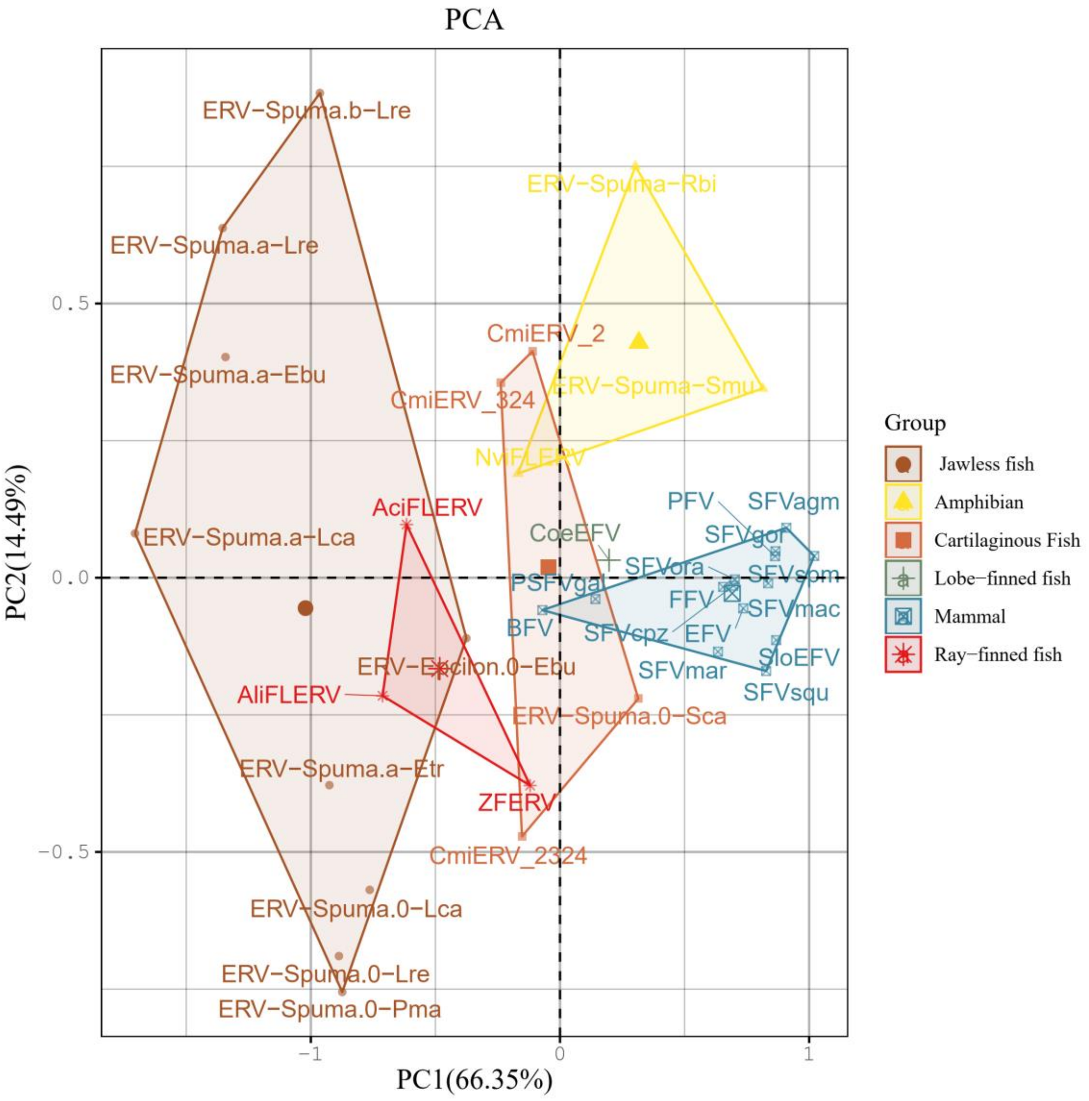

Supplementary Figure 4. Principal component analysis of codon usage frequency of FVs and ERVs. ERVs and FVs are color-coded based on host groups. A PCA of an RSCU graph was used to represent the RSCUs of ERVs and FVs by plotting the principal components 1 (PC1) and 2 (PC2). These ERVs and FVs can be found in Supplementary Table S2. The PCAs of the RSCU graphs were calculated using the following default settings: https://www.genescloud.cn/chart/IntePCA.

Supplementary Table S1. The information of representative retroviruses used for phylogenetic analysis.

No.	Genus	Abbreviation	Natural host	Host genus	Accession No.	Time Tree Replaced with
1	Endogenous retrovirus	ERV-Epcilon.0-Ebu	Jawless fish	*Eptatretus burgeri*	Supplementary Table S4	
2	Endogenous retrovirus	ERV-Spuma.a-Ebu	Jawless fish	*Eptatretus burgeri*	Supplementary Table S4	
3	Endogenous retrovirus	ERV-Spuma.0-Pma	Jawless fish	*Petromyzon marinus*	Supplementary Table S4	
4	Endogenous retrovirus	ERV-Spuma.a-Etr	Jawless fish	*Entosphenus tridentatus*	Supplementary Table S4	*Lampetra tridentata*
5	Endogenous retrovirus	ERV-Spuma.0-Lca	Jawless fish	*Lethenteron camtschaticum*	Supplementary Table S4	
6	Endogenous retrovirus	ERV-Spuma.a-Lca	Jawless fish	*Lethenteron camtschaticum*	Supplementary Table S4	
7	Endogenous retrovirus	ERV-Spuma.0-Lre	Jawless fish	*Lethenteron reissneri*	Supplementary Table S4	
8	Endogenous retrovirus	ERV-Spuma.a-Lre	Jawless fish	*Lethenteron reissneri*	Supplementary Table S4	
9	Endogenous retrovirus	ERV-Spuma.b-Lre	Jawless fish	*Lethenteron reissneri*	Supplementary Table S4	
10	Endogenous retrovirus	ERV-Spuma-Rbi	Amphibians	*Rhinatrema bivittatum*	References 2	
11	Endogenous retrovirus	ERV-Spuma-Smu	Amphibians	*Spea multiplicate*	References 2	
12	Endogenous retrovirus	NviFLERV	Amphibians	*Notophthalmus viridescens*	JF490018	
13	Endogenous retrovirus	ERV-Spuma.0-Sca	Sharks	*Scyliorhinus canicula*	Supplementary Table S4	
14	Endogenous retrovirus	CmiERV2	Sharks	*Callorhinchus milii*	References 1	
15	Endogenous retrovirus	CmiERV324	Sharks	*Callorhinchus milii*	References 1	
16	Endogenous retrovirus	CmiERV2324	Sharks	*Callorhinchus milii*	References 1	
17	Endogenous retrovirus	CoeEFV	Lobe-finned fish	*Latimeria chalumnae*	BAHO01124408	
18	Spumavirus	BFV	Mammals	*Bos taurus*	U94514	
19	Spumavirus	EFV	Mammals	*Equus ferus*	AF201902	
20	Spumavirus	FFV	Mammals	*Felis catus*	Y08851	*Equus caballus*
21	Spumavirus	PFV	Mammals	*Homo sapiens*	Y07725	
22	Spumavirus	PSFVgal	Mammals	*Otolemur crassicaudatus*	KM233624	
23	Spumavirus	SFVagm	Mammals	*Cercopithecus Aethiops*	M74895	
24	Spumavirus	SFVcpz	Mammals	*Pan troglodytes schweinfurthii*	U04327	
25	Spumavirus	SFVgor	Mammals	*Gorilla gorilla*	HM245790	*Pan troglodytes*
26	Spumavirus	SFVmac	Mammals	*Macaca mulatta*	X54482	
27	Spumavirus	SFVmar	Mammals	*Callithrix jacchus*	GU356395	
28	Spumavirus	SFVora	Mammals	*Pongo pygmaeus*	AJ544579	
29	Spumavirus	SFVspm	Mammals	*Ateles sp.*	EU010385	*Ateles belzebuth*
30	Spumavirus	SFVsqu	Mammals	*Saimiri sciureus*	GU356394	*Saimiri boliviensis*
31	Spumavirus	SloEFV	Mammals	*Choloepus hoffmanni*	ABVD02350954	
32	Endogenous retrovirus	AciFLERV	Ray-finned fish	*Amphilophus citrinellus*	CCOE01002087	
33	Endogenous retrovirus	AliFLERV	Ray-finned fish	*Austrofundulus limnaeus*	LDAR01108616	
34	unclassified Orthoretrovirinae	SnRV	Ray-finned fish	*Channa striata*	U26458	*Aplocheilus panchax*
35	Endogenous retrovirus	ZFERV	Ray-finned fish	*Danio rerio*	CABZ01054182	
36	Epsilonretrovirus	WDSV	Ray-finned fish	*Sander vitreus*	AF033822.1	
37	Epsilonretrovirus	WEHV1	Ray-finned fish	*Sander vitreus*	AF133051.1	
38	Epsilonretrovirus	WEHV2	Ray-finned fish	*Sander vitreus*	AF133052.1	
39	Endogenous retrovirus	HERVL	Mammals	*Homo sapiens*	X89211	
40	Retroviridae	RVCommonpossum	Mammals	*Trichosurus vulpecula*	AJ225211	
41	Retroviridae	₹VRhinatremidCaecilianI	Amphibians	*Epicrionops marmoratus*	AJ225225	*Epicrionops*
42	Retroviridae	RVTinamou	Birds	*Eudromia elegans*	AJ225235	
43	Gammaretrovirus	RVKohtaoensis	Amphibians	*RVKohtaoensis*	AJ236118	
44	Retroviridae	SpeV	Reptiles	*Sphenodon*	X85037	*Sphenodon punctatus*
45	Retroviridae	RVPaintedFrog	Amphibians	*Discoglossus galganoi*	AJ225219	
46	Endogenous retrovirus	MuERFL	Mammals	*Mus musculus*	Y12713	

References 1 Han, G.-Z. (2015). "Extensive retroviral diversity in shark." Retrovirology 12(1): 34.

References 2 Wei, X., et al. (2019). "A reptilian endogenous foamy virus sheds light on the early evolution of retroviruses." Virus Evol 5(1): vez001.

lementary Table S2. The information of 12 Chondrichthyes and 5 Agnatha used for data mining

**Species Name**	**Class**	**Taxid**
Amblyraja radiata	Chondrichthyes	386614
Callorhinchus milii	Chondrichthyes	7868
Carcharodon carcharias	Chondrichthyes	13397
Chiloscyllium plagiosum	Chondrichthyes	36176
Chiloscyllium punctatum	Chondrichthyes	137246
Hemiscyllium ocellatum	Chondrichthyes	170820
Hydrolagus affinis	Chondrichthyes	1459831
Leucoraja erinacea	Chondrichthyes	7782
Pristis pectinata	Chondrichthyes	685728
Rhincodon typus	Chondrichthyes	259920
Scyliorhinus canicula	Chondrichthyes	7830
Scyliorhinus torazame	Chondrichthyes	75743
Entosphenus tridentatus	Agnatha	245074
Eptatretus burgeri	Agnatha	7764
Lethenteron camtschaticum	Agnatha	980415
Lethenteron reissneri	Agnatha	7753
Petromyzon marinus	Agnatha	7757

Supplementary Table S3. The EREs sequences used for genome construction.

ERV Name	Contig Number	Contig Number	Location	Contig Size	Sequence Length
ERV-Spuma.1-Sca	CACTIS020000039.1:109059-110834	CACTIS020000039.1	109059-110834	443910	1776
ERV-Spuma.2-Sca	CACTIS020000039.1:235536-238457	CACTIS020000039.1	235536-238457	443910	2922
ERV-Spuma.3-Sca	CACTIS020000039.1:c439822-438698	CACTIS020000039.1	c439822-438698	443910	1125
ERV-Spuma.4-Sca	CACTIS020000164.1:c46793-43872	CACTIS020000164.1	c46793-43872	50774	2922
ERV-Spuma.5-Sca	CACTIS020000343.1:c52900-50858	CACTIS020000343.1	c52900-50858	51110	2043
ERV-Spuma.6-Sca	CACTIS020000748.1:721450-724371	CACTIS020000748.1	721450-724371	793529	2922
ERV-Spuma.7-Sca	CACTIS020000748.1:c99800-98745	CACTIS020000748.1	c99800-98745	793529	1056
ERV-Spuma.8-Sca	CACTIS020000774.1:c29759-28452	CACTIS020000774.1	c29759-28452	28449	1308
ERV-Spuma.9-Sca	CACTIS020000774.1:c31122-29899	CACTIS020000774.1	c31122-29899	28449	1224
ERV-Spuma.10-Sca	CACTIS020001267.1:99799-102720	CACTIS020001267.1	99799-102720	128504	2922
ERV-Spuma.11-Sca	CACTIS020001316.1:261356-264277	CACTIS020001316.1	261356-264277	249764	2922
ERV-Spuma.12-Sca	CACTIS020001345.1:c67922-65001	CACTIS020001345.1	c67922-65001	362374	2922
ERV-Spuma.13-Sca	CACTIS020001550.1:36679-39600	CACTIS020001550.1	36679-39600	167639	2922
ERV-Spuma.14-Sca	CACTIS020001847.1:c400993-398255	CACTIS020001847.1	c400993-398255	405613	2739
ERV-Spuma.15-Sca	CACTIS020001964.1:c77792-76638	CACTIS020001964.1	c77792-76638	265933	1155
ERV-Spuma.16-Sca	CACTIS020002249.1:c156250-155027	CACTIS020002249.1	c156250-155027	326178	1224
ERV-Spuma.17-Sca	CACTIS020002249.1:c157971-156190	CACTIS020002249.1	c157971-156190	326178	1782
ERV-Spuma.18-Sca	CACTIS020002475.1:58736-60283	CACTIS020002475.1	58736-60283	106202	1548
ERV-Spuma.19-Sca	CACTIS020002475.1:60298-61473	CACTIS020002475.1	60298-61473	106202	1176
ERV-Spuma.20-Sca	CACTIS020002475.1:c104205-103234	CACTIS020002475.1	c104205-103234	106202	972
ERV-Spuma.21-Sca	CACTIS020002551.1:c151124-149685	CACTIS020002551.1	c151124-149685	239338	1440
ERV-Spuma.22-Sca	CACTIS020002662.1:c44649-41728	CACTIS020002662.1	c44649-41728	43408	2922
ERV-Spuma.23-Sca	CACTIS020002664.1:125180-126655	CACTIS020002664.1	125180-126655	128916	1476
ERV-Spuma.24-Sca	CACTIS020002754.1:c39477-36556	CACTIS020002754.1	c39477-36556	492103	2922
ERV-Spuma.25-Sca	CACTIS020002783.1:42089-44482	CACTIS020002783.1	42089-44482	226167	2394
ERV-Spuma.26-Sca	CACTIS020002927.1:437985-439445	CACTIS020002927.1	437985-439445	443375	1461
ERV-Spuma.27-Sca	CACTIS020002927.1:439463-440890	CACTIS020002927.1	439463-440890	443375	1428
ERV-Spuma.28-Sca	CACTIS020003204.1:c68544-66664	CACTIS020003204.1	c68544-66664	568782	1881
ERV-Spuma.29-Sca	CACTIS020003317.1:302952-305873	CACTIS020003317.1	302952-305873	308100	2922
ERV-Spuma.30-Sca	CACTIS020003369.1:c219260-218037	CACTIS020003369.1	c219260-218037	1165178	1224
ERV-Spuma.31-Sca	CACTIS020003369.1:c220968-219229	CACTIS020003369.1	c220968-219229	1165178	1740
ERV-Spuma.32-Sca	CACTIS020003419.1:c221696-218775	CACTIS020003419.1	c221696-218775	219006	2922
ERV-Spuma.33-Sca	CACTIS020003667.1:438174-441095	CACTIS020003667.1	438174-441095	443519	2922
ERV-Spuma.34-Sca	CACTIS020003673.1:c417931-416084	CACTIS020003673.1	c417931-416084	422653	1848
ERV-Spuma.35-Sca	CACTIS020003673.1:c418994-417948	CACTIS020003673.1	c418994-417948	422653	1047
ERV-Spuma.36-Sca	CACTIS020003691.1:220546-222435	CACTIS020003691.1	220546-222435	1150920	1890
ERV-Spuma.37-Sca	CACTIS020003691.1:680644-682521	CACTIS020003691.1	680644-682521	1150920	1878
ERV-Spuma.38-Sca	CACTIS020003691.1:1147515-1148510	CACTIS020003691.1	1147515-1148510	1150920	996
ERV-Spuma.39-Sca	CACTIS020003691.1:c396756-395629	CACTIS020003691.1	c396756-395629	1150920	1128
ERV-Spuma.40-Sca	CACTIS020003691.1:c596234-593313	CACTIS020003691.1	c596234-593313	1150920	2922
ERV-Spuma.41-Sca	CACTIS020003802.1:372728-375355	CACTIS020003802.1	372728-375355	377581	2628
ERV-Spuma.42-Sca	CACTIS020003990.1:c210968-210006	CACTIS020003990.1	c210968-210006	722114	963
ERV-Spuma.43-Sca	CACTIS020003990.1:c718440-715702	CACTIS020003990.1	c718440-715702	722114	2739
ERV-Spuma.44-Sca	CACTIS020004246.1:c180087-178876	CACTIS020004246.1	c180087-178876	600888	1212
ERV-Spuma.45-Sca	CACTIS020004246.1:c383889-382000	CACTIS020004246.1	c383889-382000	600888	1890
ERV-Spuma.46-Sca	CACTIS020004246.1:c410607-409606	CACTIS020004246.1	c410607-409606	600888	1002
ERV-Spuma.47-Sca	CACTIS020004330.1:267403-270324	CACTIS020004330.1	267403-270324	272644	2922
ERV-Spuma.48-Sca	CACTIS020004375.1:196896-199817	CACTIS020004375.1	196896-199817	202235	2922
ERV-Spuma.49-Sca	CACTIS020004375.1:c123907-122774	CACTIS020004375.1	c123907-122774	202235	1134
ERV-Spuma.50-Sca	CACTIS020004375.1:c125373-124198	CACTIS020004375.1	c125373-124198	202235	1176
ERV-Spuma.51-Sca	CACTIS020004662.1:c33079-32057	CACTIS020004662.1	c33079-32057	312050	1023
ERV-Spuma.52-Sca	CACTIS020004662.1:c57784-55046	CACTIS020004662.1	c57784-55046	312050	2739
ERV-Spuma.53-Sca	CACTIS020004662.1:c308519-307230	CACTIS020004662.1	c308519-307230	312050	1290
ERV-Spuma.54-Sca	CACTIS020004865.1:156891-158453	CACTIS020004865.1	156891-158453	160668	1563
ERV-Spuma.55-Sca	CACTIS020005044.1:90590-92191	CACTIS020005044.1	90590-92191	419901	1602
ERV-Spuma.56-Sca	CACTIS020005044.1:92585-93688	CACTIS020005044.1	92585-93688	419901	1104
ERV-Spuma.57-Sca	CACTIS020005044.1:c284578-283364	CACTIS020005044.1	c284578-283364	419901	1215
ERV-Spuma.58-Sca	CACTIS020005044.1:c416360-414471	CACTIS020005044.1	c416360-414471	419901	1890
ERV-Spuma.59-Sca	CACTIS020005346.1:c169499-168324	CACTIS020005346.1	c169499-168324	173236	1176
ERV-Spuma.60-Sca	CACTIS020005559.1:c263033-260112	CACTIS020005559.1	c263033-260112	277475	2922
ERV-Spuma.61-Sca	CACTIS020005744.1:41404-44346	CACTIS020005744.1	41404-44346	556680	2943
ERV-Spuma.62-Sca	CACTIS020005886.1:183383-186304	CACTIS020005886.1	183383-186304	188847	2922
ERV-Spuma.63-Sca	CACTIS020006041.1:35304-38225	CACTIS020006041.1	35304-38225	44331	2922
ERV-Spuma.64-Sca	CACTIS020006078.1:178642-181563	CACTIS020006078.1	178642-181563	335577	2922
ERV-Spuma.65-Sca	CACTIS020006278.1:c6052-3131	CACTIS020006278.1	c6052-3131	76851	2922
ERV-Spuma.66-Sca	CACTIS020007245.1:183335-184330	CACTIS020007245.1	183335-184330	383848	996
ERV-Spuma.67-Sca	CACTIS020007245.1:c232452-231361	CACTIS020007245.1	c232452-231361	383848	1092
ERV-Spuma.68-Sca	CACTIS020007245.1:c379425-376477	CACTIS020007245.1	c379425-376477	383848	2949
ERV-Spuma.69-Sca	CACTIS020007245.1:c408500-407118	CACTIS020007245.1	c408500-407118	383848	1383
ERV-Spuma.70-Sca	CACTIS020007491.1:c138398-135735	CACTIS020007491.1	c138398-135735	136860	2664
ERV-Spuma.71-Sca	CACTIS020007841.1:209600-210670	CACTIS020007841.1	209600-210670	208762	1071
ERV-Spuma.72-Sca	CACTIS020007841.1:545065-546954	CACTIS020007841.1	545065-546954	208762	1890

ERV-Spuma.73-Sca	CACTIS020008215.1:c28636-26612	CACTIS020008215.1	c28636-26612	422598	2025
ERV-Spuma.74-Sca	CACTIS020008384.1:178856-179833	CACTIS020008384.1	178856-179833	280792	978
ERV-Spuma.75-Sca	CACTIS020008384.1:c277015-274094	CACTIS020008384.1	c277015-274094	280792	2922
ERV-Spuma.76-Sca	CACTIS020008911.1:119813-121561	CACTIS020008911.1	119813-121561	73315	1749
ERV-Spuma.77-Sca	CACTIS020008911.1:c50139-47218	CACTIS020008911.1	c50139-47218	73315	2922
ERV-Spuma.78-Sca	CACTIS020008914.1:70262-71485	CACTIS020008914.1	70262-71485	114360	1224
ERV-Spuma.79-Sca	CACTIS020008914.1:71620-73182	CACTIS020008914.1	71620-73182	114360	1563
ERV-Spuma.80-Sca	CACTIS020008914.1:111400-112371	CACTIS020008914.1	111400-112371	114360	972
ERV-Spuma.81-Sca	CACTIS020008914.1:c8634-7585	CACTIS020008914.1	c8634-7585	114360	1050
ERV-Spuma.82-Sca	CACTIS020008914.1:c63925-62636	CACTIS020008914.1	c63925-62636	114360	1290
ERV-Spuma.83-Sca	CACTIS020008926.1:284396-285475	CACTIS020008926.1	284396-285475	288275	1080
ERV-Spuma.84-Sca	CACTIS020008926.1:c49495-48338	CACTIS020008926.1	c49495-48338	288275	1158
ERV-Spuma.85-Sca	CACTIS020008926.1:c310380-307459	CACTIS020008926.1	c310380-307459	288275	2922
ERV-Spuma.86-Sca	CACTIS020008926.1:c318808-315887	CACTIS020008926.1	c318808-315887	288275	2922
ERV-Spuma.87-Sca	CACTIS020009030.1:44569-45621	CACTIS020009030.1	44569-45621	480350	1053
ERV-Spuma.88-Sca	CACTIS020009030.1:45632-47479	CACTIS020009030.1	45632-47479	480350	1848
ERV-Spuma.89-Sca	CACTIS020009030.1:478056-479156	CACTIS020009030.1	478056-479156	480350	1101
ERV-Spuma.90-Sca	CACTIS020009039.1:11790-14711	CACTIS020009039.1	11790-14711	13923	2922
ERV-Spuma.91-Sca	CACTIS020009425.1:35432-38353	CACTIS020009425.1	35432-38353	37076	2922
ERV-Spuma.92-Sca	CACTIS020009425.1:43716-46430	CACTIS020009425.1	43716-46430	37076	2715
ERV-Spuma.93-Sca	CACTIS020009501.1:c161375-159072	CACTIS020009501.1	c161375-159072	254879	2304
ERV-Spuma.94-Sca	CACTIS020009607.1:13292-16213	CACTIS020009607.1	13292-16213	358622	2922
ERV-Spuma.95-Sca	CACTIS020009607.1:c33356-32001	CACTIS020009607.1	c33356-32001	358622	1356
ERV-Spuma.96-Sca	CACTIS020009607.1:c154267-152351	CACTIS020009607.1	c154267-152351	358622	1917
ERV-Spuma.97-Sca	CACTIS020009647.1:400773-401738	CACTIS020009647.1	400773-401738	404054	966
ERV-Spuma.98-Sca	CACTIS020009647.1:c12551-11583	CACTIS020009647.1	c12551-11583	404054	969
ERV-Spuma.99-Sca	CACTIS020009647.1:c66226-64865	CACTIS020009647.1	c66226-64865	404054	1362
ERV-Spuma.100-Sca	CACTIS020009647.1:c435978-433435	CACTIS020009647.1	c435978-433435	404054	2544
ERV-Spuma.101-Sca	CACTIS020010018.1:715446-717173	CACTIS020010018.1	715446-717173	720725	1728
ERV-Spuma.102-Sca	CACTIS020010018.1:717235-718365	CACTIS020010018.1	717235-718365	720725	1131
ERV-Spuma.103-Sca	CACTIS020010213.1:87917-89764	CACTIS020010213.1	87917-89764	92020	1848
ERV-Spuma.104-Sca	CACTIS020010352.1:49983-52904	CACTIS020010352.1	49983-52904	55389	2922
ERV-Spuma.105-Sca	CACTIS020010610.1:86872-88191	CACTIS020010610.1	86872-88191	246995	1320
ERV-Spuma.106-Sca	CACTIS020010610.1:c131841-130522	CACTIS020010610.1	c131841-130522	246995	1320
ERV-Spuma.107-Sca	CACTIS020010610.1:c159428-156507	CACTIS020010610.1	c159428-156507	246995	2922
ERV-Spuma.108-Sca	CACTIS020010874.1:c93965-92907	CACTIS020010874.1	c93965-92907	100504	1059
ERV-Spuma.109-Sca	CACTIS020010874.1:c95823-93946	CACTIS020010874.1	c95823-93946	100504	1878
ERV-Spuma.110-Sca	CACTIS020010907.1:c131470-129503	CACTIS020010907.1	c131470-129503	135117	1968
ERV-Spuma.111-Sca	CACTIS020011020.1:c69861-68872	CACTIS020011020.1	c69861-68872	541884	990
ERV-Spuma.112-Sca	CACTIS020011020.1:c508490-505692	CACTIS020011020.1	c508490-505692	541884	2799
ERV-Spuma.113-Sca	CACTIS020011305.1:c596287-593366	CACTIS020011305.1	c596287-593366	599934	2922
ERV-Spuma.114-Sca	CACTIS020011639.1:c469785-466864	CACTIS020011639.1	c469785-466864	544542	2922
ERV-Spuma.115-Sca	CACTIS020011639.1:c523367-522396	CACTIS020011639.1	c523367-522396	544542	972
ERV-Spuma.116-Sca	CACTIS020011821.1:54772-57693	CACTIS020011821.1	54772-57693	60185	2922
ERV-Spuma.117-Sca	CACTIS020012111.1:312311-315232	CACTIS020012111.1	312311-315232	554079	2922
ERV-Spuma.118-Sca	CACTIS020012164.1:c45968-43953	CACTIS020012164.1	c45968-43953	64722	2016
ERV-Spuma.119-Sca	CACTIS020012164.1:c46875-45898	CACTIS020012164.1	c46875-45898	64722	978
ERV-Spuma.120-Sca	CACTIS020012183.1:254564-256684	CACTIS020012183.1	254564-256684	226241	2121
ERV-Spuma.121-Sca	CACTIS020012884.1:153966-155117	CACTIS020012884.1	153966-155117	213548	1152
ERV-Spuma.122-Sca	CACTIS020013108.1:214720-216453	CACTIS020013108.1	214720-216453	518291	1734
ERV-Spuma.123-Sca	CACTIS020013108.1:216414-217640	CACTIS020013108.1	216414-217640	518291	1227
ERV-Spuma.124-Sca	CACTIS020013108.1:c140096-138933	CACTIS020013108.1	c140096-138933	518291	1164
ERV-Spuma.125-Sca	CACTIS020013108.1:c141727-140357	CACTIS020013108.1	c141727-140357	518291	1371
ERV-Spuma.126-Sca	CACTIS020013165.1:c24405-21484	CACTIS020013165.1	c24405-21484	28339	2922
ERV-Spuma.127-Sca	CACTIS020013330.1:c15471-13168	CACTIS020013330.1	c15471-13168	13396	2304
ERV-Spuma.128-Sca	CACTIS020013629.1:c10502-7581	CACTIS020013629.1	c10502-7581	14430	2922
ERV-Spuma.129-Sca	CACTIS020013674.1:448073-450799	CACTIS020013674.1	448073-450799	454074	2727
ERV-Spuma.130-Sca	CACTIS020013714.1:c96032-93111	CACTIS020013714.1	c96032-93111	94791	2922
ERV-Spuma.131-Sca	CACTIS020013863.1:22596-24506	CACTIS020013863.1	22596-24506	28050	1911
ERV-Spuma.132-Sca	CACTIS020013863.1:24454-25518	CACTIS020013863.1	24454-25518	28050	1065
ERV-Spuma.133-Sca	CACTIS020013997.1:c42341-39420	CACTIS020013997.1	c42341-39420	45955	2922
ERV-Spuma.134-Sca	CACTIS020014077.1:5869-7419	CACTIS020014077.1	5869-7419	11727	1551
ERV-Spuma.135-Sca	CACTIS020014077.1:7380-8606	CACTIS020014077.1	7380-8606	11727	1227
ERV-Spuma.136-Sca	CACTIS020014124.1:402023-404944	CACTIS020014124.1	402023-404944	407825	2922
ERV-Spuma.137-Sca	CACTIS020014169.1:c55551-52753	CACTIS020014169.1	c55551-52753	59165	2799
ERV-Spuma.138-Sca	CACTIS020014208.1:3719-5587	CACTIS020014208.1	3719-5587	367450	1869
ERV-Spuma.139-Sca	CACTIS020014557.1:c93716-92553	CACTIS020014557.1	c93716-92553	284336	1164
ERV-Spuma.140-Sca	CACTIS020014557.1:c126108-124819	CACTIS020014557.1	c126108-124819	284336	1290
ERV-Spuma.141-Sca	CACTIS020014557.1:c187267-185378	CACTIS020014557.1	c187267-185378	284336	1890
ERV-Spuma.142-Sca	CACTIS020014557.1:c199907-198030	CACTIS020014557.1	c199907-198030	284336	1878
ERV-Spuma.143-Sca	CACTIS020014631.1:c102702-99781	CACTIS020014631.1	c102702-99781	106345	2922
ERV-Spuma.144-Sca	CACTIS020014675.1:140252-143173	CACTIS020014675.1	140252-143173	145675	2922
ERV-Spuma.145-Sca	CACTIS020014675.1:c22211-20922	CACTIS020014675.1	c22211-20922	145675	1290
ERV-Spuma.146-Sca	CACTIS020014916.1:c154975-152672	CACTIS020014916.1	c154975-152672	159463	2304

ERV-Spuma.147-Sca	CACTIS020015004.1:c132829-130697	CACTIS020015004.1	c132829-130697	349399	2133
ERV-Spuma.148-Sca	CACTIS020015004.1:c346231-345152	CACTIS020015004.1	c346231-345152	349399	1080
ERV-Spuma.149-Sca	CACTIS020015160.1:c76521-74224	CACTIS020015160.1	c76521-74224	75094	2298
ERV-Spuma.150-Sca	CACTIS020015519.1:c161503-159941	CACTIS020015519.1	c161503-159941	254796	1563
ERV-Spuma.151-Sca	CACTIS020015524.1:24918-27839	CACTIS020015524.1	24918-27839	250793	2922
ERV-Spuma.152-Sca	CACTIS020015524.1:301356-302567	CACTIS020015524.1	301356-302567	250793	1212
ERV-Spuma.153-Sca	CACTIS020015528.1:41242-43911	CACTIS020015528.1	41242-43911	43360	2670
ERV-Spuma.154-Sca	CACTIS020015689.1:122534-125455	CACTIS020015689.1	122534-125455	127684	2922
ERV-Spuma.155-Sca	CACTIS020015748.1:24719-26008	CACTIS020015748.1	24719-26008	48966	1290
ERV-Spuma.156-Sca	CACTIS020015748.1:c45319-42398	CACTIS020015748.1	c45319-42398	48966	2922
ERV-Spuma.157-Sca	CACTIS020015767.1:204323-206212	CACTIS020015767.1	204323-206212	302226	1890
ERV-Spuma.158-Sca	CACTIS020015767.1:296904-299825	CACTIS020015767.1	296904-299825	302226	2922
ERV-Spuma.159-Sca	CACTIS020015838.1:190566-191780	CACTIS020015838.1	190566-191780	262819	1215
ERV-Spuma.160-Sca	CACTIS020015838.1:257683-260604	CACTIS020015838.1	257683-260604	262819	2922
ERV-Spuma.161-Sca	CACTIS020015870.1:180652-181941	CACTIS020015870.1	180652-181941	217286	1290
ERV-Spuma.162-Sca	CACTIS020016067.1:c3815-894	CACTIS020016067.1	c3815-894	1770	2922
ERV-Spuma.163-Sca	CACTIS020016081.1:c114560-113133	CACTIS020016081.1	c114560-113133	116880	1428
ERV-Spuma.164-Sca	CACTIS020016138.1:21944-23071	CACTIS020016138.1	21944-23071	24019	1128
ERV-Spuma.165-Sca	CACTIS020016138.1:c37396-35969	CACTIS020016138.1	c37396-35969	24019	1428
ERV-Spuma.166-Sca	CACTIS020016138.1:c38916-37414	CACTIS020016138.1	c38916-37414	24019	1503
ERV-Spuma.167-Sca	CACTIS020016605.1:c69850-67148	CACTIS020016605.1	c69850-67148	73978	2703
ERV-Spuma.168-Sca	CACTIS020016609.1:96644-98785	CACTIS020016609.1	96644-98785	108280	2142
ERV-Spuma.169-Sca	CACTIS020016679.1:c63105-60184	CACTIS020016679.1	c63105-60184	66994	2922
ERV-Spuma.170-Sca	CACTIS020016859.1:3660-4712	CACTIS020016859.1	3660-4712	5780	1053
ERV-Spuma.171-Sca	CACTIS020016859.1:5141-6430	CACTIS020016859.1	5141-6430	5780	1290
ERV-Spuma.172-Sca	CACTIS020016880.1:118891-121812	CACTIS020016880.1	118891-121812	91537	2922
ERV-Spuma.173-Sca	CACTIS020017066.1:c191096-188175	CACTIS020017066.1	c191096-188175	195066	2922
ERV-Spuma.174-Sca	CACTIS020017180.1:c25551-24460	CACTIS020017180.1	c25551-24460	57580	1092
ERV-Spuma.175-Sca	CACTIS020017180.1:c30915-29824	CACTIS020017180.1	c30915-29824	57580	1092
ERV-Spuma.176-Sca	CACTIS020017496.1:124435-125754	CACTIS020017496.1	124435-125754	130758	1320
ERV-Spuma.177-Sca	CACTIS020017496.1:c17989-16427	CACTIS020017496.1	c17989-16427	130758	1563
ERV-Spuma.178-Sca	CACTIS020017586.1:60516-63437	CACTIS020017586.1	60516-63437	65865	2922
ERV-Spuma.179-Sca	CACTIS020017660.1:c119961-118669	CACTIS020017660.1	c119961-118669	216932	1293
ERV-Spuma.180-Sca	CACTIS020017928.1:137833-140754	CACTIS020017928.1	137833-140754	139477	2922
ERV-Spuma.181-Sca	CACTIS020017934.1:56671-59592	CACTIS020017934.1	56671-59592	143329	2922
ERV-Spuma.182-Sca	CACTIS020018036.1:c108107-105186	CACTIS020018036.1	c108107-105186	112004	2922
ERV-Spuma.183-Sca	CACTIS020018056.1:74705-75676	CACTIS020018056.1	74705-75676	92732	972
ERV-Spuma.184-Sca	CACTIS020018056.1:87314-90235	CACTIS020018056.1	87314-90235	92732	2922
ERV-Spuma.185-Sca	CACTIS020018133.1:c31144-29054	CACTIS020018133.1	c31144-29054	31182	2091
ERV-Spuma.186-Sca	CACTIS020018152.1:c123272-120351	CACTIS020018152.1	c123272-120351	126919	2922
ERV-Spuma.187-Sca	CACTIS020018152.1:c136087-133394	CACTIS020018152.1	c136087-133394	126919	2694
ERV-Spuma.188-Sca	CACTIS020018175.1:c38850-35929	CACTIS020018175.1	c38850-35929	42464	2922
ERV-Spuma.189-Sca	CACTIS020018188.1:56916-59006	CACTIS020018188.1	56916-59006	58203	2091
ERV-Spuma.190-Sca	CACTIS020018240.1:144683-147604	CACTIS020018240.1	144683-147604	149146	2922
ERV-Spuma.191-Sca	CACTIS020018343.1:c12361-9443	CACTIS020018343.1	c12361-9443	10319	2919
ERV-Spuma.192-Sca	CACTIS020018706.1:61690-62901	CACTIS020018706.1	61690-62901	63911	1212
ERV-Spuma.193-Sca	CACTIS020018706.1:63473-64597	CACTIS020018706.1	63473-64597	63911	1125
ERV-Spuma.194-Sca	CACTIS020018720.1:140785-142962	CACTIS020018720.1	140785-142962	94615	2178
ERV-Spuma.195-Sca	CACTIS020018723.1:64282-67203	CACTIS020018723.1	64282-67203	69541	2922
ERV-Spuma.196-Sca	CACTIS020018739.1:27546-28709	CACTIS020018739.1	27546-28709	32932	1164
ERV-Spuma.197-Sca	CACTIS020018739.1:28721-30466	CACTIS020018739.1	28721-30466	32932	1746
ERV-Spuma.198-Sca	CACTIS020019005.1:c114539-111618	CACTIS020019005.1	c114539-111618	112743	2922
ERV-Spuma.199-Sca	CACTIS020019157.1:37344-39737	CACTIS020019157.1	37344-39737	44999	2394
ERV-Spuma.200-Sca	CACTIS020019157.1:c67217-64362	CACTIS020019157.1	c67217-64362	44999	2856
ERV-Spuma.201-Sca	CACTIS020019354.1:75819-78179	CACTIS020019354.1	75819-78179	81019	2361
ERV-Spuma.202-Sca	CACTIS020019385.1:c19865-16944	CACTIS020019385.1	c19865-16944	23693	2922
ERV-Spuma.203-Sca	CACTIS020019402.1:c136999-134078	CACTIS020019402.1	c136999-134078	140959	2922
ERV-Spuma.204-Sca	CACTIS020019507.1:25504-27396	CACTIS020019507.1	25504-27396	30693	1893
ERV-Spuma.205-Sca	CACTIS020019507.1:27300-28349	CACTIS020019507.1	27300-28349	30693	1050
ERV-Spuma.206-Sca	CACTIS020019950.1:77287-79566	CACTIS020019950.1	77287-79566	128648	2280
ERV-Spuma.207-Sca	CACTIS020019978.1:27289-28866	CACTIS020019978.1	27289-28866	80562	1578
ERV-Spuma.208-Sca	CACTIS020019978.1:28938-30209	CACTIS020019978.1	28938-30209	80562	1272
ERV-Spuma.209-Sca	CACTIS020019984.1:12069-14990	CACTIS020019984.1	12069-14990	78630	2922
ERV-Spuma.210-Sca	CACTIS020019984.1:72345-74234	CACTIS020019984.1	72345-74234	78630	1890
ERV-Spuma.211-Sca	CACTIS020020290.1:24179-27100	CACTIS020020290.1	24179-27100	26318	2922
ERV-Spuma.212-Sca	CACTIS020020290.1:32597-35518	CACTIS020020290.1	32597-35518	26318	2922
ERV-Spuma.213-Sca	CACTIS020020462.1:99418-102120	CACTIS020020462.1	99418-102120	104391	2703
ERV-Spuma.214-Sca	CACTIS020020679.1:c112339-110996	CACTIS020020679.1	c112339-110996	111011	1344
ERV-Spuma.215-Sca	CACTIS020020829.1:c114310-111389	CACTIS020020829.1	c114310-111389	118419	2922
ERV-Spuma.216-Sca	CACTIS020021236.1:c61141-60062	CACTIS020021236.1	c61141-60062	60104	1080
ERV-Spuma.217-Sca	CACTIS020021236.1:c103290-101728	CACTIS020021236.1	c103290-101728	60104	1563
ERV-Spuma.218-Sca	CACTIS020021236.1:c104525-103410	CACTIS020021236.1	c104525-103410	60104	1116
ERV-Spuma.219-Sca	CACTIS020021389.1:69919-72219	CACTIS020021389.1	69919-72219	71794	2301
ERV-Spuma.220-Sca	CACTIS020021406.1:c9479-8415	CACTIS020021406.1	c9479-8415	8406	1065

ERV-Spuma.221-Sca	CACTIS020021560.1:c34998-32077	CACTIS020021560.1	c34998-32077	53223	2922
ERV-Spuma.222-Sca	CACTIS020021572.1:c71973-69052	CACTIS020021572.1	c71973-69052	75900	2922
ERV-Spuma.223-Sca	CACTIS020022124.1:c89223-87103	CACTIS020022124.1	c89223-87103	68633	2121
ERV-Spuma.224-Sca	CACTIS020022421.1:90978-92120	CACTIS020022421.1	90978-92120	93073	1143
ERV-Spuma.225-Sca	CACTIS020022421.1:92605-93876	CACTIS020022421.1	92605-93876	93073	1272
ERV-Spuma.226-Sca	CACTIS020022454.1:c20503-18722	CACTIS020022454.1	c20503-18722	18974	1782
ERV-Spuma.227-Sca	CACTIS020023086.1:64957-66009	CACTIS020023086.1	64957-66009	69649	1053
ERV-Spuma.228-Sca	CACTIS020023086.1:66081-67352	CACTIS020023086.1	66081-67352	69649	1272
ERV-Spuma.229-Sca	CACTIS020023393.1:c15860-13137	CACTIS020023393.1	c15860-13137	13815	2724
ERV-Spuma.230-Sca	CACTIS020023487.1:36336-38615	CACTIS020023487.1	36336-38615	36351	2280
ERV-Spuma.231-Sca	CACTIS020023487.1:c74548-73238	CACTIS020023487.1	c74548-73238	36351	1311
ERV-Spuma.232-Sca	CACTIS020023593.1:c21650-18729	CACTIS020023593.1	c21650-18729	24939	2922
ERV-Spuma.233-Sca	CACTIS020023831.1:26041-27528	CACTIS020023831.1	26041-27528	29799	1488
ERV-Spuma.234-Sca	CACTIS020024068.1:c39834-36913	CACTIS020024068.1	c39834-36913	38038	2922
ERV-Spuma.235-Sca	CACTIS020024294.1:c48434-46872	CACTIS020024294.1	c48434-46872	24194	1563
ERV-Spuma.236-Sca	CACTIS020024294.1:c49780-48569	CACTIS020024294.1	c49780-48569	24194	1212
ERV-Spuma.237-Sca	CACTIS020026534.1:c5376-2464	CACTIS020026534.1	c5376-2464	3808	2913
ERV-Spuma.238-Sca	CACTIT020000004.1:c152487-150598	CACTIT020000004.1	c152487-150598	399311	1890
ERV-Spuma.239-Sca	CACTIT020000004.1:c394159-392414	CACTIT020000004.1	c394159-392414	399311	1746
ERV-Spuma.240-Sca	CACTIT020000004.1:c395322-394171	CACTIT020000004.1	c395322-394171	399311	1152
ERV-Spuma.241-Sca	CACTIT020000018.1:242231-244108	CACTIT020000018.1	242231-244108	386050	1878
ERV-Spuma.242-Sca	CACTIT020000018.1:380797-383709	CACTIT020000018.1	380797-383709	386050	2913
ERV-Spuma.243-Sca	CACTIT020000018.1:c168866-166041	CACTIT020000018.1	c168866-166041	386050	2826
ERV-Spuma.244-Sca	CACTIT020000038.1:344048-346741	CACTIT020000038.1	344048-346741	2367138	2694
ERV-Spuma.245-Sca	CACTIT020000038.1:1324526-1327261	CACTIT020000038.1	1324526-1327261	2367138	2736
ERV-Spuma.246-Sca	CACTIT020000038.1:2361853-2364774	CACTIT020000038.1	2361853-2364774	2367138	2922
ERV-Spuma.247-Sca	CACTIT020000038.1:c1684806-1682917	CACTIT020000038.1	c1684806-1682917	2367138	1890
ERV-Spuma.248-Sca	CACTIT020000053.1:c36167-34077	CACTIT020000053.1	c36167-34077	36205	2091
ERV-Spuma.249-Sca	CACTIT020000067.1:c710661-708448	CACTIT020000067.1	c710661-708448	715341	2214
ERV-Spuma.250-Sca	CACTIT020000070.1:c119576-117699	CACTIT020000070.1	c119576-117699	123123	1878
ERV-Spuma.251-Sca	CACTIT020000070.1:c251186-250107	CACTIT020000070.1	c251186-250107	123123	1080
ERV-Spuma.252-Sca	CACTIT020000070.1:c1046453-1043532	CACTIT020000070.1	c1046453-1043532	123123	2922
ERV-Spuma.253-Sca	CACTIT020000070.1:c1059264-1057483	CACTIT020000070.1	c1059264-1057483	123123	1782
ERV-Spuma.254-Sca	CACTIT020000071.1:c488053-485132	CACTIT020000071.1	c488053-485132	492080	2922
ERV-Spuma.255-Sca	CACTIT020000077.1:3129888-3131795	CACTIT020000077.1	3129888-3131795	4429010	1908
ERV-Spuma.256-Sca	CACTIT020000077.1:c1657078-1655873	CACTIT020000077.1	c1657078-1655873	4429010	1206
ERV-Spuma.257-Sca	CACTIT020000077.1:c1714833-1713706	CACTIT020000077.1	c1714833-1713706	4429010	1128
ERV-Spuma.258-Sca	CACTIT020000077.1:c2080786-2079011	CACTIT020000077.1	c2080786-2079011	4429010	1776
ERV-Spuma.259-Sca	CACTIT020000094.1:28517-31243	CACTIT020000094.1	28517-31243	66565	2727
ERV-Spuma.260-Sca	CACTIT020000094.1:44818-47544	CACTIT020000094.1	44818-47544	66565	2727
ERV-Spuma.261-Sca	CACTIT020000094.1:61334-64255	CACTIT020000094.1	61334-64255	66565	2922
ERV-Spuma.262-Sca	CACTIT020000119.1:287817-289031	CACTIT020000119.1	287817-289031	111515	1215
ERV-Spuma.263-Sca	CACTIT020000119.1:670030-672951	CACTIT020000119.1	670030-672951	111515	2922
ERV-Spuma.264-Sca	CACTIT020000119.1:c598728-596953	CACTIT020000119.1	c598728-596953	111515	1776
ERV-Spuma.265-Sca	CACTIT020000196.1:14378-17326	CACTIT020000196.1	14378-17326	20036	2949
ERV-Spuma.266-Sca	CACTIT020000207.1:20543-23464	CACTIT020000207.1	20543-23464	25709	2922
ERV-Spuma.267-Sca	CACTIT020000214.1:35476-37737	CACTIT020000214.1	35476-37737	36949	2262
ERV-Spuma.268-Sca	CACTIT020000216.1:c9536-8295	CACTIT020000216.1	c9536-8295	10804	1242
ERV-Spuma.269-Sca	CACTIT020000230.1:31150-33618	CACTIT020000230.1	31150-33618	33025	2469
ERV-Spuma.270-Sca	CACTIT020000231.1:2422691-2425612	CACTIT020000231.1	2422691-2425612	2427840	2922
ERV-Spuma.271-Sca	CACTIT020000239.1:c6442-5393	CACTIT020000239.1	c6442-5393	6439	1050
ERV-Spuma.272-Sca	CACTIT020000239.1:c7641-6442	CACTIT020000239.1	c7641-6442	6439	1200
ERV-Spuma.273-Sca	CACTIT020000241.1:34147-35703	CACTIT020000241.1	34147-35703	35739	1557
ERV-Spuma.274-Sca	CACTIT020000294.1:2460911-2463649	CACTIT020000294.1	2460911-2463649	2465899	2739
ERV-Spuma.275-Sca	CACTIT020000294.1:c8630-7344	CACTIT020000294.1	c8630-7344	2465899	1287
ERV-Spuma.276-Sca	CACTIT020000294.1:c71874-70900	CACTIT020000294.1	c71874-70900	2465899	975
ERV-Spuma.277-Sca	CACTIT020000294.1:c1880548-1879259	CACTIT020000294.1	c1880548-1879259	2465899	1290
ERV-Spuma.278-Sca	CACTIT020000294.1:c2315349-2313466	CACTIT020000294.1	c2315349-2313466	2465899	1884
ERV-Spuma.279-Sca	CACTIT020000295.1:1710337-1713258	CACTIT020000295.1	1710337-1713258	1844008	2922
ERV-Spuma.280-Sca	CACTIT020000305.1:329024-330343	CACTIT020000305.1	329024-330343	4166639	1320
ERV-Spuma.281-Sca	CACTIT020000305.1:2444809-2447088	CACTIT020000305.1	2444809-2447088	4166639	2280
ERV-Spuma.282-Sca	CACTIT020000305.1:2469083-2470288	CACTIT020000305.1	2469083-2470288	4166639	1206
ERV-Spuma.283-Sca	CACTIT020000305.1:2470561-2471775	CACTIT020000305.1	2470561-2471775	4166639	1215
ERV-Spuma.284-Sca	CACTIT020000305.1:3146873-3148750	CACTIT020000305.1	3146873-3148750	4166639	1878
ERV-Spuma.285-Sca	CACTIT020000328.1:1149012-1150901	CACTIT020000328.1	1149012-1150901	1155296	1890
ERV-Spuma.286-Sca	CACTIT020000328.1:1539268-1540743	CACTIT020000328.1	1539268-1540743	1155296	1476
ERV-Spuma.287-Sca	CACTIT020000328.1:1540776-1542173	CACTIT020000328.1	1540776-1542173	1155296	1398
ERV-Spuma.288-Sca	CACTIT020000429.1:c788690-785769	CACTIT020000429.1	c788690-785769	792661	2922
ERV-Spuma.289-Sca	CACTIT020000477.1:c177682-175385	CACTIT020000477.1	c177682-175385	177059	2298
ERV-Spuma.290-Sca	CACTIT020000486.1:190941-192716	CACTIT020000486.1	190941-192716	250376	1776
ERV-Spuma.291-Sca	CACTIT020000486.1:c136771-134474	CACTIT020000486.1	c136771-134474	250376	2298
ERV-Spuma.292-Sca	CACTIT020000509.1:19141-22062	CACTIT020000509.1	19141-22062	24556	2922
ERV-Spuma.293-Sca	CACTIT020000579.1:c32349-29665	CACTIT020000579.1	c32349-29665	31108	2685
ERV-Spuma.1-Etr	JAAVTP020000019.1:10627827-10628816	JAAVTP020000019.1	10627827-10628816	11,387,455	990

ERV-Spuma.2-Etr	JAAVTP020000019.1:c10989414-10988110	JAAVTP020000019.1	c10989414-10988110	11,387,455	1305
ERV-Spuma.3-Etr	JAAVTP020000019.1:c11351127-11349874	JAAVTP020000019.1	c11351127-11349874	11,387,455	1254
ERV-Spuma.4-Etr	JAAVTP020000022.1:92725-93795	JAAVTP020000022.1	92725-93795	11,244,061	1071
ERV-Spuma.5-Etr	JAAVTP020000022.1:195023-196252	JAAVTP020000022.1	195023-196252	11,244,061	1230
ERV-Spuma.6-Etr	JAAVTP020000022.1:204064-205038	JAAVTP020000022.1	204064-205038	11,244,061	975
ERV-Spuma.7-Etr	JAAVTP020000022.1:333959-335275	JAAVTP020000022.1	333959-335275	11,244,061	1317
ERV-Spuma.8-Etr	JAAVTP020000022.1:c176692-175730	JAAVTP020000022.1	c176692-175730	11,244,061	963
ERV-Spuma.9-Etr	JAAVTP020000022.1:c183042-181951	JAAVTP020000022.1	c183042-181951	11,244,061	1092
ERV-Spuma.10-Etr	JAAVTP020000022.1:c6050779-6049514	JAAVTP020000022.1	c6050779-6049514	11,244,061	1266
ERV-Spuma.11-Etr	JAAVTP020000025.1:1290296-1291615	JAAVTP020000025.1	1290296-1291615	4079654	1320
ERV-Spuma.12-Etr	JAAVTP020000025.1:2070051-2071304	JAAVTP020000025.1	2070051-2071304	4079654	1254
ERV-Spuma.13-Etr	JAAVTP020000025.1:2099332-2100540	JAAVTP020000025.1	2099332-2100540	4079654	1209
ERV-Spuma.14-Etr	JAAVTP020000025.1:c1110415-1109114	JAAVTP020000025.1	c1110415-1109114	4079654	1302
ERV-Spuma.15-Etr	JAAVTP020000025.1:c1719436-1718432	JAAVTP020000025.1	c1719436-1718432	4079654	1005
ERV-Spuma.16-Etr	JAAVTP020000025.1:c1847857-1846895	JAAVTP020000025.1	c1847857-1846895	4079654	963
ERV-Spuma.17-Etr	JAAVTP020000025.1:c1855864-1854779	JAAVTP020000025.1	c1855864-1854779	4079654	1086
ERV-Spuma.18-Etr	JAAVTP020000025.1:c2093312-2092350	JAAVTP020000025.1	c2093312-2092350	4079654	963
ERV-Spuma.19-Etr	JAAVTP020000027.1:65609-66658	JAAVTP020000027.1	65609-66658	10,304,965	1050
ERV-Spuma.20-Etr	JAAVTP020000027.1:490603-491925	JAAVTP020000027.1	490603-491925	10,304,965	1323
ERV-Spuma.21-Etr	JAAVTP020000027.1:636941-637924	JAAVTP020000027.1	636941-637924	10,304,965	984
ERV-Spuma.22-Etr	JAAVTP020000027.1:c242605-241610	JAAVTP020000027.1	c242605-241610	10,304,965	996
ERV-Spuma.23-Etr	JAAVTP020000045.1:131210-132391	JAAVTP020000045.1	131210-132391	7231711	1182
ERV-Spuma.24-Etr	JAAVTP020000045.1:650890-651876	JAAVTP020000045.1	650890-651876	7231711	987
ERV-Spuma.25-Etr	JAAVTP020000045.1:1822969-1824177	JAAVTP020000045.1	1822969-1824177	7231711	1209
ERV-Spuma.26-Etr	JAAVTP020000045.1:c232202-231024	JAAVTP020000045.1	c232202-231024	7231711	1179
ERV-Spuma.27-Etr	JAAVTP020000045.1:c502957-501767	JAAVTP020000045.1	c502957-501767	7231711	1191
ERV-Spuma.28-Etr	JAAVTP020000045.1:c701022-699700	JAAVTP020000045.1	c701022-699700	7231711	1323
ERV-Spuma.29-Etr	JAAVTP020000049.1:452626-453888	JAAVTP020000049.1	452626-453888	1967377	1263
ERV-Spuma.30-Etr	JAAVTP020000049.1:808314-809351	JAAVTP020000049.1	808314-809351	1967377	1038
ERV-Spuma.31-Etr	JAAVTP020000049.1:1071276-1072592	JAAVTP020000049.1	1071276-1072592	1967377	1317
ERV-Spuma.32-Etr	JAAVTP020000049.1:2347313-2348299	JAAVTP020000049.1	2347313-2348299	1967377	987
ERV-Spuma.33-Etr	JAAVTP020000049.1:c1964611-1963289	JAAVTP020000049.1	c1964611-1963289	1967377	1323
ERV-Spuma.34-Etr	JAAVTP020000067.1:1191420-1192424	JAAVTP020000067.1	1191420-1192424	1,495,792	1005
ERV-Spuma.35-Etr	JAAVTP020000067.1:c1359145-1358138	JAAVTP020000067.1	c1359145-1358138	1,495,792	1008
ERV-Spuma.36-Etr	JAAVTP020000069.1:c32109-31135	JAAVTP020000069.1	c32109-31135	1,387,424	975
ERV-Spuma.37-Etr	JAAVTP020000081.1:104436-105737	JAAVTP020000081.1	104436-105737	759,291	1302
ERV-Spuma.38-Etr	JAAVTP020000081.1:255414-256418	JAAVTP020000081.1	255414-256418	759,291	1005
ERV-Spuma.39-Etr	JAAVTP020000081.1:290952-292217	JAAVTP020000081.1	290952-292217	759,291	1266
ERV-Spuma.40-Etr	JAAVTP020000081.1:c220321-219017	JAAVTP020000081.1	c220321-219017	759,291	1305
ERV-Spuma.41-Etr	JAAVTP020000102.1:c131424-130102	JAAVTP020000102.1	c131424-130102	42369	1323
ERV-Spuma.42-Etr	JAAVTP020000229.1:70078-71064	JAAVTP020000229.1	70078-71064	104,201	987
ERV-Spuma.43-Etr	JAAVTP020000229.1:c85431-84196	JAAVTP020000229.1	c85431-84196	104,201	1236
ERV-Spuma.44-Etr	JAAVTP020000249.1:85366-86397	JAAVTP020000249.1	85366-86397	90,162	1032
ERV-Spuma.45-Etr	JAAVTP020000249.1:c60097-58835	JAAVTP020000249.1	c60097-58835	90,162	1263
ERV-Spuma.46-Etr	JAAVTP020002587.1:c6603-5617	JAAVTP020002587.1	c6603-5617	11,830	987
ERV-Spuma.47-Etr	JAAVTP020003269.1:6929-7927	JAAVTP020003269.1	6929-7927	10,305	999
ERV-Spuma.48-Etr	JAAVTP020013941.1:c1508-516	JAAVTP020013941.1	c1508-516	3,990	993
ERV-Spuma.49-Etr	JAAXLI020001155.1:650534-651496	JAAXLI020001155.1	650534-651496	12,457,464	963
ERV-Spuma.50-Etr	JAAXLI020001155.1:c1110007-1108820	JAAXLI020001155.1	c1110007-1108820	12,457,464	1188
ERV-Spuma.51-Etr	JAAXLI020001155.1:c3394714-3393455	JAAXLI020001155.1	c3394714-3393455	12,457,464	1260
ERV-Spuma.52-Etr	JAAXLI020001166.1:999868-1001133	JAAXLI020001166.1	999868-1001133	4308926	1266
ERV-Spuma.53-Etr	JAAXLI020001166.1:1296837-1298156	JAAXLI020001166.1	1296837-1298156	4308926	1320
ERV-Spuma.54-Etr	JAAXLI020001166.1:2036114-2037379	JAAXLI020001166.1	2036114-2037379	4308926	1266
ERV-Spuma.55-Etr	JAAXLI020001166.1:2117284-2118492	JAAXLI020001166.1	2117284-2118492	4308926	1209
ERV-Spuma.56-Etr	JAAXLI020001166.1:c1713442-1712438	JAAXLI020001166.1	c1713442-1712438	4308926	1005
ERV-Spuma.57-Etr	JAAXLI020001166.1:c1846228-1845266	JAAXLI020001166.1	c1846228-1845266	4308926	963
ERV-Spuma.58-Etr	JAAXLI020001166.1:c1854304-1853219	JAAXLI020001166.1	c1854304-1853219	4308926	1086
ERV-Spuma.59-Etr	JAAXLI020001166.1:c2111264-2110302	JAAXLI020001166.1	c2111264-2110302	4308926	963
ERV-Spuma.60-Etr	JAAXLI020001166.1:c4063892-4062591	JAAXLI020001166.1	c4063892-4062591	4308926	1302
ERV-Spuma.61-Etr	JAAXLI020001169.1:c574323-573328	JAAXLI020001169.1	c574323-573328	10,891,510	996
ERV-Spuma.62-Etr	JAAXLI020001169.1:c655565-654543	JAAXLI020001169.1	c655565-654543	10,891,510	1023
ERV-Spuma.63-Etr	JAAXLI020001182.1:c4793-3456	JAAXLI020001182.1	c4793-3456	8,241,887	1338
ERV-Spuma.64-Etr	JAAXLI020001185.1:88250-89452	JAAXLI020001185.1	88250-89452	7,916,794	1203
ERV-Spuma.65-Etr	JAAXLI020001185.1:322881-324206	JAAXLI020001185.1	322881-324206	7,916,794	1326
ERV-Spuma.66-Etr	JAAXLI020001185.1:786895-787881	JAAXLI020001185.1	786895-787881	7,916,794	987
ERV-Spuma.67-Etr	JAAXLI020001185.1:1956007-1957239	JAAXLI020001185.1	1956007-1957239	7,916,794	1233
ERV-Spuma.68-Etr	JAAXLI020001185.1:c404763-403585	JAAXLI020001185.1	c404763-403585	7,916,794	1179
ERV-Spuma.69-Etr	JAAXLI020001185.1:c638929-637739	JAAXLI020001185.1	c638929-637739	7,916,794	1191
ERV-Spuma.70-Etr	JAAXLI020001185.1:c862747-861425	JAAXLI020001185.1	c862747-861425	7,916,794	1323
ERV-Spuma.71-Etr	JAAXLI020001185.1:c1928779-1927805	JAAXLI020001185.1	c1928779-1927805	7,916,794	975
ERV-Spuma.72-Etr	JAAXLI020001197.1:1112577-1113551	JAAXLI020001197.1	1112577-1113551	2,559,654	975
ERV-Spuma.73-Etr	JAAXLI020001197.1:c585339-584242	JAAXLI020001197.1	c585339-584242	2,559,654	1098
ERV-Spuma.74-Etr	JAAXLI020001197.1:c832857-831610	JAAXLI020001197.1	c832857-831610	2,559,654	1248
ERV-Spuma.75-Etr	JAAXLI020001197.1:c2099795-2098524	JAAXLI020001197.1	c2099795-2098524	2,559,654	1272

ERV-Spuma.76-Etr	JAAXLI020001205.1:104931-105938	JAAXLI020001205.1	104931-105938	1,292,846	1008
ERV-Spuma.77-Etr	JAAXLI020001205.1:1189895-1190899	JAAXLI020001205.1	1189895-1190899	1,292,846	1005
ERV-Spuma.78-Etr	JAAXLI020001205.1:c1064252-1063233	JAAXLI020001205.1	c1064252-1063233	1,292,846	1020
ERV-Spuma.79-Etr	JAAXLI020001226.1:313909-315231	JAAXLI020001226.1	313909-315231	315934	1323
ERV-Spuma.80-Etr	JAAXLI020001241.1:38084-39373	JAAXLI020001241.1	38084-39373	332050	1290
ERV-Spuma.81-Etr	JAAXLI020001241.1:328041-329027	JAAXLI020001241.1	328041-329027	332050	987
ERV-Spuma.82-Etr	JAAXLI020001241.1:c48204-47131	JAAXLI020001241.1	c48204-47131	332050	1074
ERV-Spuma.83-Etr	JAAXLI020001530.1:c6401-5193	JAAXLI020001530.1	c6401-5193	31,851	1209
ERV-Spuma.84-Etr	JAAXLI020001714.1:c15934-14933	JAAXLI020001714.1	c15934-14933	20,270	1002
ERV-Spuma.85-Etr	JAAXLI020001731.1:5120-6109	JAAXLI020001731.1	5120-6109	19,825	990
ERV-Spuma.86-Etr	JAAXLI020002595.1:6306-7331	JAAXLI020002595.1	6306-7331	12,810	1026
ERV-Spuma.87-Etr	JAAXLI020002687.1:9057-10043	JAAXLI020002687.1	9057-10043	12,473	987
ERV-Spuma.88-Etr	JAAXLI020005802.1:c2520-1540	JAAXLI020005802.1	c2520-1540	8,038	981
ERV-Spuma.89-Etr	JAAXLI020009800.1:2111-3094	JAAXLI020009800.1	2111-3094	5,435	984
ERV-Spuma.90-Etr	JAAXLI020010138.1:2343-3338	JAAXLI020010138.1	2343-3338	5,274	996
ERV-Spuma.91-Etr	JAAXLI020012139.1:1156-2142	JAAXLI020012139.1	1156-2142	4,439	987
ERV-Spuma.1-Ebu	FYBX02010718.1:4187750-4188856	FYBX02010718.1	4187750-4188856	1520647	1107
ERV-Spuma.1-Lca	APJL01023490.1:18005-18967	APJL01023490.1	18005-18967	18,968	963
ERV-Spuma.2-Lca	APJL01032512.1:2363-3367	APJL01032512.1	2363-3367	4,134	1005
ERV-Spuma.3-Lca	APJL01036253.1:c1005-1	APJL01036253.1	c1005-1	1,571	1005
ERV-Spuma.4-Lca	APJL01041016.1:2825-3829	APJL01041016.1	2825-3829	6,042	1005
ERV-Spuma.5-Lca	APJL01046347.1:2823-4031	APJL01046347.1	2823-4031	12,553	1209
ERV-Spuma.6-Lca	APJL01046347.1:c11013-10021	APJL01046347.1	c11013-10021	12,553	993
ERV-Spuma.7-Lca	APJL01049445.1:c20751-19765	APJL01049445.1	c20751-19765	28,663	987
ERV-Spuma.8-Lca	APJL01053917.1:c4623-3595	APJL01053917.1	c4623-3595	8,184	1029
ERV-Spuma.9-Lca	APJL01063301.1:c1449-253	APJL01063301.1	c1449-253	17,542	1197
ERV-Spuma.10-Lca	APJL01063301.1:c13785-12790	APJL01063301.1	c13785-12790	17,542	996
ERV-Spuma.11-Lca	APJL01066204.1:4831-5826	APJL01066204.1	4831-5826	9,362	996
ERV-Spuma.12-Lca	APJL01076692.1:c10563-9529	APJL01076692.1	c10563-9529	10,803	1035
ERV-Spuma.13-Lca	APJL01092180.1:c3167-2127	APJL01092180.1	c3167-2127	6,573	1041
ERV-Spuma.14-Lca	APJL01093716.1:c4776-3784	APJL01093716.1	c4776-3784	11,713	993
ERV-Spuma.15-Lca	APJL01099496.1:c1122-133	APJL01099496.1	c1122-133	4,578	990
ERV-Spuma.16-Lca	APJL01101315.1:c1536-562	APJL01101315.1	c1536-562	1823	975
ERV-Spuma.17-Lca	APJL01104703.1:278-1423	APJL01104703.1	278-1423	3,297	1146
ERV-Spuma.18-Lca	APJL01110781.1:1580-2545	APJL01110781.1	1580-2545	2,553	966
ERV-Spuma.19-Lca	APJL01114069.1:1213-2205	APJL01114069.1	1213-2205	2,314	993
ERV-Spuma.20-Lca	APJL01133636.1:620-1597	APJL01133636.1	620-1597	1,605	978
ERV-Spuma.21-Lca	APJL01137688.1:543-1517	APJL01137688.1	543-1517	1,519	975
ERV-Spuma.22-Lca	APJL01141850.1:c1122-118	APJL01141850.1	c1122-118	1,441	1005
ERV-Spuma.23-Lca	APJL01144395.1:3-1070	APJL01144395.1	3-1070	1,397	1068
ERV-Spuma.24-Lca	APJL01145357.1:c1062-70	APJL01145357.1	c1062-70	1,382	993
ERV-Spuma.25-Lca	WFAB01000017.1:9101-10087	WFAB01000017.1	9101-10087	121,062	987
ERV-Spuma.26-Lca	WFAB01000072.1:882217-883299	WFAB01000072.1	882217-883299	1,947,535	1083
ERV-Spuma.27-Lca	WFAB01000072.1:c249452-248421	WFAB01000072.1	c249452-248421	1,947,535	1032
ERV-Spuma.28-Lca	WFAB01000088.1:c4729371-4728388	WFAB01000088.1	c4729371-4728388	14,750,739	984
ERV-Spuma.29-Lca	WFAB01000088.1:c13807429-13806458	WFAB01000088.1	c13807429-13806458	14,750,739	972
ERV-Spuma.30-Lca	WFAB01000192.1:4397862-4399163	WFAB01000192.1	4397862-4399163	4401276	1302
ERV-Spuma.31-Lca	WFAB01000192.1:c1689506-1688505	WFAB01000192.1	c1689506-1688505	4401276	1002
ERV-Spuma.32-Lca	WFAB01000192.1:c2351294-2350308	WFAB01000192.1	c2351294-2350308	4401276	987
ERV-Spuma.33-Lca	WFAB01000193.1:c15447-14092	WFAB01000193.1	c15447-14092	2263890	1356
ERV-Spuma.34-Lca	WFAB01000193.1:c1605025-1603991	WFAB01000193.1	c1605025-1603991	2263890	1035
ERV-Spuma.35-Lca	WFAB01000209.1:c1039527-1038487	WFAB01000209.1	c1039527-1038487	1,495,787	1041
ERV-Spuma.36-Lca	WFAB01000248.1:c8091397-8090396	WFAB01000248.1	c8091397-8090396	8,198,886	1002
ERV-Spuma.37-Lca	WFAB01000272.1:c116359-115373	WFAB01000272.1	c116359-115373	124,309	987
ERV-Spuma.38-Lca	WFAB01000273.1:c37672-36686	WFAB01000273.1	c37672-36686	1,105,953	987
ERV-Spuma.39-Lca	WFAB01000336.1:186839-187888	WFAB01000336.1	186839-187888	552,514	1050
ERV-Spuma.40-Lca	WFAB01000375.1:14920-16215	WFAB01000375.1	14920-16215	894,977	1296
ERV-Spuma.41-Lca	WFAB01000375.1:c214657-213665	WFAB01000375.1	c214657-213665	894,977	993
ERV-Spuma.42-Lca	WFAB01000449.1:4422410-4423396	WFAB01000449.1	4422410-4423396	5,481,687	987
ERV-Spuma.43-Lca	WFAB01000474.1:c1901118-1900126	WFAB01000474.1	c1901118-1900126	2,698,218	993
ERV-Spuma.44-Lca	WFAB01000603.1:c2339679-2338645	WFAB01000603.1	c2339679-2338645	4,597,781	1035
ERV-Spuma.45-Lca	WFAB01000666.1:23574-24815	WFAB01000666.1	23574-24815	178,220	1242
ERV-Spuma.46-Lca	WFAB01000666.1:c81451-80195	WFAB01000666.1	c81451-80195	178,220	1257
ERV-Spuma.47-Lca	WFAB01000678.1:79440-80759	WFAB01000678.1	79440-80759	26941	1320
ERV-Spuma.48-Lca	WFAB01000883.1:391086-392384	WFAB01000883.1	391086-392384	396,157	1299
ERV-Spuma.49-Lca	WFAB01000916.1:369771-370769	WFAB01000916.1	369771-370769	624157	999
ERV-Spuma.50-Lca	WFAB01000916.1:431620-432873	WFAB01000916.1	431620-432873	624157	1254
ERV-Spuma.51-Lca	WFAB01000916.1:620122-621384	WFAB01000916.1	620122-621384	624157	1263
ERV-Spuma.52-Lca	WFAB01000916.1:711936-713405	WFAB01000916.1	711936-713405	624157	1470
ERV-Spuma.53-Lca	WFAB01000919.1:c93883-92897	WFAB01000919.1	c93883-92897	565682	987
ERV-Spuma.54-Lca	WFAB01000919.1:c238056-236761	WFAB01000919.1	c238056-236761	565682	1296
ERV-Spuma.55-Lca	WFAB01000948.1:136575-137558	WFAB01000948.1	136575-137558	346,506	984
ERV-Spuma.56-Lca	WFAB01000987.1:64428-65474	WFAB01000987.1	64428-65474	1283737	1047
ERV-Spuma.57-Lca	WFAB01000987.1:420472-421461	WFAB01000987.1	420472-421461	1283737	990

ERV-Spuma.58-Lca	WFAB01000989.1:59612-60607	WFAB01000989.1	59612-60607	242295	996
ERV-Spuma.59-Lca	WFAB01000989.1:c19644-18661	WFAB01000989.1	c19644-18661	242295	984
ERV-Spuma.60-Lca	WFAB01001028.1:30816-31808	WFAB01001028.1	30816-31808	502,597	993
ERV-Spuma.61-Lca	WFAB01001028.1:44258-45250	WFAB01001028.1	44258-45250	502,597	993
ERV-Spuma.62-Lca	WFAB01001050.1:c458150-457155	WFAB01001050.1	c458150-457155	486,959	996
ERV-Spuma.63-Lca	WFAB01001063.1:c355452-354445	WFAB01001063.1	c355452-354445	525,379	1008
ERV-Spuma.64-Lca	WFAB01001066.1:110152-111159	WFAB01001066.1	110152-111159	628,656	1008
ERV-Spuma.65-Lca	WFAB01001066.1:431586-432719	WFAB01001066.1	431586-432719	628,656	1134
ERV-Spuma.66-Lca	WFAB01001067.1:67995-68996	WFAB01001067.1	67995-68996	656,105	1002
ERV-Spuma.67-Lca	WFAB01001067.1:324668-326002	WFAB01001067.1	324668-326002	656,105	1335
ERV-Spuma.68-Lca	WFAB01001067.1:c346617-345580	WFAB01001067.1	c346617-345580	656,105	1038
ERV-Spuma.69-Lca	WFAB01001133.1:34777-36039	WFAB01001133.1	34777-36039	419,834	1263
ERV-Spuma.70-Lca	WFAB01001133.1:c238862-237861	WFAB01001133.1	c238862-237861	419,834	1002
ERV-Spuma.71-Lca	WFAB01001139.1:345068-346183	WFAB01001139.1	345068-346183	413,620	1116
ERV-Spuma.72-Lca	WFAB01001198.1:69822-70814	WFAB01001198.1	69822-70814	324,590	993
ERV-Spuma.73-Lca	WFAB01001198.1:c253289-252300	WFAB01001198.1	c253289-252300	324,590	990
ERV-Spuma.74-Lca	WFAB01001206.1:208996-209988	WFAB01001206.1	208996-209988	321,622	993
ERV-Spuma.75-Lca	WFAB01001256.1:185368-186330	WFAB01001256.1	185368-186330	277,359	963
ERV-Spuma.76-Lca	WFAB01001298.1:21531-22523	WFAB01001298.1	21531-22523	127,317	993
ERV-Spuma.77-Lca	WFAB01001301.1:c136712-135666	WFAB01001301.1	c136712-135666	254,185	1047
ERV-Spuma.78-Lca	WFAB01001335.1:119728-120720	WFAB01001335.1	119728-120720	232,100	993
ERV-Spuma.79-Lca	WFAB01001382.1:c134333-133170	WFAB01001382.1	c134333-133170	149928	1164
ERV-Spuma.80-Lca	WFAB01001388.1:199883-201148	WFAB01001388.1	199883-201148	208,276	1266
ERV-Spuma.81-Lca	WFAB01001561.1:82449-83714	WFAB01001561.1	82449-83714	136,082	1266
ERV-Spuma.82-Lca	WFAB01001561.1:92091-93422	WFAB01001561.1	92091-93422	136,082	1332
ERV-Spuma.83-Lca	WFAB01001633.1:28084-29067	WFAB01001633.1	28084-29067	121,426	984
ERV-Spuma.84-Lca	WFAB01001689.1:c96960-95989	WFAB01001689.1	c96960-95989	101806	972
ERV-Spuma.85-Lca	WFAB01001777.1:c38718-37732	WFAB01001777.1	c38718-37732	91,449	987
ERV-Spuma.86-Lca	WFAB01001796.1:c36706-35699	WFAB01001796.1	c36706-35699	55,590	1008
ERV-Spuma.87-Lca	WFAB01001896.1:44979-46187	WFAB01001896.1	44979-46187	68258	1209
ERV-Spuma.88-Lca	WFAB01001979.1:c59191-58208	WFAB01001979.1	c59191-58208	67,713	984
ERV-Spuma.89-Lca	WFAB01001990.1:24917-26182	WFAB01001990.1	24917-26182	67,021	1266
ERV-Spuma.90-Lca	WFAB01001990.1:34505-35836	WFAB01001990.1	34505-35836	67,021	1332
ERV-Spuma.91-Lca	WFAB01002077.1:c12181-11198	WFAB01002077.1	c12181-11198	61,132	984
ERV-Spuma.92-Lca	WFAB01002758.1:15759-16736	WFAB01002758.1	15759-16736	27,321	978
ERV-Spuma.93-Lca	WFAB01002758.1:21880-22866	WFAB01002758.1	21880-22866	27,321	987
ERV-Spuma.94-Lca	WFAB01002778.1:4770-5747	WFAB01002778.1	4770-5747	27,026	978
ERV-Spuma.1-Lre	JADLOY010000230.1:28097-29089	JADLOY010000230.1	28097-29089	10051827	993
ERV-Spuma.2-Lre	JADLOY010000230.1:51098-52453	JADLOY010000230.1	51098-52453	10051827	1356
ERV-Spuma.3-Lre	JADLOY010000230.1:254721-256016	JADLOY010000230.1	254721-256016	10051827	1296
ERV-Spuma.4-Lre	JADLOY010000230.1:490462-492732	JADLOY010000230.1	490462-492732	10051827	2271
ERV-Spuma.5-Lre	JADLOY010000230.1:511285-512589	JADLOY010000230.1	511285-512589	10051827	1305
ERV-Spuma.6-Lre	JADLOY010000230.1:736856-738187	JADLOY010000230.1	736856-738187	10051827	1332
ERV-Spuma.7-Lre	JADLOY010000230.1:791760-793025	JADLOY010000230.1	791760-793025	10051827	1266
ERV-Spuma.8-Lre	JADLOY010000230.1:882490-883494	JADLOY010000230.1	882490-883494	10051827	1005
ERV-Spuma.9-Lre	JADLOY010000230.1:2400107-2401129	JADLOY010000230.1	2400107-2401129	10051827	1023
ERV-Spuma.10-Lre	JADLOY010000230.1:2667385-2668686	JADLOY010000230.1	2667385-2668686	10051827	1302
ERV-Spuma.11-Lre	JADLOY010000230.1:4172794-4174125	JADLOY010000230.1	4172794-4174125	10051827	1332
ERV-Spuma.12-Lre	JADLOY010000230.1:6441612-6442682	JADLOY010000230.1	6441612-6442682	10051827	1071
ERV-Spuma.13-Lre	JADLOY010000230.1:8896570-8898870	JADLOY010000230.1	8896570-8898870	10051827	2301
ERV-Spuma.14-Lre	JADLOY010000230.1:9335090-9336391	JADLOY010000230.1	9335090-9336391	10051827	1302
ERV-Spuma.15-Lre	JADLOY010000230.1:9684899-9686164	JADLOY010000230.1	9684899-9686164	10051827	1266
ERV-Spuma.16-Lre	JADLOY010000230.1:10048400-10050700	JADLOY010000230.1	10048400-10050700	10051827	2301
ERV-Spuma.17-Lre	JADLOY010000230.1:10286600-10287775	JADLOY010000230.1	10286600-10287775	10051827	1176
ERV-Spuma.18-Lre	JADLOY010000230.1:c9335-7980	JADLOY010000230.1	c9335-7980	10051827	1356
ERV-Spuma.19-Lre	JADLOY010000230.1:c554129-552792	JADLOY010000230.1	c554129-552792	10051827	1338
ERV-Spuma.20-Lre	JADLOY010000230.1:c801758-800517	JADLOY010000230.1	c801758-800517	10051827	1242
ERV-Spuma.21-Lre	JADLOY010000230.1:c1291060-1289759	JADLOY010000230.1	c1291060-1289759	10051827	1302
ERV-Spuma.22-Lre	JADLOY010000230.1:c1544802-1543471	JADLOY010000230.1	c1544802-1543471	10051827	1332
ERV-Spuma.23-Lre	JADLOY010000230.1:c2188919-2187588	JADLOY010000230.1	c2188919-2187588	10051827	1332
ERV-Spuma.24-Lre	JADLOY010000230.1:c2412201-2410870	JADLOY010000230.1	c2412201-2410870	10051827	1332
ERV-Spuma.25-Lre	JADLOY010000230.1:c5493818-5492565	JADLOY010000230.1	c5493818-5492565	10051827	1254
ERV-Spuma.26-Lre	JADLOY010000992.1:7049722-7051077	JADLOY010000992.1	7049722-7051077	9958788	1356
ERV-Spuma.27-Lre	JADLOY010000992.1:8049488-8050756	JADLOY010000992.1	8049488-8050756	9958788	1269
ERV-Spuma.28-Lre	JADLOY010000992.1:8077015-8078199	JADLOY010000992.1	8077015-8078199	9958788	1185
ERV-Spuma.29-Lre	JADLOY010000992.1:8189047-8190099	JADLOY010000992.1	8189047-8190099	9958788	1053
ERV-Spuma.30-Lre	JADLOY010000992.1:8415312-8416577	JADLOY010000992.1	8415312-8416577	9958788	1266
ERV-Spuma.31-Lre	JADLOY010000992.1:8815042-8816004	JADLOY010000992.1	8815042-8816004	9958788	963
ERV-Spuma.32-Lre	JADLOY010000992.1:8979344-8980306	JADLOY010000992.1	8979344-8980306	9958788	963
ERV-Spuma.33-Lre	JADLOY010000992.1:9534856-9536190	JADLOY010000992.1	9534856-9536190	9958788	1335
ERV-Spuma.34-Lre	JADLOY010000992.1:9770637-9771992	JADLOY010000992.1	9770637-9771992	9958788	1356
ERV-Spuma.35-Lre	JADLOY010000992.1:9954929-9957229	JADLOY010000992.1	9954929-9957229	9958788	2301
ERV-Spuma.36-Lre	JADLOY010000992.1:10263574-10264857	JADLOY010000992.1	10263574-10264857	9958788	1284
ERV-Spuma.37-Lre	JADLOY010000992.1:c1799134-1797797	JADLOY010000992.1	c1799134-1797797	9958788	1338

ERV-Spuma.38-Lre	JADLOY010000992.1:c3328990-3327635	JADLOY010000992.1	c3328990-3327635	9958788	1356
ERV-Spuma.39-Lre	JADLOY010000992.1:c4943990-4942725	JADLOY010000992.1	c4943990-4942725	9958788	1266
ERV-Spuma.40-Lre	JADLOY010000992.1:c6090825-6089557	JADLOY010000992.1	c6090825-6089557	9958788	1269
ERV-Spuma.41-Lre	JADLOY010000992.1:c6158816-6157485	JADLOY010000992.1	c6158816-6157485	9958788	1332
ERV-Spuma.42-Lre	JADLOY010000992.1:c7983380-7981413	JADLOY010000992.1	c7983380-7981413	9958788	1968
ERV-Spuma.43-Lre	JADLOY010000992.1:c8352646-8351480	JADLOY010000992.1	c8352646-8351480	9958788	1167
ERV-Spuma.44-Lre	JADLOY010000992.1:c8647052-8645799	JADLOY010000992.1	c8647052-8645799	9958788	1254
ERV-Spuma.45-Lre	JADLOY010000992.1:c8820270-8818939	JADLOY010000992.1	c8820270-8818939	9958788	1332
ERV-Spuma.46-Lre	JADLOY010000992.1:c8984572-8983241	JADLOY010000992.1	c8984572-8983241	9958788	1332
ERV-Spuma.47-Lre	JADLOY010000992.1:c9306035-9303735	JADLOY010000992.1	c9306035-9303735	9958788	2301
ERV-Spuma.48-Lre	JADLOY010000992.1:c9507550-9506285	JADLOY010000992.1	c9507550-9506285	9958788	1266
ERV-Spuma.49-Lre	JADLOY010000992.1:c9766940-9765954	JADLOY010000992.1	c9766940-9765954	9958788	987
ERV-Spuma.50-Lre	JADLOY010001211.1:c126520-125189	JADLOY010001211.1	c126520-125189	150,133	1332
ERV-Spuma.51-Lre	JADLOY010001538.1:675291-676559	JADLOY010001538.1	675291-676559	13568161	1269
ERV-Spuma.52-Lre	JADLOY010001538.1:2121931-2123199	JADLOY010001538.1	2121931-2123199	13568161	1269
ERV-Spuma.53-Lre	JADLOY010001538.1:2555891-2557246	JADLOY010001538.1	2555891-2557246	13568161	1356
ERV-Spuma.54-Lre	JADLOY010001538.1:3244755-3246086	JADLOY010001538.1	3244755-3246086	13568161	1332
ERV-Spuma.55-Lre	JADLOY010001538.1:5822633-5823964	JADLOY010001538.1	5822633-5823964	13568161	1332
ERV-Spuma.56-Lre	JADLOY010001538.1:6751506-6753806	JADLOY010001538.1	6751506-6753806	13568161	2301
ERV-Spuma.57-Lre	JADLOY010001538.1:7273364-7274695	JADLOY010001538.1	7273364-7274695	13568161	1332
ERV-Spuma.58-Lre	JADLOY010001538.1:9449758-9451113	JADLOY010001538.1	9449758-9451113	13568161	1356
ERV-Spuma.59-Lre	JADLOY010001538.1:10323558-10324823	JADLOY010001538.1	10323558-10324823	13568161	1266
ERV-Spuma.60-Lre	JADLOY010001538.1:10533204-10534469	JADLOY010001538.1	10533204-10534469	13568161	1266
ERV-Spuma.61-Lre	JADLOY010001538.1:10985181-10986476	JADLOY010001538.1	10985181-10986476	13568161	1296
ERV-Spuma.62-Lre	JADLOY010001538.1:10995739-10997034	JADLOY010001538.1	10995739-10997034	13568161	1296
ERV-Spuma.63-Lre	JADLOY010001538.1:11897159-11898541	JADLOY010001538.1	11897159-11898541	13568161	1383
ERV-Spuma.64-Lre	JADLOY010001538.1:11955073-11956377	JADLOY010001538.1	11955073-11956377	13568161	1305
ERV-Spuma.65-Lre	JADLOY010001538.1:12690860-12692110	JADLOY010001538.1	12690860-12692110	13568161	1251
ERV-Spuma.66-Lre	JADLOY010001538.1:12828310-12830262	JADLOY010001538.1	12828310-12830262	13568161	1953
ERV-Spuma.67-Lre	JADLOY010001538.1:13532322-13533677	JADLOY010001538.1	13532322-13533677	13568161	1356
ERV-Spuma.68-Lre	JADLOY010001538.1:13553385-13554740	JADLOY010001538.1	13553385-13554740	13568161	1356
ERV-Spuma.69-Lre	JADLOY010001538.1:c2638562-2637294	JADLOY010001538.1	c2638562-2637294	13568161	1269
ERV-Spuma.70-Lre	JADLOY010001538.1:c5021014-5019749	JADLOY010001538.1	c5021014-5019749	13568161	1266
ERV-Spuma.71-Lre	JADLOY010001538.1:c5159169-5157721	JADLOY010001538.1	c5159169-5157721	13568161	1449
ERV-Spuma.72-Lre	JADLOY010001538.1:c5863468-5862200	JADLOY010001538.1	c5863468-5862200	13568161	1269
ERV-Spuma.73-Lre	JADLOY010001538.1:c9223080-9221749	JADLOY010001538.1	c9223080-9221749	13568161	1332
ERV-Spuma.74-Lre	JADLOY010001538.1:c11484115-11481815	JADLOY010001538.1	c11484115-11481815	13568161	2301
ERV-Spuma.75-Lre	JADLOY010001538.1:c11778349-11777081	JADLOY010001538.1	c11778349-11777081	13568161	1269
ERV-Spuma.76-Lre	JADLOY010001538.1:c11939687-11937651	JADLOY010001538.1	c11939687-11937651	13568161	2037
ERV-Spuma.77-Lre	JADLOY010001538.1:c11967520-11966186	JADLOY010001538.1	c11967520-11966186	13568161	1335
ERV-Spuma.78-Lre	JADLOY010001538.1:c12450579-12449329	JADLOY010001538.1	c12450579-12449329	13568161	1251
ERV-Spuma.79-Lre	JADLOY010001538.1:c13309329-13308064	JADLOY010001538.1	c13309329-13308064	13568161	1266
ERV-Spuma.80-Lre	JADLOY010001538.1:c13565073-13563808	JADLOY010001538.1	c13565073-13563808	13568161	1266
ERV-Spuma.81-Lre	JADLOY010001574.1:43773-44756	JADLOY010001574.1	43773-44756	159,062	984
ERV-Spuma.82-Lre	JADLOY010001574.1:c104082-102727	JADLOY010001574.1	c104082-102727	159,062	1356
ERV-Spuma.83-Lre	JADLOY010001828.1:c229156-227801	JADLOY010001828.1	c229156-227801	383,266	1356
ERV-Spuma.1-Pma	AEFG01002868.1:c18205-17219	AEFG01002868.1	c18205-17219	42,435	987
ERV-Spuma.2-Pma	AEFG01005866.1:265-1266	AEFG01005866.1	265-1266	11,763	1002
ERV-Spuma.3-Pma	AEFG01008065.1:c1395-295	AEFG01008065.1	c1395-295	2,980	1101
ERV-Spuma.4-Pma	AEFG01011919.1:c3447-2464	AEFG01011919.1	c3447-2464	26,375	984
ERV-Spuma.5-Pma	AEFG01013105.1:c22513-21518	AEFG01013105.1	c22513-21518	31,127	996
ERV-Spuma.6-Pma	AEFG01017992.1:8942-9925	AEFG01017992.1	8942-9925	16,228	984
ERV-Spuma.7-Pma	AEFG01018298.1:c9606-8629	AEFG01018298.1	c9606-8629	20,411	978
ERV-Spuma.8-Pma	AEFG01021149.1:c14837-13848	AEFG01021149.1	c14837-13848	31,475	990
ERV-Spuma.9-Pma	AEFG01023326.1:c8927-7557	AEFG01023326.1	c8927-7557	7557	1371
ERV-Spuma.10-Pma	AEFG01026666.1:15785-16825	AEFG01026666.1	15785-16825	18275	1041
ERV-Spuma.11-Pma	AEFG01027239.1:16822-17796	AEFG01027239.1	16822-17796	18,144	975
ERV-Spuma.12-Pma	AEFG01031207.1:c4753-3764	AEFG01031207.1	c4753-3764	10,074	990
ERV-Spuma.13-Pma	AEFG01033864.1:21764-22759	AEFG01033864.1	21764-22759	23346	996
ERV-Spuma.14-Pma	AEFG01036928.1:c1207-227	AEFG01036928.1	c1207-227	5,088	981
ERV-Spuma.15-Pma	AEFG01039876.1:10140-11126	AEFG01039876.1	10140-11126	15,942	987
ERV-Spuma.16-Pma	AEFG01041665.1:3280-4281	AEFG01041665.1	3280-4281	14,166	1002
ERV-Spuma.17-Pma	AEFG01042895.1:c4617-3622	AEFG01042895.1	c4617-3622	5,312	996
ERV-Spuma.18-Pma	AEFG01044146.1:c4688-3693	AEFG01044146.1	c4688-3693	11,877	996
ERV-Spuma.19-Pma	AEFG01046600.1:8911-9948	AEFG01046600.1	8911-9948	9,963	1038
ERV-Spuma.20-Pma	AEFG01053972.1:950-1936	AEFG01053972.1	950-1936	6,152	987
ERV-Spuma.21-Pma	AEFG01059754.1:937-1923	AEFG01059754.1	937-1923	4,399	987
ERV-Spuma.22-Pma	AEFG01070560.1:c4317-3022	AEFG01070560.1	c4317-3022	3022	1296
ERV-Spuma.23-Pma	JAAIYE010000041.1:130429-131424	JAAIYE010000041.1	130429-131424	1,871,265	996
ERV-Spuma.24-Pma	JAAIYE010000041.1:1799138-1800442	JAAIYE010000041.1	1799138-1800442	1,871,265	1305
ERV-Spuma.25-Pma	JAAIYE010000041.1:c828291-827047	JAAIYE010000041.1	c828291-827047	1,871,265	1245
ERV-Spuma.26-Pma	JAAIYE010000688.1:142134-143432	JAAIYE010000688.1	142134-143432	15,679,795	1299
ERV-Spuma.27-Pma	JAAIYE010000688.1:12300570-12301544	JAAIYE010000688.1	12300570-12301544	15,679,795	975
ERV-Spuma.28-Pma	JAAIYE010000688.1:12741393-12742838	JAAIYE010000688.1	12741393-12742838	15,679,795	1446

ERV-Spuma.29-Pma	JAAIYE010000688.1:c13829939-13828953	JAAIYE010000688.1	c13829939-13828953	15,679,795	987
ERV-Spuma.30-Pma	JAAIYE010000695.1:10266075-10267400	JAAIYE010000695.1	10266075-10267400	34195180	1326
ERV-Spuma.31-Pma	JAAIYE010000695.1:14606258-14607232	JAAIYE010000695.1	14606258-14607232	34195180	975
ERV-Spuma.32-Pma	JAAIYE010000695.1:c2102920-2101592	JAAIYE010000695.1	c2102920-2101592	34195180	1329
ERV-Spuma.33-Pma	JAAIYE010000695.1:c2127947-2126619	JAAIYE010000695.1	c2127947-2126619	34195180	1329
ERV-Spuma.34-Pma	JAAIYE010000695.1:c4813371-4812064	JAAIYE010000695.1	c4813371-4812064	34195180	1308
ERV-Spuma.35-Pma	JAAIYE010000695.1:c4848614-4847652	JAAIYE010000695.1	c4848614-4847652	34195180	963
ERV-Spuma.36-Pma	JAAIYE010000695.1:c5776920-5775592	JAAIYE010000695.1	c5776920-5775592	34195180	1329
ERV-Spuma.37-Pma	JAAIYE010000695.1:c10722239-10720914	JAAIYE010000695.1	c10722239-10720914	34195180	1326
ERV-Spuma.38-Pma	JAAIYE010000704.1:c9412818-9411505	JAAIYE010000704.1	c9412818-9411505	13,533,735	1314
ERV-Spuma.39-Pma	JAAIYE010000716.1:118466-119761	JAAIYE010000716.1	118466-119761	120864	1296
ERV-Spuma.40-Pma	JAAIYE010000716.1:392952-394247	JAAIYE010000716.1	392952-394247	120864	1296
ERV-Spuma.41-Pma	JAAIYE010000732.1:12919157-12920230	JAAIYE010000732.1	12919157-12920230	12,997,950	1074
ERV-Spuma.42-Pma	JAAIYE010000732.1:12995354-12996331	JAAIYE010000732.1	12995354-12996331	12,997,950	978
ERV-Spuma.43-Pma	JAAIYE010000732.1:c4193728-4192649	JAAIYE010000732.1	c4193728-4192649	12,997,950	1080
ERV-Spuma.44-Pma	JAAIYE010000899.1:c120607-119621	JAAIYE010000899.1	c120607-119621	197,704	987
ERV-Spuma.45-Pma	JAAIYE010000899.1:c137018-136038	JAAIYE010000899.1	c137018-136038	197,704	981
ERV-Spuma.46-Pma	JAAIYE010000953.1:10956438-10957742	JAAIYE010000953.1	10956438-10957742	12,408,743	1305
ERV-Spuma.47-Pma	JAAIYE010000953.1:c8580234-8579233	JAAIYE010000953.1	c8580234-8579233	12,408,743	1002
ERV-Spuma.48-Pma	JAAIYE010001040.1:c108402-107416	JAAIYE010001040.1	c108402-107416	138,695	987
ERV-Spuma.49-Pma	JAAIYE010001062.1:98130-99110	JAAIYE010001062.1	98130-99110	127,909	981
ERV-Spuma.50-Pma	JAAIYE010001083.1:c131991-131011	JAAIYE010001083.1	c131991-131011	248,473	981
ERV-Spuma.51-Pma	JAAIYE010001411.1:889687-890673	JAAIYE010001411.1	889687-890673	4,109,867	987
ERV-Spuma.52-Pma	JAAIYE010001411.1:1420574-1421902	JAAIYE010001411.1	1420574-1421902	4,109,867	1329
ERV-Spuma.53-Pma	JAAIYE010001411.1:1845200-1846390	JAAIYE010001411.1	1845200-1846390	4,109,867	1191
ERV-Spuma.54-Pma	JAAIYE010001411.1:c1798405-1797215	JAAIYE010001411.1	c1798405-1797215	4,109,867	1191
ERV-Spuma.55-Pma	JAAIYE010001411.1:c1984117-1982927	JAAIYE010001411.1	c1984117-1982927	4,109,867	1191
ERV-Spuma.56-Pma	JAAIYE010001411.1:c2372613-2371573	JAAIYE010001411.1	c2372613-2371573	4,109,867	1041
ERV-Spuma.57-Pma	JAAIYE010001411.1:c3012132-3011137	JAAIYE010001411.1	c3012132-3011137	4,109,867	996
ERV-Spuma.58-Pma	JAAIYE010001411.1:c3664427-3663429	JAAIYE010001411.1	c3664427-3663429	4,109,867	999
ERV-Spuma.59-Pma	JAAIYF010000230.1:163442-164734	JAAIYF010000230.1	163442-164734	751,301	1293
ERV-Spuma.60-Pma	JAAIYF010000284.1:200997-202001	JAAIYF010000284.1	200997-202001	433,613	1005
ERV-Spuma.61-Pma	JAAIYF010000510.1:c931881-930907	JAAIYF010000510.1	c931881-930907	1,307,572	975
ERV-Spuma.62-Pma	JAAIYF010000593.1:60539-61519	JAAIYF010000593.1	60539-61519	61,912	981
ERV-Spuma.63-Pma	JAAIYF010000663.1:32481-33482	JAAIYF010000663.1	32481-33482	71,374	1002
ERV-Spuma.64-Pma	JAAIYF010000705.1:c393414-392428	JAAIYF010000705.1	c393414-392428	487,411	987
ERV-Spuma.65-Pma	JAAIYF010001153.1:335975-336955	JAAIYF010001153.1	335975-336955	398,201	981
ERV-Spuma.66-Pma	JAAIYF010001280.1:486502-487491	JAAIYF010001280.1	486502-487491	695,770	990
ERV-Spuma.67-Pma	JAAIYF010001280.1:c363582-362587	JAAIYF010001280.1	c363582-362587	695,770	996
ERV-Spuma.68-Pma	JAAIYF010001460.1:86790-87785	JAAIYF010001460.1	86790-87785	369,182	996
ERV-Spuma.69-Pma	JAAIYF010001689.1:23623-24591	JAAIYF010001689.1	23623-24591	152,170	969
ERV-Spuma.70-Pma	JAAIYF010002030.1:301613-302938	JAAIYF010002030.1	301613-302938	1,286,594	1326
ERV-Spuma.71-Pma	JAAIYF010002030.1:326615-327616	JAAIYF010002030.1	326615-327616	1,286,594	1002
ERV-Spuma.72-Pma	JAAIYF010002030.1:668597-670024	JAAIYF010002030.1	668597-670024	1,286,594	1428
ERV-Spuma.73-Pma	JAAIYF010002030.1:c827566-826322	JAAIYF010002030.1	c827566-826322	1,286,594	1245
ERV-Spuma.74-Pma	JAAIYF010002080.1:c296532-295567	JAAIYF010002080.1	c296532-295567	909,685	966
ERV-Spuma.75-Pma	JAAIYF010002132.1:c434130-433147	JAAIYF010002132.1	c434130-433147	540,935	984
ERV-Spuma.76-Pma	JAAIYF010002235.1:440232-441221	JAAIYF010002235.1	440232-441221	470,498	990
ERV-Spuma.77-Pma	JAAIYF010002245.1:337378-338364	JAAIYF010002245.1	337378-338364	458,286	987
ERV-Spuma.78-Pma	JAAIYF010002357.1:c97999-97028	JAAIYF010002357.1	c97999-97028	62485	972
ERV-Spuma.79-Pma	JAAIYF010002452.1:c72760-71765	JAAIYF010002452.1	c72760-71765	197,654	996
ERV-Spuma.80-Pma	JAAIYF010002495.1:c266062-264758	JAAIYF010002495.1	c266062-264758	269,465	1305
ERV-Spuma.81-Pma	JAAIYF010002511.1:153181-154167	JAAIYF010002511.1	153181-154167	265,619	987
ERV-Spuma.82-Pma	JAAIYF010002695.1:c110997-110020	JAAIYF010002695.1	c110997-110020	170,285	978
ERV-Spuma.83-Pma	JAAIYF010002855.1:34660-35964	JAAIYF010002855.1	34660-35964	159,505	1305
ERV-Spuma.84-Pma	JAAIYF010002855.1:c140125-139139	JAAIYF010002855.1	c140125-139139	159,505	987
ERV-Spuma.85-Pma	JAAIYF010003007.1:c6529-5552	JAAIYF010003007.1	c6529-5552	130,195	978
ERV-Spuma.86-Pma	JAAIYF010004000.1:c4809-3826	JAAIYF010004000.1	c4809-3826	11,620	984
ERV-Spuma.87-Pma	PIZI01000008.1:c10640197-10639217	PIZI01000008.1	c10640197-10639217	19,114,974	981
ERV-Spuma.88-Pma	PIZI01000008.1:c12427283-12426108	PIZI01000008.1	c12427283-12426108	19,114,974	1176
ERV-Spuma.89-Pma	PIZI01000014.1:c402698-401664	PIZI01000014.1	c402698-401664	16,331,332	1035
ERV-Spuma.90-Pma	PIZI01000014.1:c9925827-9924496	PIZI01000014.1	c9925827-9924496	16,331,332	1332
ERV-Spuma.91-Pma	PIZI01000017.1:c8568239-8567259	PIZI01000017.1	c8568239-8567259	15,686,774	981
ERV-Spuma.92-Pma	PIZI01000021.1:7466999-7468021	PIZI01000021.1	7466999-7468021	14677215	1023
ERV-Spuma.93-Pma	PIZI01000021.1:c8810956-8809649	PIZI01000021.1	c8810956-8809649	14677215	1308
ERV-Spuma.94-Pma	PIZI01000046.1:7383052-7384221	PIZI01000046.1	7383052-7384221	9,632,494	1170
ERV-Spuma.95-Pma	PIZI01004156.1:c9362-8319	PIZI01004156.1	c9362-8319	14,556	1044

Supplementary Table S4a. The genome annotation of Small spotted catshark and Jawless fish ERVs.

##gff-version 3								
##source-version geneious 11.1.2								
##sequence-region	ERV-Spuma.0-Lca	1	17673					
ERV-Spuma.0-Lca	Geneious	ORF	6888	10319	.	+	.	Name=ORF 2 (frame 3)
ERV-Spuma.0-Lca	Geneious	ORF	4540	6903	.	+	.	Name=ORF 1 (frame 1)
ERV-Spuma.0-Lca	Geneious	CDS	6888	10319	.	+	.	Name=pol CDS
ERV-Spuma.0-Lca	Geneious	CDS	10385	13190	.	+	.	Name=env CDS
ERV-Spuma.0-Lca	Geneious	CDS	4540	6903	.	+	.	Name=gag CDS
ERV-Spuma.0-Lca	Geneious	gene	6888	10319	.	+	.	Name=pol gene
ERV-Spuma.0-Lca	Geneious	gene	10385	13190	.	+	.	Name=env gene
ERV-Spuma.0-Lca	Geneious	gene	4540	6903	.	+	.	Name=gag gene
ERV-Spuma.0-Lca	Geneious	repeat_region	1	4201	.	+	.	Name=LTR
ERV-Spuma.0-Lca	Geneious	repeat_region	13488	17673	.	+	.	Name=LTR
##sequence-region	ERV-Spuma.0-Pma	1	16585					
ERV-Spuma.0-Pma	Geneious	ORF	4048	10821	.	+	.	Name=ORF 1 (frame 1)
ERV-Spuma.0-Pma	Geneious	ORF	10898	12622	.	+	.	Name=ORF 2 (frame 2)
ERV-Spuma.0-Pma	Geneious	CDS	6496	10821	.	+	.	Name=pol CDS
ERV-Spuma.0-Pma	Geneious	CDS	4048	6456	.	+	.	Name=gag CDS
ERV-Spuma.0-Pma	Geneious	CDS	10898	12622	.	+	.	Name=env CDS
ERV-Spuma.0-Pma	Geneious	gene	6496	10821	.	+	.	Name=pol gene
ERV-Spuma.0-Pma	Geneious	gene	4048	6456	.	+	.	Name=gag gene
ERV-Spuma.0-Pma	Geneious	gene	10898	12622	.	+	.	Name=env gene
ERV-Spuma.0-Pma	Geneious	repeat_region	12887	16581	.	+	.	Name=LTR
ERV-Spuma.0-Pma	Geneious	repeat_region	5	3705	.	+	.	Name=LTR
##sequence-region	ERV-Spuma.a-Etr	1	7160					
ERV-Spuma.a-Etr	Geneious	ORF	14	5671	.	+	.	Name=ORF 1 (frame 2)
ERV-Spuma.a-Etr	Geneious	CDS	1415	5677	.	+	.	Name=pol CDS
ERV-Spuma.a-Etr	Geneious	CDS	5754	7479	.	+	.	Name=env CDS
ERV-Spuma.a-Etr	Geneious	gene	14	1414	.	+	.	Name=gag gene
ERV-Spuma.a-Etr	Geneious	CDS	14	1414	.	+	.	Name=gag CDS
ERV-Spuma.a-Etr	Geneious	gene	1415	5677	.	+	.	Name=pol gene
ERV-Spuma.a-Etr	Geneious	gene	5754	7160	.	+	.	Name=env gene
##sequence-region	ERV-Spuma.a-Lre	1	6757					
ERV-Spuma.a-Lre	Geneious	CDS	566	4921	.	+	.	Name=pol CDS
ERV-Spuma.a-Lre	Geneious	CDS	4819	5853	.	+	.	Name=env CDS
ERV-Spuma.a-Lre	Geneious	ORF	566	4921	.	+	.	Name=ORF 1 (frame 2)
ERV-Spuma.a-Lre	Geneious	ORF	4819	5853	.	+	.	Name=ORF 2 (frame 1)
ERV-Spuma.a-Lre	Geneious	gene	566	4921	.	+	.	Name=pol gene
ERV-Spuma.a-Lre	Geneious	gene	4819	5853	.	+	.	Name=env gene
ERV-Spuma.a-Lre	Geneious	repeat_region	5	541	.	+	.	Name=LTR
ERV-Spuma.a-Lre	Geneious	repeat_region	6218	6753	.	+	.	Name=LTR
##sequence-region	ERV-Spuma.b-Lre	1	6570					
ERV-Spuma.b-Lre	Geneious	extracted region	1999	2006	.	+	.	Name=Extracted region from JADLOY010001804.1:7648910-7651917
ERV-Spuma.b-Lre	Geneious	CDS	1459	5793	.	+	.	Name=pol CDS
ERV-Spuma.b-Lre	Geneious	CDS	500	2074	.	+	.	Name=gag CDS
ERV-Spuma.b-Lre	Geneious	ORF	2017	5793	.	+	.	Name=ORF 2 (frame 1)
ERV-Spuma.b-Lre	Geneious	ORF	500	2074	.	+	.	Name=ORF 1 (frame 2)
ERV-Spuma.b-Lre	Geneious	gene	1459	5793	.	+	.	Name=pol gene
ERV-Spuma.b-Lre	Geneious	gene	500	2074	.	+	.	Name=gag gene
ERV-Spuma.b-Lre	Geneious	repeat_region	6084	6562	.	+	.	Name=LTR
ERV-Spuma.b-Lre	Geneious	repeat_region	5	481	.	+	.	Name=LTR
##sequence-region	ERV-Spuma.a-Lca	1	8145					
ERV-Spuma.a-Lca	Geneious	CDS	2606	5086	.	+	.	Name=gag CDS
ERV-Spuma.a-Lca	Geneious	CDS	5083	6483	.	+	.	Name=pol CDS
ERV-Spuma.a-Lca	Geneious	ORF	2606	5086	.	+	.	Name=ORF 1 (frame 2)
ERV-Spuma.a-Lca	Geneious	ORF	5083	6483	.	+	.	Name=ORF 2 (frame 1)
ERV-Spuma.a-Lca	Geneious	gene	2606	5086	.	+	.	Name=gag gene
ERV-Spuma.a-Lca	Geneious	gene	5083	6483	.	+	.	Name=pol gene
ERV-Spuma.a-Lca	Geneious	repeat_region	6714	8141	.	+	.	Name=LTR
ERV-Spuma.a-Lca	Geneious	repeat_region	37	1457	.	+	.	Name=LTR
##sequence-region	ERV-Epcilon.0-Ebu	1	9568					
ERV-Epcilon.0-Ebu	Geneious	motif	7568	8209	.	+	.	Name=evola env Conserved Domains
ERV-Epcilon.0-Ebu	Geneious	ORF	3650	6769	.	+	.	Name=ORF 2 (frame 2)
ERV-Epcilon.0-Ebu	Geneious	ORF	1176	2642	.	+	.	Name=ORF 1 (frame 3)
ERV-Epcilon.0-Ebu	Geneious	CDS	3650	6769	.	+	.	Name=pol CDS
ERV-Epcilon.0-Ebu	Geneious	CDS	6833	8536	.	+	.	Name=env CDS
ERV-Epcilon.0-Ebu	Geneious	CDS	1176	2642	.	+	.	Name=gag CDS
ERV-Epcilon.0-Ebu	Geneious	gene	3650	6769	.	+	.	Name=pol gene
ERV-Epcilon.0-Ebu	Geneious	gene	6833	8536	.	+	.	Name=env gene
ERV-Epcilon.0-Ebu	Geneious	gene	1176	2642	.	+	.	Name=gag gene

ERV-Epcilon.0-Ebu	Geneious	repeat_region	5	567	.	+	.	Name=LTR
ERV-Epcilon.0-Ebu	Geneious	repeat_region	8994	9564	.	+	.	Name=LTR
##sequence-region	ERV-Spuma.a-Ebu	1	4431					
ERV-Spuma.a-Ebu	Geneious	ORF	1328	4027	.	+	.	Name=ORF 1 (frame 2)
ERV-Spuma.a-Ebu	Geneious	gene	1328	4027	.	+	.	Name=pol gene
ERV-Spuma.a-Ebu	Geneious	CDS	1328	4027	.	+	.	Name=pol CDS
ERV-Spuma.a-Ebu	Geneious	CDS	163	1335	.	+	.	Name=gag CDS
ERV-Spuma.a-Ebu	Geneious	repeat_region	6	148	.	+	.	Name=LTR
ERV-Spuma.a-Ebu	Geneious	repeat_region	4286	4426	.	+	.	Name=LTR
##sequence-region	ERV-Spuma.0-Lre	1	18053					
ERV-Spuma.0-Lre	Geneious	repeat_region	1	4339	.	+	.	Name=LTR
ERV-Spuma.0-Lre	Geneious	repeat_region	13715	18053	.	+	.	Name=LTR
ERV-Spuma.0-Lre	Geneious	CDS	7339	11601	.	+	.	Name=pol CDS
ERV-Spuma.0-Lre	Geneious	CDS	5075	7264	.	+	.	Name=gag CDS
ERV-Spuma.0-Lre	Geneious	CDS	11677	13401	.	+	.	Name=env CDS
ERV-Spuma.0-Lre	Geneious	ORF	7339	11601	.	+	.	Name=ORF 2 (frame 2)
ERV-Spuma.0-Lre	Geneious	ORF	5075	7264	.	+	.	Name=ORF 1 (frame 3)
ERV-Spuma.0-Lre	Geneious	ORF	11677	13401	.	+	.	Name=ORF 3 (frame 2)
ERV-Spuma.0-Lre	Geneious	gene	7339	11601	.	+	.	Name=gag gene
ERV-Spuma.0-Lre	Geneious	gene	5075	7264	.	+	.	Name=gag gene
ERV-Spuma.0-Lre	Geneious	gene	11677	13401	.	+	.	Name=gag gene
##sequence-region	ERV-Spuma.0-Sca	1	9220					
ERV-Spuma.0-Sca	Geneious	BLAST Hit	4112	6550	.	+	.	
ERV-Spuma.0-Sca	Geneious	ORF	3920	6865	.	+	.	Name=ORF 2 (frame 3)
ERV-Spuma.0-Sca	Geneious	ORF	1538	3796	.	+	.	Name=ORF 1 (frame 3)
ERV-Spuma.0-Sca	Geneious	repeat_region	8352	9220	.	+	.	Name=LTR
ERV-Spuma.0-Sca	Geneious	repeat_region	1	866	.	+	.	Name=LTR
ERV-Spuma.0-Sca	Geneious	CDS	3920	6865	.	+	.	Name=pol CDS
ERV-Spuma.0-Sca	Geneious	CDS	1538	3796	.	+	.	Name=gag CDS
ERV-Spuma.0-Sca	Geneious	CDS	6989	8314	.	+	.	Name=env CDS
ERV-Spuma.0-Sca	Geneious	gene	3920	6865	.	+	.	Name=pol gene
ERV-Spuma.0-Sca	Geneious	gene	1538	3796	.	+	.	Name=gag gene
ERV-Spuma.0-Sca	Geneious	gene	6989	8314	.	+	.	Name=env gene

##FASTA

>ERV-Spuma.0-Lca

CTAACCCTTCTTCCATAATTGAGATTAATAA
GATTTTTATATATTATCTTTTTACTTGTTTTT
TGAAACACACCAAGCGCAGCAAACTGCAAA
TTGTTAAGTAATGAACCGGAGGAGGGCTAG
TTTACAACCTTGCTTCAAGGGAAGATAAGG
GGCACATGTCACCCGTGCAGGCGGCAGAG
TGTCAGCCAGACATAGTTAAGGCAAAAGCC
GCATGGTTTTAAAGTAACGTCAAAATTCGA
CAGAGTAGGCTAAATTATCGATCCTCGCGC
TGGAGACCAGGCTGCGAGCTACATCAATGA
ATTTGCTAATCCAATACTGAATGTTAATATT
AAAACGAATTCTATGACCTCAGCACATGGG
GGCAGTGTCGACTCAAAGGCAATGGAAGAA
CCTTTGTCTAAATCACAACCACAGAAAGCC
ACTGATTGTGTGTTCAGGGATTCCGAGCTA
GTGCACAGGGGGGGAGCTGATGTAAACAAC
TGGATGTGAGGGGGCCTTGGACCTGGAATC
CTCCCAGAGCAGAAGGTTGGACAGGGGGAG
AAAGAGTGTTGTATATCTCTCAAGGCGGCA
AATAACAAGGCTGCGGGGGTCACCAGAAGT
CTAGTTAAGACAAAAAGCTCAATTATTCTG
AAAGTAACGTTAAAGTCGGAATTTAACAAC
AAAGTTATTGATATTCACACCCACTCCAAT
CACAAGTTACGCCTACTGAACCATCACCAT
ATATGAAATGTTATTTCCATACTTTAATACT
ACTCGACCCGAAGCATATGACAATCAGAAC
CAAAATTGAATCGCACCCAGACAGGAGCGA
TAAGGTTGACGAGGGAAGGAAATTAAGAGC
GCAAACGTAAGCAAAAAGCCGGGAACAACA
AACAGCGAGTGGGGCTTCGACAGGCCATAA
TAACTCCCGGGGTCAGGGGACACAAGAAGA
CAAAAACCAGAACTGACTCCAAGGTTCAAC
TACTCAGGAGTGACGGAAATTAATGAGGCC
GGCAGGCAACAACAACGGCGAGCAGAGATA
AAGACTGCGGGCTATGAAATGCAGCTTCGT
CTCTGACAAACAAGTTAAGTTAAAGACATCA

ATATCGCACATAACATCAATGTTAACGCTAT
ACTATATTCCGCTTATATAACCATGCAGGCA
ACGAATTCCTAGGATCAAGAAACTTACCCCT
ATATCGGAAATAAATTAATAGTAATAAGTAT
AAGACAAAAGTAAAACTTATGGAAAAGCCAG
ATGAATCACCACCAGGTCGAATATAATACAT
ACGATGTTAAACCCAGGGTCGGAGAGAAAGG
GCCGTCTTTGTATCCCCGCAGCGCGGATAC
TACGCTCGGGNAAACACCTCCACTGGAGTCA
ATGGGAAACGACAGACCTTAGGTTACATACA
CTGTGAAATAGTAAGGGACAAATGTTAACTT
TAACCATTTCTTTGTGCGCCGGGTCGGAGAC
CCTCCCTCCAGGTTGTGCAACACGAACAAAC
AGACGATTTGTAACGTCTGTGTTTTGGGTTC
GCCAGACAAGAGATAAATTGCACCCAGACAG
CTTCGAGGCAATTTGTAACATCTGATTGTTTC
TTTATGGAGGGAGTGTGACAACGGAATATAG
TGCACACAAGGACATACGGACGTACGGACAG
CACACCTGCCTACGGACAGATCCTGACACAC
GAAGCCCCGCCTTGTGGGCGGGATCCAGGGG
CATAAAGGAAAGGATCCTGAGCATTGGAAAG
CAGAGGGTCCACTCAGGAGTGCGACCAGAGG
GGCGAGGGCGACCAGCGGGTCGACCCAGGAG
CCGGGAGGGTCGACTCAGGAGCGAGCTGCGA
CGGCTCGACTCATCTTCAGCTCGTCCGACAC
TGCGACCAGCGGGCCGACCCAGGAGCGCGGG
GAGCGGCTCCGCATCGACTCAGCAGGCGGGG
GGGTCAGCTCGACTCATCTTCAGCTCGTCAA
AGTCGGAGGTCTCTGCTGCCGCCGGAGTGGT
GGTTTGGCGACGATCCAGCGCTGCACGCTAC
GTGGGCGCCACCTACCGACCGGGTTCCGAAC
GTATCAACAGCCATCCGAACCATTCACCACC
GAGCGACTGATACGTATGACGCGCACCGCCC
ACGACCACCAGGCGTCTGTTTGACATAAAGA
GAACACTGACTATTCAAGACAACTCTTACCA
ACTCGAACCCATAAGCCATCCGTGGCTGGGA
AACGAGACCGAGTGATGGCCATCCGTCGTTT
ATTCGTAATTTTTATCTTTAATATTTAGTGGC
TAATCGCATAAATTCCCAACAGATCCATCAA
ATTCTTGGCACCTCCTTCCGCCGAGCCACGC
GAGTGGTGGCCCACCGGATCCTTATCAGGTC
CCAATTAGTAAACTTGGTATAAGATAACGAT
AGGAGCACCGTCGCAGGCCCTTACTCCGAGG
GTCAGATAACCTGCCGATGTATCTGTTGAGA
TTAAGAATAATAACTGTTTTTGATTGTGTTT
CGTATAATCTTTCAAGTATCTTTCAGTCATA
CGTACTCGCATCCTCTACAGAATATTCAACT
CCCAAGGTGTTAACAACGTGATAAGGGTTGC
ATAAGGAGTGCGGTACTGCGTGAACAATATT
TCATTCATTAACGTTAAGGTTTTCTTAGCATA
TGTTTTAACGACTCTCTCTCTCACTAACAAC
ATGCGGGATCCATGTGCCGTTGTCAAAGTGA
AATCGTTGATGATACTGTATTGATGCCGTGTT
GGATTGAGAGTTCGCATACTATATCATAGTT
TTAGTAATAAATTGATCAATACAGAAGTGGT
CTTTCCTGAAATCTATGCATAAGATCTGCTT
GATTTGAACTTGGTGACCTTAATAAACACAC
CTCAGTAAATGTTAAAGCGATAACGGGGTGA
CTAAAATATAACCACGAATACACACTCTGGC
GCGAGAATGAGACGAACCTAGACAACGATGG
CTAAGTTAATCTTGCATACTTACACCACACC
GCATACGATTAATTGCCTTATTCCGATCGAT
TCGTCACCGAGGCGGGGTCTGGTTATCATAA
TTATCATAATTACACCCACTCGCATAATTAA
CTCTCGAATTGGCATAATTAAAATGTGCGTA
ATTAACATATTTCCGGATAATTACACATTTC
TCATTTAATTATTATTATTTTCACTGAATATT
CATATACAACATAATAATTTCACCCACGCAC
ATAACAAATTTTCATAACCAACATATACAAC
GGCGTCGTCCGGGATCTCTCGCAGACAGTGT
ATGAAGGAATGAATCACTGTTGTGTTAACCA

GATCGAACATTGCATTGTGATTGTAAACGGC
ACAAAACGCCCTCTGAGGTTAAAAACCAGAA
AAAACAAAGACAGCTGGGCTGAGATTATTTC
AACTGACGCATCCTGTGATACAATTGTTTAT
GAAAGGGAATACTAGAGTAACTCATGCCACA
CTAGGATTAAGATACACTCTTAAGTCTATTA
CATAATAGAGATGGCACAGGATCCACAACAT
GACACACCAACGCATAACACTGATACAGAGT
AGGAGGAGATGGAATCTATGGAAAAACAATA
TGTGTTAAAACGTACTGGACGGACTGCTAGC
GAGCAGAGAGATATATGGGAGGGGAGCCATA
TTAAGGCCCATAGAAGGAAACTACTTAAGGA
ATGTGGCAAAGCCAAACGTGACGTAAAAATT
GCGAGGGTAGACTCGTATCTCCTCGCACAGA
AAAGATTATGGAATGAGGAAAAATTTGAAAC
AGAGAAACTGTCCTTAATGGTGCAGGTAGAC
GCCCTCGTGAGAGAGAAAGAATCCGCACTAA
TGAAACACCCAGACGTTGATACACAGAACAC
TAACAATTTACCACACTCCGAGCCACGTGGA
GGGCCAGATCCCACAATGAGTATGGCTACTA
ATTTTGATAAAGTAACACCCTCGCAAATCGA
AGAGGAAATAATAATACAAACGGGAAAACA
CACCCCAACGTTAGAGAGAGACAAATAATTA
CATCAGGATACACTGTAATGATAGAGCGGAA
GCTGGTGCAAGACGGGAAACATGACATAAAC
GGCAGACGCACGCTACGCAAGTATGACCGAC
ATGACAGAGACAGCTCACCAGAGAAACGCAC
ACACGACCAACACGACACACGTAACAGGAGC
AGACGCACATCACACAAGCATGAGCCATGGC
AATCACCGAGCCAAGTTGACCCACGTAACAC
GCACTACAAAAGTTATGTTAAGCAGAGCCAC
GACGGCTCATCTGACACTGACTCCTCTGACA
ACGACATGGGTACCAGAGAGAGCTCACCAGA
GACATGTAAAAATCACCCACGCAAAACCACC
AATCACAGAGACAGGAATCCCGATAACCATC
ACAAACATAAATTCAACACCTCACCTAGCAC
ACGTAGTAGGCAGCATGAAGGTCGAGGACAA
CCACATAGACACCGCTCAAGGAGATCACGCA
ACAAGCACGAATCACAAGACCCACATAAGGA
ACACAAGCAACGGGACAGATACGAATCCTCC
AGATCACGACACAGACACGGGACACATGACA
GTGGAGACAAGGGTAGCTCAAGGGAGCACTC
CGACACCACTGATGACGAACAACACACTTTT
ACACTAAAACTAATGGACAAAGCACTCGTGA
ATTTCGATCCACATAACAAGAACAGGGATAT
CATAGCTACAGTAGAACAGGAGGCTAAGATC
GGTAAATCAGCAGAAAAGGCATGTTTATTAT
TACATGAACCCGCCAGACCATGGGTAGCCTC
TAGGGTGAAGAGTGACTTTAAACAATTGAGC
GTTAAGAGATTTGGGGAATACTCCACACTAT
TGAACGCGGCAAAGTTGATGACCCAGCGTGT
CCCACGAGATTATCTCCTACGCCTTAAGAAA
GCAGGGGACGTGGACAATGCCCAGGGAGATA
TAAAAGTGATGTTCTTCCAATCATTGAGACC
GAGAATAGTAGGAATGATGTTGGACCCCGAC
CTACGCCAGATTGAAAGCAAGGCCCACACGC
ACGGACGGAAAACCACTGGCATAGCCAAAAC
CACCAGAGATAAAGAATGCCTCGATCTGGAC
TCTCCAAGGAATAATATTAGCGCCACACGGC
AATTCCCCCAGGAGAGGGCGCCTAACCCAAT
ACCCGTCCACAACACGCTGAATAACGTTCCA
GGAACGAGGCCAGAGGAACTTGGCACCCACA
ACCATGGAATGCAGAAGAGGGCGGAGGCTGC
GGAGGAAGAGAGAGTGACGGGCCATACCTAC
GCCGACGCCCACCAAACCAGTCACCATATCC
GCCGCCTCCACTAGCAATAGAGCATAGACCA
AACAGGAATCGATATGCTCACCACATTAGGA
AGCTCCTAGGGAGTATGAAGACTCCCATGCA
GGGAAGAATACCAACTATACCCACACAGGAC
CCCAGAGCAAAGGTAGCCCAAGTGAACGTAC
GAAGCCTCGAAGCAATCACCGAAGTGGTACC

AGGGTACCATCTGGGGGAATTGATTCCCGTA
GGACGAGTCCACGTGGAAGTAATACTGCAAC
CCAGCCAGGGCCTTTGTGATACTGGAGCAGA
GATATCCAGAGAATTATTTGACGAACTCCAC
CAGAGGAAGAATGAAACACTACAAATGATGC
TTACCTACTCAGGATGTGAAGGGACCCACAT
GGGCAAAGTTATGACGAAGTTGACGTTCAAC
ATGAACCATCCCCTATATGTCGTACAATACC
AACTGATTATTGGGATCGAACTACTGAATCC
CATTATGAACCTTGAATCCCGACAAATTTGC
ATGCCGGAGCAACAAAGGGACTCAGAAGATC
TGAGAGACTCCACGGGAATCCAAGTGATAAC
CGACGACCTATCAACGACAATCAGGAACGA
CCAGCAGGAATTCGAATCACTCAGCTAATGA
TGAGAGAAGACCGAAAGGAAGCCGACTTAGA
ACCGAATGACCGTCCCCTCATGGAGTATATA
GGAGGCCAAACCGACGAACTGCAAACTGATC
ACAAGGGCAAGAGCGTATTACCCTCGGATGA
AACTCAACCGATAGTAACGCAAAAGGAACTT
CCGCCCGAAACCACGAAGGCCCTAGATCCAA
AAAGAGTCCTTACAATAGAAGAACAAATAGA
TGAAGCAGCAGACGCCCTAGAGAGCGACACT
GAGCTACGACAACTGCTCCAAAAATACAACT
CCAAAGACTCCTATGATTGTGGCAAAACCGA
GGTATCCATTCCCACACTGGAGGGAGCACCA
GTGAAACAATACAAGATTCCACTGGCCGCCT
TCCAAGAAACCCTAGAGAACCTACTCAAAAA
CCGGGAATGCAACTCGACCTACAACTCACCC
GTACTAAAACCCACGGGAAAATGGAGACTCA
ACCGACAACTCAATAAAACAGTTCCCCTATC
AATGGCAGAAATCGACCACGGTCTCAACCAA
GCAAAGGTACTCACCACAATGGACCTAGCAA
GGACCATGCCAGTAAAAGAAACCGATCAATA
CTTCACCTTCGACGGAATACAGTATACTTGC
CCGTTCGGATACTCGAACTCACCTGCAGACT
TCCTCCACAAGGCGATGGGCGACGCAAAAGA
CATCGTCTACGTCGACGACATACTAGTCAAC
TGGGAAGAACACCTCGAGAGCCTCCAGCATA
AGTTAAAGGAAGCAGGCGCAAAGATTTCAAT
ACAGTGGGCACGCAAAAGGGTGGACTACCTC
GTCGGCACTGAGGGAATGTTACCCCAAACCA
AAGCCCTCCTCGCACTCAAATCGCCCACCAC
CCTTCGGAGCTTCTTAGGGATTTGCAACTAC
TTCGTGGACGACTACGCAGGAATCGTTCGAC
AACTACTGCAAAAAGACGAACCATGGGAGTC
ACAGGAAGCCGCAGAAAAAGAATTAAAACGA
GAGGCATCCTGCCTAGCATACCCAGAGAAAC
ACTACCTGGAGACCGCATACTCAGATCATAC
GGTACTGTACCAGAGACAGGAAGCAGAGAAA
GCCTATGCCAGCAAAGCCCTCCGGGGAGTGC
TCTCCGAGTGTGAGAAGGCGATATTTGGAAA
CATACAACATTTTCGGAACTTGTTGAACGGA
ATCCTGGAAACGAATCATGAATCCTTGGCAT
GTAAGAAGATTCGAGAGGGCCGCGTAACCAC
AGCCAGCTGGGCACTGACACTCCAAGGCCTA
GTGAAATACGCAAAAAACAAGAAAAGTCCAA
GGTCTTGCCGATTTACATGATTGTACCTTTGA
AGCAGAGGACGAAAAGATACCTCCGGAGGAC
CTGCCCTTCAGGAAGGAGACATGTGCCGACC
TATACATCGATGGATGCTCCAAGATGAAGGA
TCACGCTGGGTCAGGAGTCATTTGGGAAACT
GCAGGGATTCAAGAGGGATTTCAGCTGGGAC
ACCAGTATGCAGAGTTGGTCGGGGTACACAT
GATGGCATCCGATAACCAAATACGGACCATC
ACTGATTCCAACTACGTACAGCACAGCTTCC
TACCCATATGGAAAAAAAACGAGATGAAGAA
TAAACCAATTCACCATCGAGAACTATTCGAC
AAGATGGTTCAGAACAAGGATATGAAGATAT
AAGTCAAAGGGCACTCCAAGATACCTGGTCA
TGGGAACGACCAAGCAGATGCCATAGCAAAA
CTGGAAGGCACCCCGTGGAAGCTAGAAGACC

GAGACGGACAACAAGAGTCGACGAACCAAG
GATAAGACTGGTCACAAGAAGTGGGAAGAA
GCTGGAACCAGAGCCCAATTGAACAGTGCC
AAGACCTGATCAGAATGCAACAAGAGGACG
GACACTCGCCGATCATATCCAAGATCCAGC
ATCACTACTGACAACTTGAATGACAACAAA
TCATGCATAGCCAAATCAAAAGGTTCTCAA
GCTACTGGTATACACAAACGAAAAAGACGG
TGGGTAGTCCCTCTCAATATCGAGAAATAA
CGCCCACAATGAACCGTGTAGCGGCCACCG
AATACGGGACAGATGCTGCGACAGGTGGCC
AAATGGGGAAGACATACACAAACATGTGTC
GTGTGTTGTAAGTACCAGCCAACGACCTCA
CACCCCTACAGAGGAGGGGAGCAGATTATC
CCTACAAATCGACTGGATAGGACCTGTCAA
AAAGGGAACAAGTACATGCTAACTGTGACC
CTAAATGGACAGAGTGTTTACCAGCACCCA
GGAAACAACCGCTATACTACTGATTAACCA
AGATGGGGCCTACCGTCAACGATCGATTCA
GCCACTTCACAGCCGAGGTCATCACAGAAG
GTTGGGAGTAAAAAGACAATTACATGTCGC
CAATCCTCAGGACAGGTGGAAAGAGCCAAC
TGAGCCTCCTAAAGAAATATGTGTCTACCA
CTGGGATACGAAACTACCCCTAGTCTTGAT
GCCACACCCAACCGAGCTACAGGCAGGACA
TCATGACAGGGAGGCTAATGACCCTCCCAG
ATATGTCCNAGCGGAGGACGAGACCCCGAC
CCAACTGAATACCTGACGGGACTCAATAGT
GCACATTCGCTATGGTCAGGGACCAGTTGC
TCAGGGAGCAAAGCATACTATGACCAAAAA
CAGAATACCAGCTAGGAGACCAGGTGTGGT
CGAGAAAAGGAAACAGAAGTGCAGGAAATT
TGGACAGGACCACACGAGGTTGTTGACAAA
TCGTATATCAGCTAAAAATCACAAAGACAG
GCAATACAAGTGGGTGCACATCAACCAAAT
CCTGTGCTGGTGGAGCGAGATGAGGAATAA
GGGGGGGGAACTAAACTAATGGGCTTATCA
ATATGTAACATGTTTTCCAGGGAATGGCAG
CACGCTCCTGCTAACGTGCCTTGCCAAGCTA
CAAGACCTGATCAAGCCAGGACCAGAATCC
TGAGAGACTCCTCCAGCTTCCTAGTAACCG
AGTAACCCAACAAGTCTATGTCTCCTTGGA
GTGGTAAAGAAGCACTTCGGGAAGTTACCA
CCCAAGCAACCCGAATATGGTATTATGACT
TAGTGAACAGAATATCGGAACGATCCTGAA
AGAACCATGATACTTCCCCCACCACTGTCA
TTCCAAGAGGTCTATCGGCCTGGGAGGGTC
TATCCGGTTTTAGCTCCTTAATCACACTAG
ATCCAACGCCGTAAAGATTGGACACCTCAC
GAGAATTTACAGCTCGATATGCATCAGATC
TACAACACCAACAAAAGAATCTGGTCGAAA
CTCCGAGACACCGTGGTTCTCGTAAACCTA
TGATTAACTTAACATTCCAAGAATTGAGTT
AGRTCTATACGAAGAAGAGAGTATTCGAGA
TGGGTGATCCAAGACTTGTTGCGTAGCATC
TCAGTGATCTTATACAAGGACAAATTCCGT
AACTCCAACACTGATAAAAGATGTGCTACA
CGGAAACAATCAGTCCAACTCAAGTACAAA
CATGGGTACAGCCACACCCATACATGTGGA
AGACAGCTGGCGTTTTTGCTAAATTTACCC
CCGGAAATGTATTCCGATTGCGGACAGTAT
TACATGGAATAACGACATTTACTCCAAGAT
TTAGTGGTGGCCTACCAGGAAGAAGGCCCC
GGTGCCTGACCTAAACCTGTGTCAAATAAT
ATTGGCTCTGCCCAGGGAACCATTTCTGAC
GAAATAATGTGTGGTCTGTCAAACCTAAGC
CTTGCAAGTTTAAGCTAAGTAAACCCAAAG
AACAGTGGCCATGATAGCTAATGCCCAGTG
ACCCCTCTAGACACGGCAATTGTTTCCACC
CCATAACCATGGAAGTTCCGATTCCAAGCC
GGTCACAATACCAAGAGGAACATTCGTACG

AAGGTATTATATAACCTAGACAAAAGCCTAC
AGGTAGAAATAGTAGACACATTCCGAGGCCA
GATAAGCAGCACTCTGTCCAACTTGTTGAAC
TTCCATATGGTGAAATTATCCCTCAGCGAAT
ACATAACAGATCTTACTTGGGCGACCGAAGA
TCAGCGAAATCAACAGATGGGGATGGGAATC
ACAGGGATGATCATACTAGTCCTGGCTATTC
CATACCATGTGGCCAAAATGAATGCCGAACT
AATCGCAGGCACAAGCCACATACCATTCGAC
AGGGATAGAGCCAAGGCCTGATGATGCCACC
CCGTAGAGACTATCTTTTTAACGATATCTCC
TAGTCAATAGAATAATTTTCCCTCCCTTATC
TTCCTTTAGAATTTTTTTTTTTTTTTTTCTT
CAAATATAACCATGTTAAGTTGTGACACGTA
TGTAAGGAATAGCAGCAAAACTTGAGGACAA
CATGATGGAAGAAGGGTATACATTTTTTTAA
TGGCCATTTGACCACTGGTCATTTTTAAAAC
GTTGTATCCTAACCCTTCTTCCATAATTGAGA
ATTTGATGATTTTTATATATTATCTTTTTACT
ATGAGTGAAACACACCAAAGCGCAGCAAAC
CCAGGTGTTGTTAAGTAATGAACCGGAGGAC
GGCAGAATTTGCAACCTTGCTTCAAGGCAAC
GATGAACGGGCACATGTCACCCGTGCAGGCC
GTGGGAGTGTCAGCCAGACATAGTTAAGGCA
TCATCCTGCATGGTTTTAAAGTAACGTCAAA
TCAACAACAGAGTAGGCTAAATTATCGATCC
CTCAAATTGGAGACCAGGCTGCGAGCTACAT
ATTGCCCATTTGCTAATCCAATACTGAATGT
TGAAGTCAAAACGAATTCTATGACCTCAGCA
ATAATAGGGCAGTGTCGACTCAAAGGCAATC
AGAGGCGCCTTTGTCTAAATCACAACCACAC
GAATCATCACTGATTGTGTGTTCAGGAATTCC
AGAACACGTGCACAGGGGGGAGCTGATGTAA
CGCGTCTGGATGTGAGGGGGCCTTGGACCTC
ATAACACTCCCAGAGCAGAAGGTTGGACAGC
ATTTAAAAAGAGGGTTGTATATCTCTCAAGC
CAATTGAATAACAAGGCTGCGGGGTCACCAC
TTCGCCTAGTTAAGACAAAAAGCTCAATTAT
CTATTAAAGTAACGTTAAAGTCGGAATTTAA
AGGCTAAAGTTATTGATATTCACACCCACTC
CAGGCCACAAGTTACGCCTACTGAACCATCA
AGTCAATATGAAATGTTATTTCCATACTTTA
ATACAACTCGACCCGAAGCATATGACAATCA
TAAGTCAAAATTGAATCGCACCCAGACAGGA
CGATATAAGGTTGACGAGGGAAGGAAATTAA
GAACTGCAAACGTAAGCAAAAAGCCGGGAAC
CAGCAACAGCGAGTGGGGCTTCGACAGGCCA
ATAATAACTCCCGGGTCAGGGGACACAAGAA
AGACAAAAACCAGAACTGACTCCAAGGTTCA
ACCTACTCAGGAGTGACGGAAATTAATGAGC
ACGGCAGGCAACAACAACGGCGAGCAGAGA
GCAAAGACTGCGGGCTATGAAATACAGCTT
AAGCTCTGACAAACAAGTTAAGTTAAAGACA
ATAATATCGCACATAACATCAATGTTAACGC
TAAACTATATTCCGCTTATATAACCATGCAC
AATACGAATTCCTAGGATCAAGAAACTTACC
AGATATCGGAAATAAATTTATAGTAATAAGT
AAAAGACAAAAGTAAAACTTATGGAAAAGCC
GGATGAATCACCACCAGGTCGAATATAATAC
AACGATGTTAAACCCAGGGTCGGAGAGAAAC
GGCCGTCTTTGTATCCCCGCAGCGCGGGATA
ATACGCTCGGGAAACACCTCCACTGGAGTCA
ATGGGAAACGACAGACCTTAGGTTACATACA
CTGTGAAATAGTAAGGGACAAATGTTAACTT
AACCATTTCTTTGTGCGCCGGGTCGGAGAGA
CTCCCTCCAGGTGGTGCAACACGAACAAAGA
GACGATTTGTAACGTCTGTGTTTTGGGTTCA
CCAGACAAGAGATAAATTGCACCCAGACAGA
TCGAGGCAATTTGTAACATCTGATTGTTTGC
TATGGAGGGAGTGTGACAACGGAATATACCC
CACACAAGGACATACGGACGTACGGACACAC

CACCTGCCTACGGACAGATCCTGACACAGCA
AGCCCCGCCTTGTGGGCGGGATCCAGGGGAT
TAAAGTAAAGGATCCTGAGCATTGGAAACCA
GCGGGTCCACTCAGGAGTGCGACCAGAGGTC
GCGAGGGCGACCAGCGGGTCGACCCAGGAG
CGGAGGGTCGACTCAGGAGCGAGCTGCGAC
GCTCGACTCATCTTCAGCTCGTCGACCACAC
AGGACCGAGGGCGACAGCGGGCGACCCAGGA
CCGGGAGGGTCGACTCAGGAGCGAGCTGGA
GGCTCGACTCATCTTCAGCTCGTCAACCTCT
GAGGTCTCTGCTGCCGCCGGTGGTGAGCGTC
CGACGATCCAGCGCTGCACGCTACGAGCGAC
CCACCTACCGACCGGGTTCCGAACTGCGACA
CAGCCATCCGAGCCAATTCACCACGGTGACA
CTGATACGTATGACGCGCACCGCCCCCTCAC
ACCAGGCGTCTGTTTGACATAAAGAATCGAC
TGACTATTCAAGACAACTCTTACCACTCAAA
ACCCATAAGCCATCCGTGGCTGGGAAGGCAT
ACCGAGTGATGGCCATCCGTCGTTTATAAGA
ATTTTTATCTTTAATATTTAGTGGGCTGTAC
CATAAATTCCCAACAGATCCATCAATACGAA
GGCACCTCCTTCCGCCGAGCCACGCTCACGT
TGGCCCACCGGATCCTTATCAGGTGATCTGC
AGTAAACTTGGTATAAGATAACGATCTTCGC
ACCGTCGCAGGCCCTTACTCCGAGCCGTTCT
TAACCTGCCGATGTATCTGTTGAGATTGGTC
ATAATAACTGTTTTGATTGTGTTTTTTTATC
TCTTTCAAGTATCTTTCAGTCATAATCGCCC
GCATCCTCTACAGAATATTCAACTGTTCCGT
TGTTAACAACGTGATAAGGGTTGCGTATGAT
GTGCGGTACTGCGTGAACAATATTGAGTATA
TAACGTTAAGGTTTTCTTAGCATAGTATTTC
ACGACTCTCTCTCTCACTAACAACCATCGCC
ATCCATGTGCCGTTGTCAAAGTGAAATCAGA
ATGATACTGTATTGATGCCGTGTTGACGTTC
GAGTTCGCATACTATATCATAGTTAAGGTTC
TAAATTGATCAATACAGAAGTGGTTGTGTGA
GAAATCTATGCATAAGATCTGCTTCTGCGTT
ACTTGGTGACCTTAATAAACACACTAATACA
AATGTTAAAGCGATAACGGGGTGAACCGGGC
ATAACCACGAATACACACTCTGGGAATGTTA
TGAGACGAACCTAGACAACGATGCCGAGTTA
AATCTTGCATACTTACACCACACCTGCATAA
ATTAATTGCCTTATTCCGATTGATAACCCAC
CGAGGCGGGGTCTGGTTATCATAACGACTTC
AATTACACCCACTCGCATAATTAATACGATT
ATTGGCATAATTAAAATGTGCGTAATTACAT
ATTTCCGGATAATTACACATTTGGCCAAATT
TATTATTATTTTCACTGAATATTCATAACCC
ACATAATAATTTCACCCACGCACGCATACAA
CAAATTTTCATACCCAACATATACAACA

>ERV-Spuma.0-Pma

TATACGGCCCTTTTTCCACAATTAATATCCA
TTTACCGATGCATACACCTAGATTTCTTTTTC
GTGACAAACAATATATATTTTTAATAATGAA
TAAGCCAGAAACACGAAAAACCAGATGTCGC
GACCATTAATAAATAAAGAAATAATAAAACT
AGGATGTAACAGCTATCGGCGAAAATCTCCA
GAGTTTGATGGGCCCCTTAGGAAAGGTCCCT
GAACACCAAGAGAGCAGACACGAGACGCCAA
ATCGTATACATATTCTTTTCCTTATTTGCCAC
AAAAAAAAATTAAAAGGCAACAACAAGAAT
TAGCGATCCTMACGCTCACTCCAATTAAGAA
AGACACATCAGTGAATTATTATCCAATGATT
TCAAATGATTTTCACCTTTAATACTTTTACT
AAGCTTGGAGCAAGAATACCGAGTCACATCA
GAGAAGGAATGAGGCGAACGGGCAAACTTGC
TAAACAATATCCCAAGCAACCCCGACCACCT
TCACAGCGCGGAGAAGCAGGAATTACACCTT
GGCCATCTGGAAAAGCCAGCCGGGTTTGATC
ACCCCACCCCACACACGGCAACCAGGCAAAC

AAGAAAATTGCAATATAATATTAATAATAA
CCTTAAACGATATTCCATTATAGAACCTTA
GGATATGGATTCCAACAATCAAGAAACGGA
TCAGGATGAAAATTATTTTTCTTTTTAATAA
AACAATTCGAACCTCGGTAAACCGAGAGGT
CGTAGTTACAAACACGAGTCTTGGCGAACA
TGGTATTTGAGAAATATAAGTCGAAAGTAT
TCCCGACCACCTTTGTCTCTCCCTGCAGAA
TGCATACGCTCGGAAGAGCATCTTCACTTG
AAATACCCTGAATCACAAGCAGAATCATAA
TAMGATACCAACTGTGATGTGAAATGTTAA
TCTTAACCATTTCTTTGTGCGTCGGAACGC
GGGGGGGGGAACCCGCCCCTCTCTCTGGA
AGACAACCAGACGCCTTCAAGATGAATCGA
GTCTTGATGTTTATGGCTTGAGGAAAGACC
CATGTCATAAATGACACACACACAAACAYA
CACGAACCCCCCCACCACCGACACACGAA
CCGACACACGATCAGATCAAAGATAGCTCC
GAGGAGCCGAGAAGGGGAGAGATTTTTGGC
GCCGTTTCAGCATTGAAATCAACTCATCAG
CAGCTCAACAGAAGAAGACTCATCTCAACT
GAAGAAGACTCATCTCAACTCAACTCAGCA
TGACCTGTCGCCCGCGAGCGCAGCGTCAGA
GCGGAAAGAGCGCGCGCCGGCGCAGCTCAT
AGGAAACTGCTGTCAGCGAACCAGCACGGG
CATCTTCAAGGAGAAAGAGCGCGGCCAGCA
AGAGCGTGCAGCAGCTCATCCAGCAGTGGA
TGTCAGCGAACCAGCACGGGCGCGTTCGCG
GAGAGGGAGCGCGCGCCACCAGCGTGCCAT
ACCAGCACCAGCACCASCGCGCCGCTGCGA
AGCGCCATCTACCGACGGYCATCGCAACTA
TCTACTCGTCAACCAACGTCMGCCGAGTCC
CTGAAAGAAGAATCAACTGATACGTATGAT
CCACTACACGACAAACCAGGCGTCTGCTTGA
TCAATTCCGAACACTGGCTACTCTTACACCA
TTCAACACACACACACGGGTCATTAACAAA
CTCAAGCCATTGAACCATCCGTGGCCGTGGA
ACGACCGAGTGAATAATCCGTCGTTTATAA
AATTTTCCTCCTTAATATTGTGTTTGGGCTG
TCGCATAAATTACCAACCGATCCATCAATA
CTTGACACCTTCGCCACGGAGCTCCGCTCA
KAGCGTGTTCAACCGGTTCCCTGTCAGGGG
ATTAATTAAAAGATTGGCCCAAGATAAGGA
AAACATTGCGGCCCATTGAGTTACGTGGATA
GGAGTATTTGAGTCGTTCGTTTATTTTTCTA
CTTGCTGATGTATCCATGAATGAAATAACGA
CGCATATTTGTGCCTTTAGGAATAATAATG
CTTTTCAATGAATAGCGTTGCATAATAATCA
CTGTTAACAACGTGACAAGAATTGCATTAA
TGATCAATGACCGAATGCTGTGTTGTGTAA
AACGAAGCATTGCTGCCATTGAGTTACGTT
TTAGAAGTATTTCAGTCGAACGCTTTAATT
TATGAATCAATGAACGAACATCGCCGTGGTA
CGATTTCAAGCATAATAATTTCGCATATCA
AGGATTGGATACGAATCAAAGACCAAGTGC
AACAATTCAAGTATACGAGTTTGCATATTAT
GTTTGATAGAATAGTTCGCATGTTTTTAAT
ACAACCGATCGATTGCGTTACGCGTCGGGAA
GGTGCCGTTATCGAAGTGAAACAACGCAAT
TCATGTTAATGCTGTGTTGAAATTCCAAAAT
GAGTTTTCATATTATCCAAAAATTAAAGTTT
TAAAATTGATTAAAGCAGAAGGGTTGTGTG
TGAAATTCTGCATAATATTGTTCTGCGTATC
TCTTGGGGACCTATCATAAGCACACTATCA
TCCATAACACACATAATTCATAATGTTCAAT
TCGTATCCTTATCGATAATACGGAATTAAA
GAATCGACTCTCTCTCATTCACGCTCACAA
ACTGCATACGATTAATTGCGTTATTCCAAT
ATCCTCGTCACCGAGGCTGGACCTGATAAT
CTGCATTATCATAACCACACCCACTCGCATA
GATTATCTCTCGAATCAGCATAATTTATGC

ACTAATTAACATAATTTCGGATAAATTATGC
AATTTCATTTAATTTTCATTATTTTCATCAAT
ACCTAYATATACAACAWATTGGGGGCTCGTA
TCTCGCAGACAGTTTATGCTTGAATTAAGGA
TGTTGTATTAACCAAGGTATTATGAACTAAC
TGACTGTAACGGGTGTAAAACAAACAAAAAA
GAAGGGAAATACCAGAGTAGCAGGGTATAAC
TGGAGTAAGATTATTTGCTCGAGTCGAACTC
TGTGATACAAGTGTTTTTATACGTGCTCTGA
CAGAGTAACTCACGCCACACATGAGATGTTA
ATACACTCTTAAGTCTATTAACTACACTACAT
GGCACATGACCCGCAACATTCTGAGGTTGAC
CATAACACTGATACAGAGGGGACCTCCGAGC
AATCTATGGAAAAACAATATCGTGAATTTGT
TACTGGACGGACTGCTAGAGAGCAGACAGAC
ATATGGGAGGGGAGACATATAGAAGATGTTA
GGAGGAAACTACTTAAGGACGTGATGAAATC
CGAACGTGATGTAAAAATTGCAGAAATAGCA
TCATATCTCCTCACACAGACAGACAGAGAAA
ATGAGGAAAAATTTGAAAGGGAAAGAGAGGA
CCTAATAGAGGAGGTAGAGCGTCTGCACGCC
GAGAAAGAATCGGCGCTAACTGCACCAGCGA
ACGTTGATACACATAACACATACGGGAGTAA
ACACTCCGAGCCACGCGGACGTAGCCCAGGC
ACAAAAAGTATGGCTACCAATCAAAGTAATT
AAACCCCCTCGCAACTCGAGAGAAGGGAAGC
GATAAAAACAGGAAAACATGGGGCTGACAAC
AGAGAGAGACAAAATATTAAAACAAAGTCAC
CTGTACTGATAGAGCCAAAAAGGGCGGAGCT
CGAGCGACACAGAGACAGCTCACCAGAAAGA
CGCAAGTACGACCGACATGATGCAGATGACA
CACTAGGAAGACACATTCCACGCAAGCACGA
AACCTACAGCTCTCCAGAGAGACGCACATCA
GACCAATCAGACACAGACAGCTCACCAGAGA
CACGCAAACATGACCAACATGACACGGGTAC
CTCACCAGAGAGACGCACGACACGTAAAAAT
AAAACAACCCGCACCACAGATGACAGACACA
ACAGTCACGAAGCACGAGACCAACATAAACA
ACCTAGCAGACACCACACACGTAGTAGACGC
CAAGGACAAAAACACTCACCACATAGACACA
GATCACGCACACCAACCCACAAGCACGGATC
ACATAAGAAAAACAAGGACACAAGCCACGC
GAATCCTCGGACAGAGGGAGACCACGACACA
AACATGACAGTAGTGATAGCGAAGGCAGGGA
TGAGCACTCCTCAGGAACAGACGCCACTGAC
CACACTTTCAATAGTAAAACTCTGAAATTGA
CGCTCGTCAGAATTCCCTACTTCGATCCACA
CAGGGATATACACACTCACATCGCAACGGTA
GCAATGATCCGGGGTCTGAGTAAATCAGAAC
AGTTTATTGTCAAGGAGCTTGCATGAACCGGC
GGTAACGTCCCTGCCAAATAGGGTCAGGAGC
CAATTGAGCCGAGCAGTAATTAAGAGATTTC
CCACACTGTCTGAAGGATTGAACGCGGCAAA
TCAGTGTGCAGACGAGGACCCACGAGATTAT
TTCAAGAGAGCGTATTACGCAGGTGACGTGC
ACGAAGAAGAGAAAATATTAAAAGTTATGTT
ATTAAGACCTGAAATAATGAAAATAGTAGGA
GACCCCGAGACTGCCTCCTTACGCCAGATAC
CTCACACGGCCTGGGTGAACGGCCGGAGAAC
AGCCAAAACACACATCATCACCAAAGATAAA
GATCTGGAGGGAAGCGAATTTCCAAGAAATA
CCACACGGGACGGACATGAAACCCCCCAGGA
AAATCCACTAGAATCAACACCCATCAGCAAC
AACGTTCCACGTGGGGAGAGAAGCGAGGCCA
GGCACCCACGGCCGCCACAGCCCTGGAATAC
CGGATCTTGGAACTATGGAGGAGGGAGAGCC
TCATACCTACCCCGCGGAGGCCGACGCCCAC
TCACCATATCGTCTCCCACTGCCGCCTCCACT
GCATAGACCACAGTCAGCCAACAGGAGTCGA
CACATTCGGATGGAAAGAGAACACCAAGAGA
CTCCCATGCAAACAGCAAAGGCAAGAATACC

CACACAGGAAGAGGACGCACCCAACGCAAA
GTGAACATAAGAAACTCAAGAAACCCCAAA
AAGTGGTACCAGAGGGTAAAGGGTACCATCT
AATTCCCGTAGGACCGGTCGGACGAGTCCA
ATACTGCAAGACACCCTGACCAGCCAGGGC
CTGGAGCAGAAATATCCATGATATCCAGAG
TGAACTCCAGACTGCCCTCCAGAGGAAGAA
CAGATGATGCCAAGTAGGCTTACCTACTCA
GGACCCACATGCGACCATTGGGCAAGGTCA
CACGTTCAACGAAATCAACATGAGCCATCC
GTACAGAACCCAGGAAAAGAGCTGATCATT
TACTGAATCGCCTTAAACCCATTATGAACC
ACAAATATGGGCACAGTCAATGCCGGAGCA
CCAGAAGATGAAGAAGTCGGAAAAGACTCT
AAATGATAACACAAGAACTCAACGACCTGT
CAAGAACGAAACCAAGCGTCCAGCAGAAAT
CAACTACTGACCAACAGACTGGGAAAGGAC
CCGACCTACGACCAGTAGTTCCCAAGGACT
GGAGTATATACCGCCCACTAGTGGCCAAAC
GGGACTGGTGAAGGAGAACCCAAGGCCAGG
CCTCGGGTGAAGCAAAGGCAATTCAACCGA
AAGGAAACATCAAACTGAGCAACCAGGAAT
TTAGACCCAGACCAAGGAAAAAGAGTCCCC
AACAAATTGAACAACAAATTGAAGCGGCAG
GAATGACACTCAACGAAACGAGCTCCGACA
AAATACCACTTCCTGTTCTCAGAAGACTCC
GCAAAACCGACCTACATGTGGTATCTATTC
GGGAGCACCAGCGGTATACGTGAAACAATA
CTGGCCGCCTACGAATCAATCCAAGAAACC
TACTCAAGAAGAATATAATTCGGAAATGCA
CAACTCACCAATTTGGCCTGTGCTAAAACC
TGGCGACTCACCATCGACTATAGGCAACTC
TTCCCCTATCTCGATGGCCAATGGCAGAAA
TCTCAACCAAATAAAAGGCGCAAAAATACT
GACCTATCAAATGGCTTCTGGACCATGCCC
CAGATCAATACAAGTTGGCATTTACATTCA
GTATACTTGGAACCGATGCCCATTTGGATA
CCTGCAGACTTTAACATATTCCTACACAAG
ACGCAAAAGAGCGAGGCACCATCGTCTATG
ACTGGTCAAGGACTCCTCATGGGAAGAACA
CTCCAGCATACCCTCGAGCAGTTAAAGGAA
AGATATCAATACAAAAGGGACAGTGGGCAC
GGACTACCTCGGGTTCCAGATCGGCACTGA
CCCCAAACGAAAAGACTTGAAGCCCTCCTC
CGCCCACCACAGTCACGACCCTTCGGAGCT
CTGCAACTACCTTCGACAGTTCGTGGACGA
ATCGCTCGACCCTTAGTCAAACTACTACAA
CATGGGAATGGGGACARGAACAGGAAGCCG
ATTAAAACGACAGATCACAGGGGCATCCTG
CCAGAGAAAGGAAAAGAATACTACCTAGAG
CAGATCATAGTATCAGCTCGGTACTGTACC
AGCAGGGAAACGAATTATTGCCTACGCCAG
CGGGGGGTAGAGACAAAGTTCTCCGAGTGT
TATTCGGAAACATCTGGGCTATACAACATT
GTTGAACGGAGAGAAAATCATCCTAGAAAC
TCCTTGGCGTACCTCAACAGTAAGAAGATT
GCGTAACCAGCAGCCGAATAGCCAATTGGG
CCAAGGCCTACCAATCACAGTAAAATACGC
AAAAGTCCAATGGCCCAAGGTCTTGCCGAT
GCACCCTAAACAGCGTAGAAGCATGTGCCG
TCCGGAGGACAACAGGCACCTGCCCTTCAA
TGTGCCGACCTACCAATGGTGTACATCGAT
AAATGAAGGAAAACAAACTCCACGCCGGAT
TTGGGAAACTGGCCCACTCACAGGGATTCA
CAGCTGGGACCAAAGTCAAACCAGTATGCA
GGGTACACATCGCCGTCCAAATGGCATCCG
ACAGACCATGGTGGTGTGCACTGATTCCAA
CACAGCTTCCTGCACCACCTACCCATATGG
GTATGAAGAACCATAGAAATAAACCAATTC
ACTGTTCGAGGCCATAGATACGATGGTTCA
ATGAAGATATTTTGGAAGAAGGTCAAAGGG

TACCTGGCCAAGAGAAAACTGGGAATGACC
CTAGCAAAAGTGGGAAGCCTGGAAGGCAC
TAGAARGCCAAACCGAAAGAGACGGACAA
CGAACCAAGACCCCGCCGTGATAAGACTGG
GGGAAGAACCCTGCAGAAGCTGGAACCAG
ACAGTGCACAGCCTTCCGAAGACCTAGTC
AGAGGACGAAACACTAAAGACACTAGCTG
GATCCAACAGGAAAACCAATCACTACCGA
ACAACAAAGAGCTCGAGACCATGCATAAC
GGTTCTCAATGCGAAAAGGGCTACTGGTAT
ARAGACGGTAAAACTAGATGGGTAGTCCC
GAGAAATAATGTTACAACACGCCCACGAC
CGGCCACCGATGCGAGATGAACACAGGAC
CAGGTGGCCTACTGGCCTAAAATGGGTGA
AAACATGTGTCCAACTGCCTCGTGTGTTGT
AACGACCTCACAGCATAGGGCACCCCTAC
AACAGACTATCCTTGGTCCAACCTACAAAT
GACCTGTCAACCGATCAACCAAAGGAAAT
TGACTGTGACCTGTGCATTCTCCAAATGGA
CCAGCACCCAACAACACCGCGGAGACGAC
TGATCAACCAAATATTCAGTAGGTGGGGC
AATCGATTCAGACAAAGGAAGCCACTTCA
CATCACAGAAGTGTGGAAGATGTTGGGAGT
CTACATGTCGCATACCATCCACAATCCTCA
AAAGAGCCAACAGGACCATCGTGAGCCTCC
TGTGTCCACCACGGGACGCGACTGGGATAC
CTAGTCTTGATGGCCATGCGAGCCACACCC
AGGCAGGACACCATTCGAGATCATGACAG
GACCCTCCCAGTGCACCTGCTGTATGTCCC
GAGACCCCGACGGCAATGACGCCCACTGAA
GACTGAACAGTCACCTACAGAGCACATTCG
GACCAGTTGCAAGCAGAGGCTCAGGGGAG
TATGACCAAAAAGCCAGCCACGCAGAATAC
ACCAGGTGTTGTACCTCAATTACGAGAAAA
GTGCAGGAAATTCCTCCCTTCCTGGACAGG
ATTGTTGACAAAATGTCCCCAGTCGTATAT
TCACAAAGACAGGAAGAGAGCAGCAGTACA
CATCAATCAAATCAAACCGTACCCTGTGCT
AGAAAGAATAAAGAGAAAAAAAGGGGGGG
TAAATTGGGCATGTCACACTAAGATAATGT
TCCAGGAAATGGCAGCCCTCCTGCTCACGC
TTGCTTTGCCAAGCTAGAGGGCAGCCAAGA
CAGGACCAGAATCCGGGGTGATGCTGAGA
CTTCCTAGTGACCGAAACGAAAATAGTAA
CTATGTTTCCTTAGAACCTGCCGTGGTARTA
TTCGGGAAGATACCACCCTCAACTACCAGA
ACTGGTACCATGACTTCCTAAGATACAGTG
GGAACGATCCTGAAGCAATTACAAAGAAC
CCCCCACCACTGTCAACACAACACGTTCC
TCGGCCTGGGAGGGGCCTTCGGAATACTAT
CTCCTTAATCACACTAGGAGTATCCATATC
AGATTGGACACCTCACGCAAGGCGTCGAG
TCGATATGCATCAAATCCGTCAGCAAATCC
AAAGAATCTGATCGAAATGGGATCCACCCT
GTGGTTCTCGTTAACCTGCACTCCGCCATG
AATCCAAGAATTGGGTTTCGCAATCCGAG
GAAGAGAATACTCGACAGCCAATTCAATG
ACTTGTTGCGTAGCACCAGCGGCAGCATC
TCCAAGGACAAATTCCTTCATATCTGGTAA
AAAAAAAATGTGCTACAAGCAGCATCCCT
AGTCCAACACAAGTACAAATTGCCTTTAAC
CACACCCATACATGTGGACCCCCAGACCA
ATTTTTGTTAAATTTACCACTGGTCAAACC
TTTCGATTGCGGACAGTATTGAACGTGGGT
ACGACATTTACTCTAAGATGGAAACCCCCT
CTACCAGGAAGAGGGCCCCCTTAAGTACCT
TAAACATGTGCCAAATAATGAAAGAGGTT
CCCAGGGGAACCATTCCTAATCAATACAG
GTGCGGTCTGTCAACCCTAAGCTCCAGGGA
TTCAAGCTAAGTAAACCCAAAGAAAGAACC
CCATGATAGCCGATGCTCAGTGGTTAATAA

AGATACGGCAACCGTTTCCACCGATCAACAC
ATGAAAGTTCCGATTCCAAGCCACGTCGCAC
TACCAAGAGGAACATTCGTACACATAGGGG
ATATAATTTAGACAAAAGCCTACATCAAGC
ATAGTAAACACATTCCGAAGCCACAAGTTT
GCACTCTGTCTAACCTGTTGAACAGTAAAG
GGTGAAATTAGCCATCAGCGAGTCAGAGAT
GATCTCACTTGGGCGACCGAAGAGGCCGGA
ATCAACAGATGGGACTGGGAATCACAGGGG
GATCATACTAGCCATGGCTATCCTAACCCT
ATGGCCAAAATGAATGCCGAACTTAAAAGA
GCACAAGCCACATACCATTCGAGCCCAGGA
AGTCAAGGCCTGACGATTCCCCCCCAGGAA
AAATTTTATTAACAACATCCCCTATAACAT
GAATACTTCCTTTTTTTTTACAAAACTTTT
CTTCTTATTTCAAATGTAATCATGCTAAGTT
ACGCGCGCGTTGTAAGGAAAAATCACAAAC
ATGATGGAAAAAGGCATTTTTCTTTTATTT
CCGTGGCAGACCACCGATCATTTTTAAAAG
TGTATATGGCCCTTTTTCCACAATTAATAT
ATTTGCCGATACATACACCTAGATTTCTTT
TCGTGACAAACAATATATATTTTTAATAAT
CGTAAGCCAGAAACACGAAAAACCAGATGT
CAGGCCATTAATAAATAAAGAAATAATAAA
CGAGGATGTAACAGCTATCTGCGAAGATCT
AAGAGTTTGATGTGCCCCTTAGGAAAGGTC
CAGAACACCAAGAGAGCAGACACGAGACGC
AAATCGTATACATATTTTTTCCTTATTTTCC
CAACTAAAAATTTAAAATGCAACAACAAGA
ATTAGCGATCCTCACGCTCACTCCAATTAAC
CCAGATACATCAGTGAATYATTATCCAATT
TATCAAATGATTTTCACCTTTAATACTTTTA
CAAAGCTTGGAGACAAAATACCGAGTCACA
TCGAGAAGGAATGAGGCGAACGGGCAAACT
TTTAAACAATATCCCAAGCAACCCCGACCAC
CTCACAGCGCGGAGAAGCAGGAATTACAC
GGGGCCATCTGGAAAAGCCAGCCGGGTTTG
ACACCCCACCCCACACACGGCAACCAGGCA
AAAGAAATTGCAAAATAACATTAATAATAA
TTAAACGATATTCCATTATAGAACCTTACAC
ATATGGATTCCAACAATCAAGAAACGGACC
AGGATGAAAATTATTTTTCTTTTTAATAATT
CAATTCGAACCTCGGTAAACCGAGAGGTGT
TAGTTACAAACACGAGTCTTGGCGAACATG
GTATTTGAGAAATAAAGTCGAAAGTATAAA
CTCCCGGCCACCTTTGTCTCTCCCTGCAGCAC
TGCATACGCTCGGAAGAGCATCTTCACTTG
AAATACTATGAATCACAAGCAGGATCATAA
TAAGATACCAACTGTGATGTGAAATGTTAA
TCTTAACCATTTCTTTGTGCGTCGGAACGT
GGGGGGGGGGGAACCCGCCCCTCTCTCTGG
TAGACAACCAGACGCCTTCGAGATGAATCG
TGTCTTGATGTTTATGGCTTGAGGAAAGAC
CCATGTCATAAATGACACACACACAAACAT
ACACGAACCCCCCCCACCACCGACACACGA
CGACATACGATCAGATCAAAGATAGCTCCG
GGAGCCGAGAAGGGGAGAGATTTTTGGCA
CCGTTTCAGCATTGAAATCAACTCATCAGC
AGCTCAACAGAAGAAGACTCATCTCAACTC
AAGAAGACTCATCTCAACTCAACTCAGCAG
TGACCTGTCGCCCGCGAGCGCAGCGTCAGA
GCGGAAAGAGCGCGCGCCGGCGCAGCTCAT
AGGAAACTGCTGTCAGCGAACCAGCACGGG
CATCTTCAAGGAGAAAGAGCGCGGCCAGCA
AGAGCGTGCAGCAGCTCAGCCAGCAGTGGA
TGTCAGCGAACCAGCACGGGCGCGTTCGCG
GAGAGAGAGCGCGCGCCACCAGCGTGCCAT
ATCAGCACCAGCGCGCCGCTGCGGGCGTGC
GTCTACCAGACGGTCATCTCAGCTACAACA
TCGTTCACCAACGTCCGTCGAGTCCGAACC
AGAAGAATCAACTGATACGTATGATACACT

ACACGACAAACCAGACGTCTGCTTGACGCAA
TCCGAACATTGGCCTACTCTTACACCACTCA
ACACACACACACGGGTCATTAACAAAGCCTA
AGCCATTGAACCATCCGTGATTGTGGAAACA
CCGAGTGAATAATCCATCGTTTTTAAGAGTC
TTTCTCTGTAATATTGTGCTGGGGCTGTACT
ATAAATTACCAACTGACCCATTAACAAGGAT
GCACCTCACCACGGAGCGCCGCTCGCGGGGA
TGTTCAACCGGTTCCCTGTCAGGGGTCTGGC
TTAAAAGATTGGCCCGAGATAAGGATCTCAA
TCGCCACCCATTGAGTCGCGTTGAGAAGTTC
CACTTGAGTCGTTCGTTAATATTTGTCTTTCT
TGCTGGTATCCACGAATGAAATAACGAATTC
TTTGTGCCTCCAAGAATAATACTGGTTGGAC
AACGAGTAGCGTTGCATAATAATCAGTCATC
ACAACGTGACAATAATTGCATTAAGGGCCGC
ATGACCGAATGCTGTGTTGTGTGACAATTCA
GCATTGCGGCCATTGAGTTACGTTGATGAGC
TTATTTCAGTCGAACGCTTTAATTGCATAAT
ATCAATGAACGAACATCGCCGTGGTACTGTC
TCAAGCATAATAATTTCGCATATCAGAATGT
TGGATACGAATCAAAGACCGAGTGCCGTGTT
TTCAAGTATACGAATTTGCATATTATAAGGT
GATAGAATAGTTCGCATGTTTTTAATCTCTC
CCGATCGATTGCGTTACGCGTCGGGAATTAA
CCGCTATCGAGGTGAAACAACGCAATCGTGA
GTTAACGCTGTGTTGAAATTCCAAAATCCCC
TTTCATATTATCCAAAAATTAAAGTTAGCTA
ATTGATTAAAGCAGAAGGTTTGTGTGTGATT
ATTCTGCATAATATTGTTCTGCGTATGAGAT
GGGGACCTATCATAAGCACACTATCACCACA
TAACACACATAATTCATAATGTTCAATCGAC
ATCCTTATCGATAATACGGAATTAAAGAGCA
CGACTCTCTCTCATTCACGCTCACAACGCCT
CATACGATTAATTGCGTTATTCCAATTCATA
TCGTCACCGAGGCTCGACCTGATAATCCATA
ATTATCATAACCACGCCCACTCGCATAAATT
ATCTCTCGAATCAGCATAATTTATGCGCATA
ATTAACATAATTTCGGATAAATTATGCAAAT
CATTTAATTTTCATTATTTTCATCAATTTTC
AACACCTATATATACAACATGTC

>ERV-Spuma.a-Etr

ACCCATGGTGGACGGCGTGAAGGCCGAGCAC
CACCACACAGGCACGAATCAAGGAGACCACC
ACATAGACACGRGTCACGAGACCAACATAAC
ACACAAGCCAAGAGACAGACACGACTCGTCA
AAACCACGACACAGACAAAGGAAACGTGATA
GCAGAGACAGAGACAACTCGAGTGAGCACTC
AGACKCCWCCGAYGAYGACCAACACTCTTTC
ACYCTCAAGTTGATGGACAAAGCKCTCGTGA
ATTTTGATCCACACAATAAGAACAGAGATAT
CATCGCAACAGTAAAAGMAGAGGCAATGATC
GGTAAGTCAGAGGAGAAAGCATGCTTATTGT
TACATGAACATGCCAGACCATGGGTAACCTC
CAAAGTCAAGAGCGATTTCAAACAATTAAGC
ATTAAAAGATTTGGGGAATATTCCACCCTAT
TGAACGCGGCAAAACTAATGACGCAACGTGC
CCCACGAGATTATCTCCTCCGACTCAAGAGA
GCAGGCGATGTAGACAACGCCCAGGAAGAAC
TAAAAGTGATGTTCTTCCAATCACTGAGGCC
GAGAATAGTAGGAATGATGTTAGAYCCCGAA
CTACGCCAGATWGAAAGCAAGGCCCACACGC
ACGGCCGGAGRACCACYAACRTAGTCAAAAC
CACTAAGGATAAAGAACGCCTCGACCTAGAC
TYYCCTGGAAACAAYARCAGCATCACACGGK
GAACCCCCCAGGAGAGGGCGCCAAAYCGACA
ACCCACCAGCCAMCYACTGAATAATGRATCT
GCAAGAGCAARCTGGCACCCSCGACAACCAC
TGCCGAMACARGCGAAWYCTGGAACAACCM
GAGAGCRARYGATGGACCACMCACAYCCCGC
CGCCCACCAAACCAATCACCATACCGCCTCC

CTCCACTAGCCATAGAGCATAGACCATCAC
GAATCGATATGCTCACCACATCCGGATGGA
CTAGAGAATAGGAAGACTCCCATGCAAACA
GGACATCAACAGAACACACGCAGGAAGAGG
CGCAAATATAGCCCAAGTGAACATCGGAAG
CCCGAAGCAGCCACTGAGGTGATACCCGAG
ACCATCTGGGAGAACTAATTCCCGTAGGAC
AGTCCACCTGGAAGTAAGACTGCAAGACAC
CAGGGCCTGTGTGATACCGGAGCAGAAATA
CCAATCACCTATTTGGTGAACTCCAGACTG
GAAAAATGAAGCACTACAGATGATGCCAAG
TACTCAGGATGCGAAGGAACCCACATGCGA
AAGTCATGATGAAGCTCACGTTCAACGGGA
CCATCCACTATATGTCGTACAAAACCCAGG
ATCATTGGGATCGAACTACTGAATCGGCTA
TGAACCTTGAGTCCCGACAAATCTGGGCAC
GGAATGGCATAGAGATCCAGAGGACGAAGA
AGCCCCACGGGAACCCAAGTGCTAGCACAS
ACCTGCCACCGACAASCAAAGACAAAAACA
TGAAATACGAACCACTCAACTAATAACCAA
AAGGACCTTGACGAAGCCAACCTACGGCCG
AGGACCATCACTCCAGAGAGTATCGACAGC
CCAAACAGGTGAACAGGAAACAAAGGGAGA
AAGAGCTCATCCCCTTCGAGTGAAGCAAAG
CACCACTAACGCAAGGAAAACACCAAACTG
AACCACGAAGGCCCTAGATCCAAACCAAGA
CTCACAATAGAAGAACAAATAGAACAACAA
CAGACGCCCTAGAGAATGACACTCAACGAR
ACAACTGCTCCAAAAATACAACTCCCTGTT
TCCTATGATTGYGGCAAAACCGACCTACAT
TTCCCACACTRGAGGGRGCACCAGCAGTAT
ATACAAGATTCCACTGGCCGCCTATGAATC
ACCCTGGAGAACCTACTCAAGAAGAACATA
GCAACTCAACCTACAACTCGCCCATTTGGC
ACCCACGGGCAAATGGCGACTCACAATCGA
CTCAACAAAACAGTTCCCCTATCTCGATGG
AAATCGACCACGGTCTCAACCAAATAAAAG
ACTCACCACAATGGACCTAGCAAATGGCTT
CCAGTAAAAGAAACAGATCAATACAAGTTG
TCAACGGAATACAGTATACTTGGAATCGCT
ATACTCGAACTCACCTGCAGACTTCAACAT
AGGCGATGGGCGACGCAAAAGAGCGAGGC
ACGTCGACGACATACTAGTCAAGGACTCCT
ACATCTCAAGAGCCTCCAMCATACCCTTGA
GAAGCAGGCGCAAAAATCTCAATACAAAAA
CACGAAAAAGGGTTGACTATCTTGGGTTTC
TGAGGGATTGTTACCCCAAACAAAAAGAAT
CTCGCACTCAAGTCCCCCAACACAGTCCCG
GTTTCTTGGGAATCTGCAACTACCTCCGAC
CGACTATGCAGGGATCACTCGACCCTTAGT
CAAAAGGACGAGCCATGGGAGTGGGGACCG
CCGCAGAAGAAGAATTGAAACAACAGATCA
CTGCCTAGCATATCCCGAGAAAGGGAAGGA
GAGACTGCCTACTCAGACCACAGTATCAGC
ACCAGAGACAAGAGGCGGGGAAACAAATTA
CAGTAAAGCACTCCGGGGAGTAGAGACAAA
TGTGAGAAGGCAATATTCGGAAACATCTGG
ATTTTCGGAACTTGTTGAATGGAGAGAAGA
AACAAATCATGAATCCTTGGCATATCTCAA
ATTCGGGAGGGCCGCGTGACCAGCAGCCGA
GGGCACTGACCCTCCAAGGTTTACCAATCA
CGCAAAAAATAAGAAAAGCCCAGTAGCCCA
GATTTACATGATTGCACCCTAGACAGCGTA
ACGTAGGGGTACCTCCCGAGGACAGCCGGC
TGACAAGGAAACATGTGCCAACATACCAAT
GATGGATGCGCCAAAATGAAAGAAAATAAG
GATCAGGAATCATGTGGGAATCCGGACCAC
CCAAGAGGGGTTCCAGCTGGGACCAAAGTC
GCAGAGCTGGCTGGGGTACACATTGCCCTC
CCGATAACAAGATACAGACCATGGTGGTGT
TAACTACGTACAATACAGCTTCCTAAACCA

GGAAAAAAAATGGCATGAAGAACCATAGG
TCCACCATCAAGAACTGTTCGAGGCCATAG
CAGCAAAACGACATGAAGATATTTTGGAG
GGCACTCCAAGATACCGGGCCARGAGAAG
ACCGAGCAGACGCCCTAGCAAAACTRGGAA
ACCCCATGGAAGCTAGAAGACCAACCCAA
TAGAAGAGTCRACGAACTCAGACTCCGCC
GGTCACAAGAAGCGGAAAGAACTCAGCAG
CAGAGCTCACTTGARCAATGCACAGCCTCC
TCAGAATGCAACAAGAGGACGAAACAATA
CGACCATATCCAGGATCCGGCAGGAAAAC
GACAACTTGGACAACAACAAAGAACTCGA
ACCAAATCAGAAGATTTTCAATGCGGAAA
ATACACAAACGAAAAAGACGGTAAAACTA
TCCTTCGCAATATCGAGACACAATGCTACA
ATGAACCATGTAGCGGACATCGGTGCGAG
GACAGATGTTGCGACAGGTGGCCTACTGGC
KGAAGACGTACACAAGCATGTGTCCAACTG
GCAAATACCAGCCAACGAACTCACAACAC
TACAGAAAAGGGGAACAGACTTCCCTTGGT
ATCGACTGGATAGGACCTGTCAACAGATC
AACAAATACATGCTGACCGTAACCTGTGCC
GGTAGAGTGCTTACCAGCCCCCAACAACA
GACAGCTACCCTACTGGTCAACCAGATATT
GGCCTACCGTCAACAATCGATTCTGACAAA
TCACAGCCGATGTAATCGCAGAAGTGTGGA
GTAAAAAGACAACTACATGTCGCATACCA
CAGGACAGGTGGAAAGAGCCAACAGGACC
TCCTAAAAAAATATGTGTCCACTACCGGAC
CACAAAACTACCCCTAGTATTGATGGCCAT
CCAACCGGGCGACAGGCAGGACACCCTTC
CGGGAGACAAATGACCCTCCCAGTACACC
CCAGCGGAGGACGAGACCACGACGGCAAT
AATACATAACGGGATTGAACAGCCACCTA
TCGCGATGGTCAGAGACAAATTGCAAGCCG
AGCAAAGCATACTACGACCAAAAAGCCAG
TATCAGCTAGGAGACCAGGTGTTGTACCTC
AAGGAAACAAAAATGCAGGAAATTCCTCC
GGACCATATGAAATTGTTGACAAAATATC
ACCAATTAAAAATTACAAAGACAGGAAGA
ACAAGTGGGTACACATCAACCAAATCAAGT
CAGATGGAACCAGGGAAGTAATCGTAACR
AGRGGTGGGAGARACTAAGAAGTGGGGATG
CAACGTATAAATGTTTTCCAGGAAATGGCA
TCACGCTCCTGCTAACGTGCATTGCCAAGC
CCAAGACCTACTCAAGCCAGGACCAGAATC
CTGAGAGATTCTTCCGGTTTCCTAGTGACT
TAGTAACCCAACAAGTCTATGTTTCATTAG
ACAGTAGAGAAACATTTCGGGAAGATACC
CCGTAGAAACCCGAACTTGGTACCATGGC
CAGCGAACTGAATATCGGAACAATCCTRG
GAGAACCATGATACTTCCCCCCRCCACTTT
CGTTCCAAGAGATCGATCGGCCTGGGAGTG
TCCTATCCGGCTTTAGCTCCTTAATCACAT
TATATCCAATGCCGTGAAAATTGGACACCT
ATCGAAAACTTACAGCTCGATATGCATCAA
AATCCAACACCAACAAAAAAACTTGATCG
CACCCTTAGAGACACCGTAGTTCTGGTCAA
CCATGATGAACTTAACAATCCAAGAASTK
TCCGAGAACTATACGAMGAAGAAAATATTC
CCAATGGGTAATCCARGACTTGTTGCGTAG
AGCATCAGTGATCTAATCCAAGGTCAAATT
TGGTAACTCCGACGCTGATACAAAACGTGC
MTCCCCAGAAAGAATCAGTCCAACACAAGT
TTTAACATGGGTACAGCCACACCTATATAT
AAACCAGACAACTGGCGTTCCTGTTAAATT
AAACCCGAACAGGTATATCGATTACGAAC
TGGGTACATGGAGAAATGACATTTACTCC
CACCCTCCGTAGTGGCCTACCAAGAAGAAG
TACCTAGTGCCCGACCTAACCATGTGCCA
GATGTCCATTGGCTCTGCCCAGGGAAACCC

ATACAGCTGAAATAATGTGCGGCCTGGCAAC
CAGAGAGACCTGCAAGTTCAAACTAAGTAAA
AGAACCGAAACAGTGGCCATGATAGCTGATC
TAGTCAGCACGCCTCTGGATACAGCAACCAT
TCGACACTCCGTTACCATGAAAGTTCCGATA
GTTGCACTAGTCACAGTACCAAAAGGAACAT
TAGGGGATAAGGTATTATATAATTTAGACAA
CCAAGCAGATGTAGAAACAGTAAACATATTC

>ERV-Spuma.a-Lre

GGCTGTTACAGAGGGGCTGTTTGTGTAGGGC
AGGAGCTAACGGAAAACAAACACAGTGGGCC
GAGTGCACGGGGAGGTGCCCTCTACGACGTC
CACCCTGGGGGGGGGTTTCGATCCTCTAGGC
AGGGGATGAGCCAGAGGGCTCGGGGCTCAGT
GATAAGGGAAGACTGAAGACCGGCTAGATAC
CTCCCCTGGTTCGCCGTCCTTGTCAGGGCCC
GAGCCAGCGGCGTGGTCCCGGAACCAGAGTC
GGACTCCGGGTACCGTAGCCCGACTTACCAC
AGATAGCCTCCCCTAGCCCGTTAGTGGAGGA
GAAGGATAGCTAGGGGTGTATCCCCAGTTAT
GGGGCCGATACCCCCTCCAGTGTTGTATGGC
AATAAATACGTGTGCCTCACCCCGGTGTCCA
AACGGTACTTCATCTGGTGTCAGAAGTAATA
ACACGCGCCAAACAGCTCGGGCCAGGGTACC
AGACACCGCCGAGATCGTCGAGCCCGAGACC
GACAAGGTGCAGCCGGGGCCTGACGCCTCCT
ATCGGCATGAAGAGGAGCCGCAGCCAGACGC
GGCTCACGCGAGGCTGGCGGAGCTGCTCTAC
GCCACGCTGGAGGACATCACGCGGCTGGGGC
CACCGCGGGGCGAGGGGAGCCGACGACCGGA
CGCCATTTCGGCGCAGTCCGAGTTTGAATTT
GACGCAGCGGCTGCTGAGGGCGTGGCCAGCC
GGTCCGACATCGGCCTCGCTGAAGCTGCGAC
GCCGGACGGCGCGTCATCACCGGCGCGACAC
TCGGCCGTTCGCGCCGACGCACAGCCGAGTC
GCAGCCTGCGGGATTCCACAACGACCGCTGC
GAGACTGCCCCCGCTGAAAGAATTCGTCGGC
GACTGGGGCGGCTTCCAGAGACGCTTCCTCC
AGATGGCGAAATGGACGGACGACGAGGCTCT
GCCGGCGCTCCTCGACAGCGACGCCCTCGCC
TCGGCGCCGAAGGAGAAGCGCGCCACGCTAC
TGCAGCTGCTGGCCGCGGTCTATGGACCGTC
CAGGCAGCTGTTCTACGATCGCCAGCGGGGC
TCGCCCCTGGCGTACCGGACGAGTCTCCTGC
AGGCGGCCTTCCCACGGATGGACGACGACGC
CATGGTGACGGAGAAGATCCTCCTCCTGGCT
GACATCGCCGTCGTGGCACAGGAGGACGCGC
CCCTCCAGGCGGCGCGGCTCCTCCACGCCAA
CCTGCGGCGGAAGGCCAGCARGGCAGCGGCC
GTGGCTGCAGCCACCCTCCCGTCGGAGGAGC
TCGACCACCGACGGCAACAGGGGACGGGAGC
TCGGGACGGGCCGAGCCGCCTCGACCATCCC
GCGGCGCTCCCGCGCTGCTACAACTGCGGCC
ACGTCGCCTCGGGGTGCCGCGCTCCTCGTCA
GACACCTCGACAGGACGCCGGGCCTCCTCCC
CAGCAAAATCCGGCGAGTCGCGGCACCCCAC
CCACGCACACACCCGTCACACACTCACGCAC
CGGGGGGCCGATATCGCGCACCGTCGCGCCA
ACTATGGGGACTCAGATCGAACCCGGCCAGT
TGGCCCATCGTGTGGTTGCCGCGTCGTCGGC
CGTTGTGGGACACATTGATGGGGTTGAGGTC
GTCGACACGGGGGCATCGGCGACTATTATTT
TTTATAATATTCTCCCTGTCCAAAGTCGTCC
TGTTGACGTGCCATTCTATGCCGCTAACGGC
GGCATTATCGGACAAATTCGGGCGCAAATTT
ATATTAAATTGTCGGGGCCGGTTTTTGTTTC
GGCGGTTCCGTGCCTGTTGGGGACGGATTTC
ATGCCCATCAGAATTTTGATCGACCAGGGAT
TCCCATCGGGCCGACGTATGGCGTTTCTGCC
TGCGATTCGGACGGCGTTCGCGACGGTGCAC
GCAAAAACGGTGCGCGTGCCTCCGGGGGCAC

TWCCTCTTAAACTTCGTCGGCCCTTCGCTAA
GGATCAGGGGGTTCTGTTGGGACCGTCTAGC
AATGGACCTGATTTACTTCCGGCCCAGACCA
GGGATGGGGACCCCTTCATCGCGGTCCTCAA
GCGGACGGTGGTGGTTCCTCAAGGGACCGTC
GCAACGCCGGTGCCCTGTGAGGAACCCGTCC
TCAGCACTGTTCCTCCGTCGCGACCGCATTC
TGAAGGGTGGCTCGAKGCGCTTTGTGCGGGA
CTGTCCGATTCAGAGCGTCAACAGCTTAGAA
GGGAATTTTCCGACGTTTTTAGTCAACATAA
GGGTTGTACTAACCTGCTGCGTCACCATATT
GACAGCGCGCCAATCCGCCAAAATCCCTTTC
CCGCCGAAAAGGACCATGTCAAATCGGCTGT
GTTAGCCGCCGACATCATTTCTCCTTCCACC
GGAGCGCCGATCGTCCTGGTTAAAAAGCAGC
TGCGGTTTTGCGTCGATTTTCGGCGCCTGAA
GGTGGCCGACGCATACCCGCTCCCTCGGATC
CTGGACGCCTTGGCGGGAGCGCGATTCTTTT
GATTTACTTTCGGGATTCTGGCAGCTTCCTCT
ATCAAAGCCTAAAACTGCTTTCCGTACACCC
TTCCAATTCAACCGGTTGCCGATGGGCTTAC
CGGCAACTTTCCAGCGTTTGATGGAATTGGT
ATTGCAATGGGATTCGTGCCTGATTTACTTC
ATCGTCTTTAGTAAAACTTTTGACGAACACC
TGCGGGCGGTTTTCCTCAAAATGCGGGCTGC
ATTTAAACCCCAAAAATGCCATTTGTTGCGC
CGTTATTTGGGTCACATTGTCGGGCGCTCGC
CCGATCCCGCCAAAACGCAGTGTGTCAGCTC
TCCCACCTCGGTGGGAGAGGTCAGGAGTTTC
GCGTCGTATTATCGACGGTTTGTCGCTGATT
TTGCGAAACCCCTGCACGCTTTAACGTCCAA
GTTCCACTGGGGTCCGGAGGAGGAGGCTGCT
CTTCGGGACGCCCTGGTGAGGGCTCCTCCCT
CCGATTTTTCGAAACCCTTCTTGCTCGACAC
CAACACGGGCATTGGGGGCGTGCTGTCCCAC
GGCGTGGAACGGGTGGTCGCGTACGGTAGTC
CTCCGGCGGAGCAGCGATACTGTGTCACCCC
TGTTGGCAATTGTTTATTTCATCCAACAGTAC
CTGTACGGTCGGCATTTCACCGTCCGCACAC
CACTACAGTATGCCCTGCGGGTTCAACAACC
GTTGTCTCGATGGTTGGAGGTCCTCCAGGAC
GAGACCACATACCGTAAGGGCGTTCGCCACA
ACGCTCTGTCGCGCATGCCAACCACCTCGGA
CCTCCTGGGGTCGGGGCGTTGCAGGCCTCCA
TCGGTTCCGGTCCTGGCGGCCGCCGCCGATC
TGGCTGCTAATTCGATCGAACAACAGGGAAC
GCTGGGGTTGCCCGTCACTGAATGGGGGCGC
GAGGACACCTCCCTCCTCGCGTTGCGTCGTC
GGGCTGGGCGGGGGAAGGGTGATGCTGACTC
GGTCCAGGGTCTGCTGATGCATTGGGACGGC
CGACACCGGCTGGTGGTCCCTTCTGCGTTGC
TGGTGTGGGCTGCTCATGAGCGCCTCCTGCA
TCAAAAGACCCTTGCCATGCTCCAGCTTGAT
CCGGGCATGACAAGGGATGTGGGGGATCTCT
GCCCGCAGTGTGCGCAACGGAAACCACCAGC
TAACCCGCCGTTGCAGTCCATCAAGGTTTCC
GAGCTCGTCGCGCTGGACATTTTGGGCCCAT
CCCCCAGGGGCAATAGGTATTTGTTGACAGC
TTTCACGCGCTGGCCTATGGCCTGGCCCCTC
TCGGCAGCAGCCATCGCGGAGGCCTTTGTCC
CCTGGACAAAGGTGCTCCGGAACGGCTGCTC
GGTAAAAATTTTTCCGGTAAACTCCTCCAAC
ACCTCCTGGGTACGAAGAAAATTCGTACCTC
CCCGCAAACTGACGGGATGGTCGAGCGGCTC
ATCACGTCCATGCTGTCCCAGCAGGTGTCGC
CTGATTGGGACCTGCACATTCCAGGGGTCTT
CAGGATGGCTCCCCATGCGGCCACGGGATTT
TTCCTAATGTATGGTCGCGAACCTGACCCGC
CCCAGCTGCAGATCCCCGCGCCGCAGGGGAA
GGCCGACCACGTTAAATTTAACCTCGCTAAA
GCGCGGGATGCTGCCATGCTAAATTCGGACC

AGGCGAACGAGCGCCTCAGGCTGGGTCGGG
CCAGTGGAAACCGGGGGACAGGGCATGGTT
CAGGTGCCGCTGGCAACCTCCTCTAAGCTGC
GGCGGGGCCCTGTGGAGGTGGTCGAGGTTT
GTGTGTTCGCATCAAATGGGGGAAGCGCCG
CACCCCTCTAGGTTAAAACCCTTCGTTGCG
CCACTTCACACACGCGTGGCCAAACGTGTG
CGACTCACTGGGTCCGTCTCCGCTGCTCTC
TTGCCGGCTGTTCCACCTCCAGGTGCATCA
GCTCACGCCCGACAACCCCTCCTGGGGATG
CGCGGCATCGAAGGCGGACTCACTCCGAGA
CCACCCGACGGTCCGCCACCCGGGGTTGCC
GGCCCGCCGCAAGGACGCGCGCAAAAATGC
GTGAGGCCGGAAGATGTGTACATATGTTGT
TGTTGTGTGGGTGGTGTGTTCCCTGCCCAT
TACGTTGGGTTCACCTGGTGTTGTGTGTAT
CTGCAAATTGTATATAGGTTGTTTTTACGG
TATTCAGGGGGGGTGCGTCGGGTTGTTTCC
GTTGGTTTTGTTGTTGTTCTTGTTGTTGTTG
TTGTTGTTGTTTCCTTCATGTTAGTTAAAA
AGGCACACGCGGGGGTCAAGGGGTTAATT
GAAAGTTGTTGCAGCCGGGACGGCTGCGAG
GGGGGTGATGTAGGGCTGTTACAGAGGGGC
GGCTGTTTGCATAGGAGCTAATGGAAAACA
GCGGGGCTACGTGAGTGCACGGGGAGGTGC
GTCCGAGCCAGACACCCTGGGGGGGGGTTT
GGGGGTATAAAAGGGGATGAGCCAGAGGG
AGTTTTAGAGTAGATAAGGGAAGACAGAAG
TAGAGCCCTGGACTCCCCTGGTTCGCCGTC
CCGAACAGGACCGAGCCAGCGGCGTGGTCC
GTCAGTGCTCTGGGACTCCGGGTACCGTAG
CAGTGTCGGGCCAGATAGCCTCCCCTAGCC
GGACTCGGCTAGGAAGGATAGCTAGGGGTG
TATACGGGTGAGGGGGCCGATACCCCCTCC
GGCCACTGTGTAAATAAATACGTGTGCCTC
GTCCAAGCGTCTTCAAGAGTACTTCACAG

>ERV-Spuma.b-Lre

TGCGGGCGTAAACGACCTCCCGGATGTGAC
GAGGAGTTAGGTCACGTGGGGGAGAGGGTT
GGGAGGAAACCGGAAGAGGAAGGGAGAAC
GGACCAGAGTAAGGCCACGTGGGCAAAGCC
AGCGGCGGCCAGCGGTGGCCAGCTAAGGGA
GAGCTGCGAGAGAAGACAGCGGTGGGGCGG
GGCGGACGAGGAGCTGCGGGGAACACAGCC
TGTGTGAGGGAGAGGCTACGAGAGCGACGA
AGGCTGAGAGAACGACGAGGGGAGAAACTG
GTATCAGTGTCAGTATAGAGTCTTATAGAA
GTGGGTGAAGAGAGAAGAACCGAATAAAGG
AGACAGAGTGGTCGAGTCCTTTATTACACT
GTGAAACGGCATGTCGAAACAACTGAGAAA
CCAGCGCCGGCATCGGCTTCATCGTCCGAC
TTCCTCCAGAGGTGGATCCTGCCGGGGTGG
CCCTGCTGAAACGAGTGCGGGGCCGGGAGT
GCCGCGGCGTCGGACCCGTCTGCACCAAAG
TACGATGGATCCAGATGGCGGAGCAACTGG
GAACGTCCTGGCGCAGTTGTGTGAGCTCGT
ACGGCGTCGGAGAGAGCGGATGGTGGTCCA
CGGGGTCTTCGCTGTCGGCGCCATCGGCCA
AGCCGCCATCTTGGGCGTGCCGCGGCGGGA
TTGGCGACACCGCGTTGCGACGCCATGTTG
CGGAAGAGTCAGCGGCGGACGGAAGCAGGC
CAGTCGTCACCTTACAACTGTCAAAGAATT
GGCGATTGGAGTGCGTTCGCCTGCAGATTT
TACGTTCGGCAAGGTGGACGGACGCTGAGG
TGCTTCCGACTCTTCTTGACGATGAGTCTGT
GCTCTATAAAAGCCGCGAAGAAGAAAACA
TGTTCGACGAAATGGCGGACGCGTACGAGC
CGCCCATCGCCGATTCGTGCAGCGGCAACG
GAGTCGCCTGTGGCGTACAGGGGCGCCGTG
CTATGGCCGCTTACCCGGAGACGGAGCCTG
ACCTCTCATCTTGGGGAAGATGTTGGAGTT

CTGGGCATTCCTATGCCGGTGTGCGGTCACC
CGTCCCGGGCCGCGGCCAAGTGTCTGGACGG
TCTCCGCCGCTGGAAACAAGTCACGGCGTGC
CCTGTCGTCGATGGGGGGGCCGTAGGGTGGC
AGGCCGTGTACGTGCCGGATGCGAGGGGCCC
GTCGGCCGCCTCCGCTCCGTGGCCGGCCCGA
GGGCCGCGGGCTGCGCCGCCGGCGCGGCCGG
GGGGGCCTGCTGCGCGCCGCACCGACGTCGA
CTGCGGCCGTCGGGGCCACTACGCCAGGGAC
CGCGTTCCGGGGCCGCCCCAGCAGGAGGCCT
ACGCGCCAAAGAAAGACCCTCCGGCACAGGC
CGCACCAGACACCCGGCAAGGCCTGACACCT
CCGTTGTTGTGCGATCCGGGTGTTGTTAAAA
CAGTAACTGCGCCTTGTCGCTCGATAACGCA
GTTGGCTCGGCGGGTTGTCGCGGCCGCTACC
ACTGTACCGGGGTCTATCCAAGGGGTGGAGC
GCGTATTTTGGTGGATTCCGGGGCGTCCGCC
TCGGATGTCATTTTCCATCTTTTGAACATCC
CCGTGAGGCCGGCTTACGCTTCCTGCATCGC
GGGTAGTATGGGCATCGTGGGCCAGGTGGAC
TCTATCGGGGGTGTGTCGGCCTCGGGCGTTC
CGAGGTCCCTGGCGGTTCCTTGTTTGTTGGC
TTTGGCTAAGATGCCCATTCTAATTAGAATT
TGCATTGAACTACCGGGGGGCCGCAAAATTT
CGTCGCCCTGGGGGCGACCTGCTCCGGTCGC
GGCGCTATCCGAAGTTTCTGTTGATATTCCA
CAGATGCTCATTCCCTTGCGATATCGGAAGC
GTCGGCAGGGTGCTTTTGGGATTTTGTTGTC
CGCTATTACGGGCGGATTGGCGCTGGTGGGC
ATTGTCCGGCCGGATCAGCGCCCGTTTTTGC
ATGCGAGTACTGAAAGCGTGTGTGTGCCGGC
TGTGGCACATGCCACTACGGCGTTTACGGCT
CGGCCGGCCGGGGGGTGGTGGGTGGTGTTTC
AGGGGTGGATGATCGTTGGATCGCCGAATTA
AATTCGGCCCTTTCCTGCGGAGCAGCGTTGT
AAATTATTATCGAGCTATTCCGATGTGTTTA
ATTTGGACTTGGGACGCACCTCGCTGCTTTC
TGACACGGGGGACGCTGCCCCTATTCGGTTC
CGGATGAGTCCGGCTGAGCGTGAGCACGTTC
TGGAAGACATGTTGGCAGCGGATATTATATC
CAGCCCATGGGGCGCGCCTGTTGTGTTGGTT
GGATGGTAAATTGCGATTTTGTGTGGATTTTC
ATAAAGTGTCGGTGGCAGATGCGTATCCCCT
GGACGATTCCCTCGACGCCTTGTCGGGGGCT
TCAACGCTAGATCTCTCTTCGGGCTTCTGGC
TTGACGAGCCCTCGCGGGAAAAGACAGCTTT
GCAGGGGTTGTTTCAATTTAACTGTCTTCCC
AATGCGGCCCCTGCCACTTTTCAGAGGTTGA
TATTGACGGGGTTGCAGTGGGAGACATGTCT
AGACGATATTATCGTCTTCAGCCGCACATTT
TTACTGCGGTTGGAGGCTGTGTTCGCCAGGA
CTAATTTGAAATTCAAGCCTAAGAAATGTCA
ACGCTCCGTTCGATACTTGGGACATATTGTT
GGGGTGGCCACGGATCCGGAGAAAACTGCGT
ATTGGCCAACCCCGGCCACGGTGGGTGATGT
TTTGGGGTTCGCCTCATATTATCGGAGGTTT
TTTGCTACGATCGCGAGGCCTTTGCATCTCC
AGGGACATAAATTTGTGTGGGGACCGCCGGA
CGTTTCAGAGCCTGCGCGATCTATTGGCTACT
GTTGGATTATCCGGATTTTGAACATCCCTTCA
CGGATGCGAGTGACACCGGTATTGGGGCGGT
GTCAAAAGGTGGTGTTGAGCGCGTGGTGGCC
CGGGTTTTGTCTCCCGCGGAAACCCGCTACT
GCAGGGAACTTTTGGCTATTGTGTTTTTTCT
TCGACCGTATCTGTACGGTCGGTCGTTTATT
GACCATGCGGCCTTGCAGTATGCTCTTAAGA
CATCCGGCCAATTGGCGCGATGGTTGGACAC
ATTTTCCTTCCAAATTCACCATCGGAAGGGT
GCTAACGCGGACGCACTTTCCAGGTTGCCGC
CTTGCGCGTGTGCGTCGGGGACGCATAGGTC
TCAGCCCCCCCCTCAGCCTGTCGCGTTGGCA

CCCGAAGCTCCGGTTGAACCGGGACCGGCTC
TGCATCTTCCAAGGTCTGTGTGGATTCGGGA
GGACGATTCTTTGGTGTCGTTACGGAAACGA
GCGGGTGGGGGACTCGGTGCGGGTGGTATGC
CTCTCCGAGATGGGTTGCTGATGTGGAGGGA
GGAACACTGGCGTCTCGTTGTGCCTGCGTCA
GAGTTGATTAGAACTCTGCACTGTGACACAC
TGGGGGTGCAGAAAACCCTATTGACGCTGCA
TTATTGGCACGGCATGACGACGGATGTCGTC
CGCGCGTGTCCCGATTGTGCCCGCCGTAAGC
ATCCATATGTTCCGCCGATACAATCTATTCA
GGTTAATGAGTTGGTGGCGCTCGATATTTTC
CCGCCATCGGCTAGGGGTAATAAGTATTTGC
TGGAGTACCTCTCCCGCTGGCCGATGGCCTC
CGACCAGTCGGCGGCGTCTATCACGGACGCC
GGTATGTCATGGACAAGGGGGCGCCGGAGA
CGGACCAAGGGCGAAATTTTTCGTCTAAATT
GGTGTGCGCGGCTCTGGGAACTAAGAAGGTA
CCCTACCACCCGCAAACGGATGGTATGGTGC
ACCGGACACTGTCCGCCATGCTGTGCCATAC
GAAACAATCTGATTGGGACTTGCATATCCCT
TCGGCGTATCGGATGACGCCGCATGCGGCTA
CGCCGTTTTTTCTTATGTACGGTCGGGAAAA
GATGGATGTGCAGCTGGGAACCGGTGCGAGC
AAGCCTCTTCCTGAGTATATTCAGGACACCC
TCGCTGAGGCGCGGGAAAGTGCCCGCATGGC
AGCTCAGGCCCGGAATGAGCGGTGTGCCCGA
GGGGAGTGCCCCTGGAAAGTGGGGGACCTGC
ATTGCCCACAGGTGCGGCCAGGTACCTCTCC
TAAACCTTGGCAAGGGCCGGTCCGCCTCATT
AACCCACAGTCGGCGCGTGTTCAGGTTGGTT
GGTTCGTGCACACGTCCCGGTTGAAGCCATA
GCGGCCGGATGCGTCATCTCCCGATGTTGCC
TCTGTTGCTGCCGACTCGCCCTTGCCTGTGT
TTGCAAGAGCGCCTGTCGTACCTTTTACAGA
CGTGACACCGCAGGGGAGGAGGCGCTCTGCT
CCTACCAGAGCCGCGGAACCTACGGGACCTC
CTACCCCGCCGCGGACGCGATTGCGGGACAC
ACGGGAACGGGACGGTGGCGGGCCTCCAAT
AGAACTTGCGCCCGTCGCTCCTAAAACCCTT
CGCTCGGCGGGTGGGCCGCCGACCGTCCGGC
CGACCCGCACTCGCTCGAAATCCCTCACGTT
GTTTTGTTGTTTTGTTCTTGTTGTGTATTTA
TCTGCTACGTTATTGATTGTTGTGTTGTGTT
TACTGTTATATTGCATGCTTCGTGTCCTTTG
GTCGTTTTCGCCCGCATTACTGTTGGTGTTG
GGTCGGCTGGATTTTTCTCTACGGGGGAGGC
GCGGGCGTAAACGACCTCCCGGATGTGACGT
GAGGAGTTAGGTGACGTGGGAGGAAAGGTTA
GGGAGGAGACCGGAAGAGGAAGGGAGAGAG
GGACCAGAGTAAGGCCACGTAGGGCGAAGCC
CAGCGGCGGCCAGCGGTGGCCAGCTAAGGGA
GGAGCTGCGAGAGAAGACAGCGGTGGGGCTC
AGGCGGACGAGGAGCTGCGGGGAACACAGCC
GTGTGTGAGGGAGAGGCTACGAGAGCGACGA
GAGGCTGAGAGAACGACGAGGGGAGAAACT
AGTATCAGTGTCAGTATAGAGTCTTATAGAA
TGTGGGTTAAGAGAGAAGAACCGAATAAAGC
CAGACATCGTGGTCGAGTCCTTTATTACATA
TGTATGTAAA
>ERV-Spuma.a-Lca
GCCGCCGCCACGAAGCCGGGGGGGCTATGTC
CCACGAAGCCGTTCGGCAGGCGCCCCATCCC
CACCACCAAGCAACGGAGCCGACCCCGCGTC
CACCTCACATGAACACACCCGCACGCATTTC
TAGAGCTCCGAGGGGAGGTCACCTGGCCGAC
TCCGAGAAACCCTTATCGGGATTAACTCTCA
GCGATCAGGGAATTGGACGACCTCCCCCGTC
ACCGCGGCGCTGCGACCGGCCTTAATATGGT
GCGTGGGGCATTCGGCCACCGCGGTTGAGGC
CCACACACACCCCCACGGGTCGACGGGGCA

GGACGGGACAGCATGAATAGCGGGACCGC
GCGCATAGTCATGGAGGGTTGGGATAATTG
CGCAGGCCCCATTGTTAGGGAGGGTAAGGG
ATTTACGCCGCCAACGCCGCTCACTGATTA
AGCGCCCGGAACCGCCGTGCGCGGGCTTGA
CCCATTGTTCAGCGCAGCATCCCTTTGAGG
AAATAGAGGGGCGCAGAGGGGAAATCGCTC
AGCGCCAGGCGACCAGACGCAGCTCCAGCC
ACGTCCGTGCGACGCATCGAGGTACGCTGT
ATCAGCGAACCGCCGTGGGCACCTAACAGT
GTGAAKTAGGATCATAGCCGATAAATAGGG
GGGTTAGTAGAATAGGCCAGCCTCGTCGAC
AACATCAATTCCCAGGTAGCAGATAATAAA
CGAAGCGTGGTCTCGCGTGATCTTGTCGTG
GTGTGGGTGCGCTGTGTGCACAGTGGTTCG
GAGCGCTGCGCGGAGCTGAGCGGCGCGTGA
ATTATGTAGTGCTGCGGCTCGCCTCGGGGA
GAGCGGCGCGAGAGTGGGTGTCGCGTGTGT
TGTGATACAGGTGCGCGATCATAACGTGAG
GTGCGCGGAGCGGCGTGTGTGTGGGTGTGC
TCCGTGTGGGTTGCAGGTGCGCGATTAGGA
TCGTGGTAGAGAGGGGCATAATACTACTCG
ATGCCTACTACGCGGACCCGCGGTGGAGGG
GCGACGGCTCCCACACCATGGGTGAGGAAC
TTCGCTTAGCAGCGGGGGGCGAGTGATGAT
CCGTTAGCATTATCGAGGGAGGGATCTCGG
CCCCCACATATTGGTGTCAGAAGCGGGACG
CCCCCAAGGGAAAGAGGCCCCCCAACCCAG
AATGCACATTTACGGGCAAAGGAATTCCAT
GTTTGCCCGCAGCATCAGGGGGGCTAAAAA
TGGTCGGACGAGGAGGCGTTGCAGCAGCTA
TAGATGGGCGGGCAGCCGACTTTGTGGAAG
AAGCGCACAAACTCAGTGGGAGGAACTAAT
GAACAGCGCTTCGGCACGTATGACGAGGAG
CGGCCTTAGAGGCACGCCGCCGCCAACCCG
ACGAGATTACGCCGAGGACGTGCGCACGTT
GCGTACCCGGAGTACGAGGCGAAAGTAATT
CCGCCGGGAAGGTCCAGCGATATTTGGCAC
GTGGCAGCCAAATATGGACGGTGCACGCCT
ACCCTTGTCGAGCGCGCGCGTAAATGCGAG
GTGAGCGCGCGCAGCTTCGAGGGAGGGGAC
CGTAGCGAGCCTACTCTCCGACGAGGAGGA
AGCGCAGTAAGGGACGGGCAAGGGAGCCCC
CGAGACAACCCCGGGGTGGGTAGCCGGGTT
ATGGGAGATATGGTAACCCAAGTACGCGAG
AAAATATGAAGACCATCCAGTCCGAGATGG
CTCCACGGTAGGGGGGCTAGCCGGGAGGGT
GAGGCCGAGTTGGGACAACTGCGTACTGGG
GGAGCGGGCAGGGGGCCCACTACGGAGCCG
CGGGCATGTTATATGTGCGATCGCCGAGGG
GGGACTGTCCTCAGATCGCCCGAGCTAGAC
GTTAAACACCAACGCCCCCTAGAGAGGGGA
GTGCAATCGACGCGGTCCCGACACAAATAG
GAATAATGGCGAGCGAGTGATCTCCACACA
ACAACGCCCACGGAAACGGTGACTCCTGGC
GGGAGCTGGTGGCGGGGATCACGGGGGCCA
GGAAGGGGAGGTAAACGGAGTGCGTACCCG
GACACGGGGGCGGTGGCCTCGCTCGTGAGC
GCAGCGCGATCGGGGTCGACGTGCGTCACC
GGACCGCGCGCTGCACACGGCGAGCGGAGC
CTGCTGGGGAGAGCCACCCTAGAGGTCCGC
AGGTGTGCACCCAGGAGTTCTACGTGTCGA
TCACGAGTGTATCGCAGGTACCGACCTCCT
GGACTCAACGTGCAACCCCGCCAGCGATGC
AGAGCAACGGCGAGCGGATACCGTTCCTGG
GACGCGCCCCCGCTTCGCACCTGTGGCCGC
ACGGCAGTCACCATCGGCCCGCTCACCGAG
CGGTGGCAACATCCCCCACCGACCCCGCGC
AGGGGAGGTAATGCTAACTCCTCACCCGGA
CGGTATGGGGTTGTGGGATCCGCCACGTTG
ATGGGGACCGATTGTTTATGCGGATGTTTA

TACCCCAGCGATACTGCGACGGCGCACCCCC
GTACACCCCCTGCACGCACGCCACTCTAACC
TCGCACTCTGGGCTGAGGACCCCGGTCGTCC
CCAGGACCTGGCCTGGCGAGTCGGCCCGACT
GAGGTCGTTCCCCTGCTCGACGCGTTACATC
CGGATTTACCTAAGGGAGAGGCCGATCGGCT
ACTGCGGGAGTATGCCGACGTGTTCAGTGAA
GACGTGGGTCGGACCGACCTTGTTAAGCACC
CCGGTGCAGCCCGACCCATCAAACTCCCTGC
GGCGCACCCGAACGCGCCCAGATCAAAGAC
GACATGCTTCGCGATAACATAATTCAGCCCT
CCTGGTCAGCCCCGGTGGTACTAGTAACGAA
GTCAACCCGGTTCTGCGTCGATTACCGTAAA
GTCACGCTCGGCGACGCCTTCCCCATACCCC
ACACCTTTGACAGCCTCGCCGGAGCGCGATA
CCTCGATCTCGCGTCCGGGTACTGGCAGGTC
GAGGAGGATCGGCCGAAAACCGCATTCACCA
GACTATTCGAGTTTCGAGTACTCCCGTTCGC
CGCGCCCGCGACGTTCCAGCGGCTGATGGAC
CGGGGCTTGCAATGGGAACAGTGCCTGATTT
ATGTGATAGTGTTCAGTAGATCTTTGTCTGA
CAGGCTACGAGAGGTGTTCCAGCGACTGCGA
CTCAAGCTCAAGCCACGTAAGTGTTACATCC
AGGTGGGGTACCTCGGGCATCGCGTCAGCGA
GCGCACCGATCCGGAGAAGGTACGCGCCGTA
CCACGGCCAAGGAATCTGACGGAGATCAGAT
GGCTCGCCACCTATTATCGTCGATTTGTAAA
CGAGATTGCACGCCCCCTCACCCAACTCACC
CACCCCTATCAGTGGACGGGGGCCTGCCAGA
CCACTCTCAAAGACCACCTCACCCACGCCCC
CTTTCCCCGATTTTACCACCGATTTTATTTTG
GCCTGCAGCAGCGGGCTGGGCGCGGTGTTGT
AGGATGGGACGGAACGAGTCATCGCGTACGC
CTTGAGTGGGGCCGAGCTGAACTATTGCGTC
GAGTTGTACGCCGTGATTTTCGCATGTAAAC
CGTATTTGTACGGCAGGAGATGTCGCGTACC
ATTATGCTCTGCAGTTTCTTCTTACATTCAAC
GGGCAGATGGCCCGGTGGTTGGCGCAGTTGC
ACCTGCAAATCGAGTATCGGGCTGGCCGGAC
CGCGGACGCCTTGTCGAGGCGACCCACGGTC
ACAAGGGCGGAGGAGGCCTGCCCGTGTTTCA
GCTGCCGGCAGGCAACGGACCCCCGAGAGGC
TGACCCCCCCCAGCGAGAGAGAGTGCAGTGT
AGACCCTCACAGGTGGCACCGAGGATCCCGT
GGCAAGCGCGGGACCCGCGGCTCGGGACCCC
ATCCAGGAGGACGTCGGCTGCGCCTTCCCGC
ACGAGCAGCTGAGACAGACCCAGCAGCGAGA
GGCGGCAGTGGAGGCGGCACTGCGGGAGGAA
CTACCAGGACCCTGGAGCGGTTTGTATGCCC
TGCAGAACGGAGTGGTACGGGAGTTAACCGA
GCCGGGGTGCATCTGTGTTCCTGCCCAGGTC
CTGCTACGGCTGGTGCATGACCACCCACTCC
ATGGGGAACGACGCACTCGAGAGACCCTGCC
TCTCTGGCCCAAATTGGCACAAGACGTGGCC
CAGGGCTGCCGCGCCTGTCAGCAGTGCGGGC
CACGATACCAGGTACCCCTAAGGCCGTTCCC
CACGAATCAGCTGGTCGGGATCGACCTACAC
CCACGATCCCATCGAGGCAACCGGTATATTC
TAGAGTATTTCACCCGCTACCCCGTGGCAGT
TGATAAAACGGCCTTGGTGGTGGCCCGGGCA
CATTACGTACTTAAGCACGGGATCCCAGAAC
CCGACCAGGGTAGCGAGTTCGAGGCCGAGCT
ACTGTGCCGAGAGTTCGGAATCCAGAAGGAC
GCGTACCACCCCCAGGCGAACGGTATGGTAC
ATCAGACCCTAGCGGGGAAACTCAAACGTAT
AAATCAACGGGATTGGGACGAACACCTCCCC
TTCGCCTACGCCACCACCGTACACACGTCGA
ACCCCGTTTTTAATGATGTTCGGCCGTGAGTC
CGCAGATCTGCTATTCGGCGTCCCTTCCCCC
GAGGGAAGCCGCCCCGTGGAACACCTGTTGC
GAGAAGCCCGAGGCCGAGCATACCCGCAAGC

GCATCGGCGGCAGGAACAGGGCACCCGACCC
CCCCCCTTATTCCTGACAGGCGCTAAGGTGT
CCCCTCGCGTCGGGGCCGGGCAGGTGGGGAA
CTACTGGCGGGGACCCTGGACCGTGGTGCGC
CCCTGGACGGTACTAATTTATGGCGGGGAGA
AGACGGTGGTCAACGTCCGCCGCTTAAAACC
GGCCCCGGCGAGAGAGAACCCCGTCACCCGA
AATCCCGGAGTACCCGCACCCCTCGTGTTGC
CACACTACATGGGCCCGCCGGGGCCCCGAGA
CCAGTCCGGAGACGGCGCGGACGACGGCCCC
CCGCTGCTGAGTTTGTGCCCCAGCCCGCAGC
ACGGGAGAGCCGCTGGAGTGGAGGAGGAGAG
AGCTACAGAGGACGGAGCCACCCGCCCGAGC
AGGTCCGGACGGATCCCCCGGACCCCGCAGC
TGGGCGCGGCCCAAGCGAGGTGCTCCAGTCA
GGGGACTGGCACCTCGGGGGGCTATGTGATC
CGAAGCCGTTCGGCAGGCGCCCAATCCCCGA
CACCAAGCAACGGAGCCGACCCCGCGTCCCC
CTCACATGAACACACCCGCACGCATTTCCCC
AGCTGGGAGGGGAGGTCACCTGACCGACCGC
GAGAGACCCTTATCGGGATTAATTCTCACGC
ATCAGGGAATTGGATGACCTCCCCCGTGCTA
GCGGCGCTGCGGCCGGCCTTAATATGGTGTC
TGGGGCATTCGGCCACCGCGGTTGAGGCGCA
CACACACCCCCACGGGTCGACGGGGCAAGCA
GGGACAGCATGAATAGCGGGGTCACAGGGGG
TAGTCATGGAGGGTTGGGATAATTGCGCTTC
GCCCCATTGTTAGGAAGGGTAAGGGATTAAT
CGCCGCGCACGCCGCACACTGATTAGCATAT
CCGGAACCGCCGTGCGCGGGCTTGGGGTGCA
TGTTCAGCGCAGCATCCCCTTTGAGGTAGGC
AGAGGAGCGCAGAGGGGAAATCGCTCTCTTC
CCAGGCGACCAGACGCAGCTCCAGCCAGCGA
CCGTGCGACGCATCGAGGTACGCTGTTAGCA
GCGAACCGCCGTGGGCACCTAACAGTGAGTA
ATTAGGTATCATAAGCCGATAAATAGGGAAT
TTTAGTGGAATAGGCCAGCCTCGTCGACTCA
CATCAATTCGCAGGTAGCAGATAATAAATCA
AAGCGTGGTCTCGCGTGATCTTGTCGTGCAC
GTGGGTGCGCTGTGTGCACAGTGGTTCGCCA
GCGCTGCGCGGAGCTGAGCGCGCGGGTTGCC
GCAGTGGTTCTGTAGTGCTGCGGCTCGCCTC
CTGCGCAGAGCGGCGCGAGAGTGGGTGTCGG
CTCCGTGTGTGATACAGGTGCGCGATCATAA
AGTTGGAGTGCGCGGAGCGGCGTGTGTGTGC
CGTTCGCTACGTGTGGTTGCAGGCGCGCGAT
GAAGTATCGTAGTAGAGAGGGGCAGAATAC
TAACCATGCCTATTACGCGGACCCGCGGTGC
TCGGGCGACGGCTCCCACACCATGGGTGAGC
GCGGTTCGCTTAGCAGCGGGGGGCGATTGAT
CCGGCCGTTAGCATTATCGAGGGAGGGATCT
GGGGCTAGCGACCCCCACATCTM
>ERV-Epcilon.0-Ebu
AGCTGTTGAACATCTCCCAACACTGAAAAAC
TGGTGAAAATACTTTAAACGRTCGAAAGTAA
RTATAAAAAGGTGTTGTTTTCATTTATTGAT
CAGCKGAACATCTGGCAAGCAGAATGAGAAC
ATTGTGATAAAGATCTGCCTGTCACATTAAC
GATCTTGCTGATGAAAGATCAATGCTTTGCCA
GAGAAAACAGTACTGGGGGGAGTTTTCTTTT
AAGGAAAATGATCTCTGAGGATGGCATCTCA
CTGATGGAAGTGGGAAATCATGAAAACAGAT
AGGGGGCAGGACAAATTGTATATAAAGCAAC
TAAGCGAGCCAGAGTTAGATTGAGCTGGACT
CATTCTGTGTCTCTCTTTCTCTCGTGTATACA
GTGAATTAAAGCTCTTCTCTACCACGATCAC
TGCTTGATCTCAATCAGCCGTGAAACCTGAT
TGGCGTAGTCGGCAAGGGATAAGCAGACCAA
ACWGCTTCCTTGGTGAGACAAAAGAGGAAAC
GCTTAAAGATAAGGTAAGAAACTGCCTCTTT
AGGGCATACAGGTTCGCTGCTCTTTGTGATC

CTTAAAAGAACGGGGAAGAGGGAGCTTTTG
AAGAGGGGTACCCCGGGAGGTTTAGGGTCC
CGAAGGGGAGGTTGAGGGAAAACGCTGGAA
AGAAATCTCCGGAGGACAGGGTCCAAAGGG
GGAAAAACCCTGGGAGGGTAACCTGAGACA
ACAAGGTTTTTGTGGGAGATTGAGGGCGTG
AAGGGAGTGTGAGGGTCCGTGGAAAGGTGT
CGACCCAGCCCGAGAACACCCCGGGCAAAA
CCCAAGAATACTCTAGGAAGGATCAAGGAA
AAAACGTCGAAACCAAAGTTGGATAAAAAT
CGAATACCGTGGCGGGAATGATGTATGAAA
TAAGTCATGTACTCAATTGGCATATGGTAT
AGGAGGACCCATACCCATTCCCGAGATGGG
AGCAGAAATTATAGATAACTTGTCATGTCG
CGGACAAGTGATAAGTTAGAACCAGAGCCA
CTTGTAAAATGTGGGAAGAAGAAGCCCTAC
AGTGGCTAGAAATTTGGTAAAACGGAAACT
GAAACACAGGTTAAAGGATTGCAACAACCA
TTACTCCCGCCCCTGTAACTGCCTCTGAAA
AGGGCGGGAAGCTCAGGGAAGTAATGCAGG
GCATATGTGAATAACACACCAAGTTTGTAT
AAGGTATGGGGCTGCTGGCCACAGCCTCTG
TCAAGAAATAACTCCGGCGGGACCAGGAGT
TCAGTGTACCGACCGTTTAAAGAAAGCGAA
TGAAGGAAAGAGTGTTAGATCTGAGAGGAA
AATAAAGGAGTTAATAACGCAGTGGGTTGA
TGCACGCATAGCGACATTGGGGTGTTATGT
TGTCCCCATATGTGTGGAAGAAAGCTAGGG
ATGGACAGCGGACGAACCAGCAGGATGCAC
CGGCAAGATAAGCGACGGCGCCTGTATGAT
AAGCGTTGCGACCGGCAGCACTAGATTGGG
TGAAACAAAGCAACGTCAAGGGGAACGGGC
ATGGATAGATTAGTGCAGACATTCTTACAA
TTCAAGTTGAAGAAGACATACCGCAGCGAA
CTTCGTAATGGAAGGGCTCAGGCCTCAAAT
TTAAAGTATGTAATGCTAGGTTGGCAGGGC
AGTGAGATTTGTATAGCGGCTGATCAATGTT
GGAAGAAAGGGAGAGCGCAAAGACAAAAAT
AGTGTTGCAGATGGTGTTGTTAGACAAAGTA
GAAAGAATAAGGATTGGAGAAAGTGGGAAC
TGACACAGACTCGGAAGAAGAAGCGGCCAG
CGGCGAATGAGAGTTCCGCAAAGACCCCCA
AGGATGGACGGCCCAAGAAATGGAGTGGAA
CTGGACACGAACGCCACCACAGACCCGCTC
GCAGGCCAAGGGCAGGTACAACCAACTGCG
GTTTCCAGTGCGGGCAACTAGGACATTGGA
CCCCCAAAGAGGACGATCGGGGTTCCAGCA
TCGAGACAACCGCAGGATCGTAAGTCATAT
CGGGTGGACCCGTGTTGCAAGGAGCAATGC
ACCAACAGAGGAGCCGACCTACACTCTGGA
AAGAAGCTTTCCTTTTTAGTTGACACGGGA
CTACTATTGCAGCCAAAGAACTGCCAGGGG
CAATACTAATGTACAAGTAGTAGGGTTAGG
CAGACATTAAACACAACAACACAATTACCA
GACCTTTGCAGGAAAAACATGCATTCCTTC
TACACCGGTGAATTTGTTAGGTAGAGATTT
TTGGGATGTACGATAGCATGTACAAAAAAA
TTGATATTCCCCGGGATAAAACACATTCAG
TTTGATGACACCGCACCCAGTTGTCTATTG
CCAAGACAGCCGATTAGTGATGTTTTGATA
CAATCTTAGCCGAAAGTCAGGAAATGCAAA
CAATGCACAAGTAATGACACACACCTACTA
CATTGTACAATCTATTATGATGCGAATGGA
AATTTGCGGAGAAAATTGCTCCGCGTTTGG
ATGATTTATTGGTTTGCCGTTTAATGATTGG
GTTGCAATGGAGGTTAAGCTCACAGAAGAA
TGACAAAGTACCCGCCGGGAGTAACCTCGC
AACCACAGCAAAGGGATATCACCCAAAGGA
ATGGTTTATCGCCTGCAGGGGGCAGTAGTG
CAACAATGTTGCATTGGCAAGTGCATAAGT
CGAAGGGTGGATTTGTGATTTTAAGGAACA

GGATGGTGGAGGAGGTTAATCTACCACCTA
TGGTGATGGAAACGGCAAGTGAAACAGGAA
TTGATAGAAAAGGTTCCACAAGAATTGTGGA
ATAATGATGTGGGTCGTATCATTGGGGCAAC
GGTTAAACTCAAACCTGGAATGCGTTGGCCC
CAATACCCTCTCCGAAAGGAGGCAGAGGCCC
CAGTAATTGAATCTTTGAAGGAACAAGGTAT
AACTTCAAGTCCATGTAACACACCTATATTC
AAGCCACAGAAGAATAAGTGGAGATTTGTCC
GAGAAGTAAATGAGGTGATTCAAGGTGTTCA
ACCTAGTGCAGAGGGGTTGATAACATCTATC
CTCCACCCATTTTACTGTAATTGATTTATGTT
TCTCCGTACCCATTGATAAAGAATCGCAGTT
CTTTACGTATAAAGGCCGTCAATATACATGC
CCGCAGGGGTTTGCTGATAGCCCTACATTGT
CTTTGCATAAGGACTTAGAAGATGTTGTCTT
ATCAGTGCTGCTACAATATGTGGATGACTTC
TCCCCAAACAAAGCAGCGTGTGAAACAGATA
TGCTACAGGCATTGGCAAGGAAAGGCCACAA
AGACAAGCTGCAATTAGTGCAGGAAAGGGT
GGACACCACATTTCACACGGAAAGAGGGAAT
GGAGGATAACTTCAATTGCGGAAGTGCCCCC
AAAGAAAGAGATGATGCGATTCCTAGGTATC
TGTAGACAGTGGGTTGTCGATTATGCAATTC
AACTGTATGTTGTGGTGGGCAAAAACCAGCC
CGAGTGGACTGAGAAGATGGGGGAATGTTT
AAAGCAGCACTCTCGACGGCCCCAGCTTTGC
ATTATGAGAAACCGTTCTTCCTTTATTCTCA
AGGATTTGCGCAGGCTGTGCTGACACAATCC
CGCCAGCGACCAATTGCATATTTCAGCCGAA
CGGTCGAACGCGGCCTACCCCTTTGTTTGCA
CGCAGCCAGCTATGCAGTTAAGCGCACGGCA
ATGGGTCACCCTCTTACTCTAAGGGTACCCC
CGGCTTTATTAACCCAAAAGCAAACACAACA
CACACGCCTGATGCAGTATGAGACGACTCTC
CCGAACCTGCAGATTGAGAGATGTAACGTTC
CTTCCTTGCTGCCAACGGAAGAAGACGGCGA
TTGCACAGAGGCGATCCAGGAAGTATCCACC
GATCTAAAGGATCAACCCTTAGAACACCCGC
TGTTTGTTGATGGTTCGGCCTATCGAGGCCA
GGGGCATGCAGGATACGCCGTGTGCACCGAT
TATTGAAGCGATACGCCTGCCTTCACATTTTT
AGGCGGAGCTGTGTGCCCTTATAAGGGCATC
TGCCGATATCGCGGTAAATATATACACTGAC
GCTTTTGGTGTAGTACATGACAAAGGTGCGA
TGCGAGGGTTTCTCACCTCTGCAGGAACACC
TGGTGGTTTAGTTTCTGAGCTGTTAAAAAGC
CCAGCGGAAGTTGCGGTTATTAAATGCCGCC
AACTGACTACGGATATAGACAAAGGAAATGA
TAAAGCTGCAAAAGAGGCGGCTGGCCTGCCT
GTAATGCCAGCACGACTACCAAAGGAACAAA
ATCAGTATAGCTTGTCAGATATTATTGCAAT
AGCATCGCTTAATGATCATGACAAATGGCAA
GGAACAATGGATGAAGACGGACTATGGTCAA
AGGTCATAGCGCCTGCTGAGATCCTACCGTC
CATGGCCCATTTGAATGGACATGTGTGCAAC
GTGAGACAGGTACAAGAATCATGGTATGCAC
GGAAAATAGCAGAAGGGGTTTGTAAAAGCTC
TCAACAGCACAACCAACAGAAAGAGGGAAAA
CTGGGAGCTCACCCAGCCCCTTGGGGTCCGT
TACAAATAGACTTCATAAAAATGCCTCCATC
CAGCAATGTTTTGGTAGTGGTAGACATGTTC
GTAGAGGCATACCCATGTGCAAAGGCTGATC
TGGTAAAGCACCTGTTAAAAAGCTTTTTCTC
CATCCCTCATAAAGTATCACACGATCAAGGC
ACGGGGGAAGTGACACAGGCAGCATGTAAAC
TTAAACAAGCATTTCACTGTGTTTATCACCC
AGGTGCTGTAGAAAGGCAGAACGGAATCCTA
TTGGCAAAAATAACAACGGAAACGGGGTTA
ATGCATTACCCTTGGCACTAATGAGCATGCC
AAACCGGAAAACAGGGTTATCCCCTCACGAA

GGAAGGCCGATGCGGCTGCCCGTGACTGCCC
TGAATAAGCTGGATATTGCAATGATGGACGA
GACATTTTGTAAACAGCTTTCCAGGATCGCA
CACACAAGGGTAAAAGAGGCCCTACCCGAAC
AGCCGTACCACACCTTCCAGCCGGGAGACTA
AAAACACTTCACGAGGAAAACAGCCTTGGAA
AAAGGACCATTTCAGGTACTGCTGACCACCC
TAAAGTGTGAAGGTAAGACAGCTTGGATACA
CTGTAAACTTGCAATACCAGCAGAGACAGGC
ACAACACCTATCTTACCATGACCGCGATTCT
TGTTTGCACGTGGGTAATTCACAATACCGAC
GATAATGAAGGAAACACTGATGATGTGAGAA
TGGGAAAAGGGAGTAAGAGATTAGATCAGGT
GGGTGGTAAAAGAAAGAGACGAGAAATAAC
GAATTGACCGATCATCCACTGAACACCTACC
CTCTACAGTACGCTAGGGCGATGAACAAAAA
GGTATGTTCACACCTCCCACACTCCTCAAAA
CCCTTGTTTGCAGTTCCACTCCAGGGGGAGC
GGTACTTAGTCCGTAGACAGGATAGACCAGT
AGTAGAGAAGACACCAGCATCAGTGAAAGGA
GAAATAAGGAGTAATTGGGAAAAGGGGGA
CGAACTATGAGCATTGGTATTACCCTAAGTA
TAAGCGGAAAGTCGTGATACAGGGCCCGGCC
AAAATAACAAACTATGACGGATTGAAGAAGC
TTAGAAAAGTACCCTCCACATTTTCAACTCAT
AATTGGACTAATTTGCCTAGTTAGAAGACAC
CCACTTAGGAGAATCAACATGCCAATATTAT
GGTAATGAGACGGTTACCATTAAAGGACGAA
CAACCATACCTGTAAAAATAGAAGCAAATAA
ACCTCAGGCACCCCTGAATGGTACATATTTT
TATAAGGCATACTCATGGCTACCAGTGGGAT
TCATGCTATTTGGGATTTGTACTACCCAGGAT
TGACCAATCACCATTTATGCCACAGGCCAGA
AGCCATTTCAGAAGTTGAGAGGTTTTTCATGA
CTTTTTATGGAGCTGGCAAAAGTGCAAGGGA
TTTGGCAGCATCATTGGAGAAACTTGCTAAC
GAAGGATTTCACAAAACAAGTGCCGAGCTGC
GGACGACCGTTATGCAAAACCGCATCGCTCT
GTTAGCAGAAAAGGGAGGGACTTGTTCGGTC
GAATGCTGCACATACATTCCAGATAATTCAC
ATAATTTGGCTAACAAAATTAAAGCTGAAGC
TGATTTTAACAAGGGATGGTCAATGGGATCT
GGAATATTTGGTAGTTGGGGAGCCTGGATAA
TGTTCATAGGGCTCATGATCTTCATAGCTGT
CATCGCTTGTATTAAAAATCTAATCAGGGAC
CGATGTTCCTCCGCAGCAAAGAAGACAACAA
ACGGTCCTGGGGAAGCCTGCTGTGATTGCTGT
GCTGCCAACACCTCCACCGTCTCTACCCTCC
CACTCCTCTTTTTCTCCCCAACCCATAGAAC
CTGTGAACTTATGGGACTGTTGACCCACTGC
CATTGGATGAGTGTCTCGAATTGCTTGCGCC
GATGGGAAGAAATACTATCTAAACATTGCCT
AATATTGGGATTTTGTACTTTCTTATTTTTAC
CAGTTAGAAAAAAATATATTCTTGTTTGTTC
AACAATAGTTAGCCAAAATATATTCTTACTT
AAAGAACACTAGTTAGTTAAAAATATATTTA
TGTGTAAAATAACTTTGTGTGAGTCGTTTTT
ACTTTGCTTCCTCATGACCTATGCTTAATTCA
AAATGTATCCACTATTCTACAGAGGGGTGGC
GGCCGGTTAGGATGGTAGAGAGGGGGCTTTT
TAGAAGGTGCAGGCGGAGCCTCCGTGACGTC
TTGGAGCTGACCATAAGAGTGGACCACCTGT
CTCCTGCCTGCCATCCTGCCGATCCATGGATA
TCAAAATTATTGTGTAACTGTTGTGTTTCTT
GTTTCAAAGTAACAGAGGAGCAATTGTGGAC
ATCTCCCAACACTGAAAAACAGCCATTATTC
CTTTAAACAATCGAAAGTAACCTTTTAAAGT
GTGTTGTGTTCATTCTATTGATTCATCTTYAA
TCTGGCTAGCAGAATGAGAAGATAATATATA
AGATCTGCCTGTCACATTAACCAAAAACAGA
TAGTGAAAGATCAATGCTTTGCCAGTCCAGC

CAGTGCGGGGGTGANNTTNNTTTGTTACCTC
AGATTTCTGAGGATGGCATATCAAATAGAA
ATGGGAAATCATGAAAACAGATGGCACCACA
GAACAAAGTATATATATATAAAGCAAACCA
GAGCCAGAGTTTGACTGAGCTGGGATACTG
GTGYCTCACTTACTCTCGTGTATACACTTTT
TTAAAAGCTCTTCTCTACCAYGATCAGTGTA
TGATTTCAATCAGCCGTGAAAACCTGATCTC
GACACGGC
>ERV-Spuma.a-Ebu%20
CTGTATCACGTGATTGGGTTTTCGTATATCA
CGCTAGGGGTGGCAGGAATGTACATCCGGAC
AGCGGAGTTGATGCTACCCGCTCTTGCTTCT
CTGCACCTCGTACACCACAGTTGGCTGACGA
AAGGTCGAGTCACGGCGCACGGACATGTGCC
CGGAGACGACTTCGAATTGTTTTCGGAACAA
TATTTCCACGCGAATGATGTGGTCGACGAGA
AGTCGATCTTGCTCGCCTCGCTATCGACGGA
ACTGCTGAGTGATTTGGTGGCGCCGGACAAC
GACGGGCTGTCGTTTCGGGACATTGTGGAGA
CACACATGGCGCCGGAGAAGTCATTACAGCT
CCAGTTCGATCGCCTCGCGAAAGGTGCAGAC
GCTGATTTCGTGGCGCGGGTGAAGCATTCTT
GCAAGTTCACGGCGGAGGAGCGTCCAGCTCC
TCGTTTTATTTCTGGTTTACAAGATGCGAAC
GCGGTTCTACGGCTGAAGGGTGAAGCCATTA
CGGCGGTCGACACTGCTACAGCTGTCGAGCA
AGATGTTCGGGCCATCACCGGCCACGGCATC
CCGGGGGTCCACGCGGTGTCGCGAGCGCCCC
GCACGACCTTCGGTGCGGGTCGGGGCCGACC
GGCCGCACGAGCGGCGAGGTGACCCCGCGGA
GATGCGGAGGTCGACACCTGCGCCGTGTGTC
AAACACCGTGTGTTTACATTGTCACAAGGTC
AAGCGTGTGTGCCGAGGGGCGTTGGCCACCC
TGGACGGCGATGTGCGAGGCGACCCCGGCGA
GAACCGGCAGCCGTTGATGAGCCTGTTTTCA
GCGGCGGCGCGCCCGATGACGGTGGAAGTCC
AAACGCCGATCACGTTTCAGGTTGATACGGC
GTCGATCATCGGCGAACAGACCGCGCGTAGA
CTGAAGCTTATCAGGTCAAACCTCACGCTGT
CCCGCGAGAAGATAACGCCAGTCGGGATGGC
TGTGCGGTACCAAGGCCAGGTCGCCGCGCTT
GTCGTGCGGGGGAGGGGGCCGTCTCTCCTCC
GGCTGACCCGCATCAGGCTAGACTGGGCCGC
TGTGCAACCCGTGCCGAGCCGATGGGAGGAT
AAGATTCTTCAAAGACATCAAGGTTTGTTTT
TTGGGAAACTCAAGGGCGTGGAGGCAAAGCC
GGGGGGGGGGGGGGGGGGGGGGGGGGGGGG
GGGGGGGGGGGGGGGGGGGGGGGGGGGGGG
GGGGGGGGGGGGGGGGGGGGGGGGGGGGGG
GGGGGGGGGGGGGGGGGGGGGGGGGGGGGG
TCCGATTGTGCGGGGACTTCAAGTCCACGGT
GCTCAGCGTGGAGCAATATCCACTGCCGTCA
ATATATGCACGATTGGCGGGGAGTCAATACT
TTGATCTGCGTGATGCATATTTTCACCTGGC
CGGGAGTAGCGACCTTTTGACAATCAACACC
CTCTATCGATATACCCGACTCTGTTTTGGGA
CCCCAGCCTTGTGGCAGAAAGCCATGGATCA
TGGCCTGCCCGCTCAAACCTACCTGGACGAT
GCGGGGAAAACGGCGGACGAGCACCTTGCGA
AAGTGCTGACGAAGCTAGAAGCGTGCGGCCT
CAGGGACAAGTGCGCGTTCTTCAAGCAGCGC
CTCGGGCACATCATCACGCCGGAGGGGCTGT
CTCGGAAGGTGGAGGCGATTCAGCACATGCC
CGACAAGACGCAGCTGCGCTCGTTCATTGGT
TATTACCAAAGGTTCGTGCGCGGGCTCAGCT
CGCCGTTAACGCGGTTGCTCAGGAAGGACGA
CTGGGGGCCTGCACACCAGGCGTCGTTCGAC
CGGCTGCTCACAGAGGATACGGTCCTCGTGC
CTGCGCGTCCGCTCTTATTGGCATGCGACAC
TGGCATCGGGGCAGTGCTCTCCCACAGGATC

TCCGAGCGGCCTGTTTGTTTTGCATCCCGGA
CAGCAGAGCGGGAATATGCCCAGATCGAGCC
CAGCCTCGTCTGGGGTGTGCGGAGGTTTCAT
CTGGGGCGGCATTTCACTTTGCAGACGGATC
TTCAATTCATCCTGAGACCCGAACGCGGGGT
AGCGGCCGCGCGAATACAGCGGTGGTGTCTC
GCTTACAACTACGATATTCAGTTCCGGAGCA
ATGCTAACTGCGACGGGCTCTCGCGCTTGCC
GGGTCCAGCTTCGTCTGAGTCGGCCGACCGC
TTCGCACTGGGCCAGGTCCAAAGCGGGCCGA
AACAGGTCGCGGTGGAATCGGCTCATGATCC
CCGTGTCGTTCAGGCGATTTCTACGGGGAAT
TCTACCTTCAACACCGGCATTTCAGCCTTTCC
CATCGGAGCTGTCGTTGGTCCAACGGTGCGT
AATGAGGGCGGTGATTCCACCGAAGTTCAGC
TTGGAAGAACTGCATGTCAGTCACCTTGGCC
CCAAGAGTCTTGCTCGTAGCCTGTTGTGGTC
GGACAAAGACATTGAGCAGTTGGTTAAGCGT
TGTCAAACAGATGCTGATAACCCGCGTCGAC
TATGGCCTACCCCAGACAGGGCCTGGCAGAC
GGATTTTGCGGGGCCGGTTAATGGCAAAATC
GTGATCGATGCATTCTCTAAATGGCCGGAGC
TGCAGCATACATCAGCAAACATTACAGTAGA
GACGATCTTTGCTAATAAAGGATTACCCGAC
TCTGATAACGGTCCGCAGTTCATCGCAGGTC
AGTTTATGCGTCAAAATGGTATTCGACATCT
ACCGTATCATCCTGCGACGAATGGGCAGGCT
GTTAAGATGTTGAAACGGGCTTTGCGCAGGC
CACCTGTGCAGACGGTGGTCGATGTCTTTTT
CAGGAACGCTATTCATCCTGTCACGGGTGAT
ATTCGTCTCTATGGACGGGCATTGCGGTCCA
TGCTGCGACCTGGAGGGTCGGTGAGTGGGGC
CCCGACGCTGCTGCGCGGTTTTGATGTGGGA
ATGGTGCGCGATTATTTCCACGGTTCCAAAT
CTGTGGTCTCTGTGGTACTGGGGCCGGCGAA
GCGGACTGATGGTGGGGAGGTGTGGAAGCGC
CAAATGCATGATCGCTGTTCAGCGTCACCCC
GTGCCGAGCGATGTGGGACCCCCACCTGTAA
GGCCAGACGTTGTATCGCCAGGGCCGGATGT
CACGGGGTGTTCACCTGATTCAGTCCTGCCC
GAGCAAGCTTGTCGGCCGGGGGCGGTTGTTC
GGGCAAGTCCGGCGAGGGTGTCACCTCGACC
GGCGTCTGAGCCTGGGCCGTCTGGCGGCGCT
AGGCGGGTCAGGATGCGGCCGCGGTACTTGC
AGTGTTGATTGGTTTCGGTTGGTCATGTTAT
TTCATCATTTGACTTGTCATTTTACGAGGGC
GCTGTATCACGTGATTGGGTTTTCGTATATCA
CGCTAGGGGTGGCAGGAATGTACATCCGGAC
AGCGGAGTTGCTGCTACGCTCTCGCTCTCCA
CACTGCACCTCGGACACCACAATTTC

>ERV-Spuma.0-Lre

CTAACCCTTCTTCCAAAATTGAGATTAATAA
GATATTTTTATATATTATCTTTTTACTTGTTT
ATGAAACACACCAAAGCGCAGCAAACTGCA
GGGTTGTTAAGTAATGAACTGGAGGAGGGCT
GAATTTGCAACCTTGCTTCAAGGAAAGATAA
AACGGGCACATGTCACCCGTGCAGGCGGCAC
GAGTGTCAGCCAGACATAGTTAAGGCAAAAA
TCCTGCATGGTTTTAAAGTAACGTCAAAATT
ACAACAGAGTAGGCTAAATTATCGATCCTCC
AAATTGGAGACCAGGCTGCGAGCTACATCAA
GCCCATTTGCTAATCCAATACTGAATGTTAA
AGTCAAAACGAATTCTATGACCTCAGCACAT
ATAGGGCAGTGTCGACTCAAAGGCAATGGAA
GGCGCCTTTGTCTAAATCACAACCACAGAAA
CATCACTGATTGTGTGTTTAGGGATTCCGAC
ACACGTGCACACAGGGGGGAGGTAGAAGTAC
CCTGCAGCTGAGGGAGCAAAGATAAACCCA
AGGAATTAAATTATTAAATCCAGGGTGAGTC
AGTGGGTCAGCTCAAGGCTGTGTGCACAGGC
GATTTAAACAACCCTGCACGTCTGAACGTGA

TGACCTGAAATTAAGAATAACACTCCCAGAC
GGATGGCCACAGTGAGACGAGAATAAGAGTC
TCTCAAGGATGCAGACCCAGCTGAACAACAA
GGTCACCAGAAGTTCGCGTTCGCCTAGTTAA
GCTCAATTATTCTGTATTGCTATTAAAGTAAC
GGAATTTAACAACAGGGTAGGCTAAAGTTAT
CACCCACTCCAATTAAACCAGGCCACAAGTT
GAACCATCACCATCATTAAGTCAATATGAAA
CATACTTTAATACTTTCTATACAACTCGACC
TGACAATCAGAACCTTCCTAAGTCAAAATTC
CAGACAGGAGCGAATAGTCGATATAAGGTTC
GGAAATTAAGAGCACGGCGAACTGCAAACG
GCCGGGAACGACACCAGCCCAGCAACAGCGA
CGACAGGCCATAAAAGTAGAAATAATAACTC
GGGACACAAGAAGAAGCAGGCAGACAAAA
ACTCCAAGGTTCAACCGCCCTCACCTACAAC
AAATTAATGAGGCCTTGAGTTAACGGCGTCA
CAACAACAACGGCGAGCAGAGATAGGGTGGC
GCGGGGCTATGGAATGCAACTTCGTGTCAGT
ACAAACAAGTTAAGTTAAAGACATCACAATA
GTACATAACATCAATGTTAACGCTATTCACC
ATTCCGCTTATATAACCATGCAGACACCAAC
TTCCTAGGATCAAGAAACTTACCCCTTAACC
GGAAATAAATTAATAATAATAAGCATATGCA
AAAAGTAAAACTTATGGAAAAGCCAGGATA
TCACCACCAGGTCGAATATAAATACATAATA
CGATGTTAAACCCAGGGTCGGAGAGAAAGGC
CCGTCTTTGTATCCCCGCAGCGCGGGATAGC
ACGCTCGGGGAAACACCTCCACTTGAGTCAA
TGGGAAACGACAGACCTTAGGTTACATACAC
TGTGAAATAGTAAGGGACAAATGTTAACTTA
AACCATTTCTTTGTGCGCCGGGTCGGAGAGA
CTCCCTCCAGGTTGTGCAACACGAACAAAGA
GACGATTTGTAACGTCTGTGTTTTGGGTTCA
CCAGACAAGAGATAATATTGCACCCAGACAC
CTTCGAGGCAATTTGTAACATCTGATTGTTTC
TTTATGGAGGGAGTGTGACAACGGAATATAC
TGCACGCAAGGACATACGGACGTACGGACAC
CACACCTGCCTACGGACAGATCCTGACACAC
AAAGAAGCCCCGCCTTGTGGGCGGGATCCAC
TCGGCATAAAGGAAAGGATCCTGAGCATTGC
CGACCAGCGGGTCGACTCAGGAGCGAGCTCC
GTCGGCTCAGGAGCGAGCTGCGACCAGCGGC
CTCATCTTCAGCTCGTCTGGGACTCAGCCGA
GCTTACCAAAGCCGGCTCTCGGGGCTCGACT
CCCCATCTCCTCTGCAGGCGGGCAAGACCCC
TCAACTCGGCCTGCGTGGGTCAACCTCTGCC
GTCTCTGCTGCCGCCGGAGTGGTGAGGGTCC
GACGAGGCTCCGTGCACACCCTGGTCGACGA
GCACGCTGCGAGCGAGTTGTGGGCGCCACCT
GTTCCGAACTGCGACAGCGTATCAACGGGCA
GTTCACCACGGTGACAGGGGAGCGACTGATA
CGCACCGCCCCCTCACCACACGACCACCAGC
GACATAAAGAATCGACTCCGAACACTGACTA
ACTCTTACCACTCAAACACACTCGAACCCAT
GTGGCTGGGAAGGCATCACAACGAGACCGAC
TCCGTCGTTTATAAAAGTATTCGTAATTTTT
ATTTAGTGGGCTGTACTCTTAATCGCATAAA
GATCCATCAATACGAACTGATTCTTGGCACC
CGAGCCACGCTCACGTGGGGAGTGGTGGCCC
TTATCAGGTGATCTGGTCCCCAATTAGTAAA
AGATAACGATCTTCGGAAAAGGAGCACCGTC
TACTCCGAGCCGTTCTGTTGTCAGATAACCT
TCTGTTGAGATTGGTGCCTTTAAGAATAATA
GATTGTGTTTTTTTATCGCCGTATAATCTTT
TTCAGTCATAATCGCCGCACGTACTCGCATC
ATATTCAACTGTCCCGTGACCCAAGGTGCTA
TAAGGGTTGCGTATGATCAATAAGGAGTGCC
GAACAATATTGAGTATAATCATTCATTAACC
TCTTAGCATAGTATTTGCATATTTTAACGAC
CACTAACAACCATCGCGTTATGCGGGATCCA

GTCAAAGTGAAATCAGACAATCGTTGATGAT
ATGCCGTGTTGACGTTCCGGGATTGAGAGTT
TATCATAGTTAAGGTTGAATTAGTAATAAAT
CAGAAGTGGTTGTGTGATTCTTTCCTGAAAT
AGATCTGCTTCTGCGTCGAGATCTGAACTTC
ATAAACACACTAATACATACTCAGTAAATGT
AACGGGGTGAACCGGGGACCTAAAATATAAC
ACACTCTGGGAATGTTAAAGCGAGAATGAGA
ACAACGATGCCGAGTTAATCTAAGTTAATCT
ACACCACACCTGCATAACTGCATACGATTAA
TCCGATCGATAACCCACCCTCGTCACCGAGC
GTTATCATAACGACTTGCATTATCATAATTA
GCATAATTAATACGATTACCTCTCGAATTGC
AATGTGCATAATTACATAATTAACATATTTC
ACACATTTGGCCAAATTTCATTTAATTATTA
CTGAATATTCATAACCCACATATACAACATA
CCCGCGCACGCATACAACATAACAAATTTTC
ATATACAACAAAAACGCGCTCATAAKGGCAC
AGCAGAGTATAACAAAGACAGCTGGGCTGAA
TCGAGTTTAACTGTCCGGGATCTTGTGMAAA
TATGTACTCAGGAAGGAATCTCTAGTGTGTC
ATAGGAAATTAAAGTTGCAATATACTTTTTA
AACCACACTAAATAATWGTGATGGCACAGGC
TTCTGAGATTAACACCACMACGCATAACACT
TGGACCTCTGAGGAGGAGATGGAATCTATGC
TATCGGGAATTTGTATTAAAACGTACTGGACC
CCAGCAGACGGAGCAGAGAGATATATGGGAC
ACAGAAGATGTTAAGGCCCATAGAAGGAAAC
ACGTGATGAAATGTGGCAAAGCCAAACGTGA
TGCAGAAATAGCGAGGGTAGACTCGTATCTC
ACAGACAGAGAAAGATTATGGAATGAGGAAA
GGAGAGAAAAGAGAAACTGGCCTTAATCGT
GCGTCTGCACGCCCGCGTGAGAGTRAAAGAA
AATGCACCAGTGAAACACCCAAASGTTAATA
CATAGGGGAGACACAATTCACCACACWCMGA
ACGTAGCCCGGGGCGAGAWCACAYAATGAGT
ATGCAAAGCAATTTTGATAAAGTAACACCCT
AGGAGAGAGAAGAGGAAATAATAATACAAAC
TGAGGCCGACCACCCCAACGTTAGAGAGAGA
AACCCTAAGTCATCAGGATACACTGTAATGA
AAAGGGCGGAGCTGGTGCAAGACGGGAAACA
CAGAACACCAGGCAGACGCACGCTACGCAAC
CATGATGCAGATGACAGAGACAGCTCACCAC
CCTCGCACAAACACGACCAACACGACACACC
CTCACCAGAGAGACGCAAATCACACAGGCAC
AATAAGCGGAAATCACGGAGCAAAATTGACC
GGGCAAAAGGGCACTACAAAAGTAATGTTAA
TCGTGATGGTGACGGCTCATCTGACACTGAC
AATGACCAACACGACACGGATAACAGGAGCT
GACGCACATCACACAAGCATGAGCCATGGGA
ATCACCGAGCCAAGTTGACCCACGTAACAGC
CACTACAAAAGTTATGTTAAGCAGAGCCACC
ACGGCTCATCTGACACTGACTCCTCTGACAA
CGACATGGGTACCAGAGAGAGCTCACCAGAC
ACATGTAAAAATCACCCACGCAAAACCACCC
ATCACAGAGACAGGAATCCCGATAACCATGA
CAAACATAAATTCAACACCTCACCTAGCAGC
CGTAGTAGGCAGCATGAAGGTCGAGGACAAA
CACATAGACACCGCTCAAGGAGATCACGCAC
CAAGCACGAATCACAAGACCCACATAAGGAA
CACAAGCAACGGGACAGATACGAATCCTCGC
GATCACGACACAGACACGGGACACATGACAA
TGGAGACAAGGGTAGCTCAAGGGAGCACTCC
GACACCACTGATGACGAACAACACACTTTTA
CACTAAAACTAATGGACAAAGCACTCGTGAC
TTTCGATCCACATAACAAGAACAGGGATATC
ATAGCTACAGTAGAACAGGAGGCTAAGATCC
GTAAATCAGCAGAAAAGGCATGTTTATTATC
ACATGAACCCGCCAGACCATGGGTAGCCTCC
AGGGTGAAGAGTGACTTTAAACAATTGAGCC
TTAAGAGATTTGGGGAATACTCCACACTATC

GAACGCGGCAAAGTTGATGACCCAGCGTGTA
CCACGAGATTATCTCCTACGCCTTAAGAAAC
CAGGGGACGTGGACAATGCCCAGGGAGATAA
AAAAGTGATGTTCTTCCAATCATTGAGACCT
AGAATAGTAGGAATGATGTTGGACCCCGAGA
TACGCCAGATTGAAAGCAAGGCCCACACGGC
CGGACGGAAAACCACTGGCATAGCCAAAACC
ACCAGAGATAAAGAATGCCTCGATCTGGAGC
CTCCAAGGAATAATATTAGCGCCACACGGGC
ATTCCCCCAGGAGAGGGCGCCTAACCCAATA
CCCGTCCACAACACGCTGAATAACGTTCCAC
GGAACGAGGCCAGAGGAACATGGCACCCACA
ACCATGGAATGCAGAAGAGGGCGGAGGCTGC
GGAGGAAGAGAGAGTGACGGGCCATACCTAC
GCCGACGCCCACCAAACCAGTCACCATATCC
GCCGCCTCCACTAGCAATAGAGCATAGACCA
AACAGGAATCGATATGCTCACCACATTAGGA
AGCTCCTAGGGAGTATGAAGACTCCCATGCA
GGGAAGAATACCAACTATACCCACACAGGAC
CCAGAGCAAAGGTAGCCCAAGTGAACGTAGC
AAGCCTCGAAGCAATCACCGAAGTGGTACCA
GGGTACCATCTGGGGGAATTGATTCCCGTAC
GACGAGTCCACGTGGAAGTAATACTGCAAGA
CAGCCAGGGCCTTTGTGATACTGGAGCAGAA
ATATCCAGAGAATTATTTGACGAACTCCAGA
AGAGGAAGAATGAAACACTACAAATGATGCC
TACCTACTCAGGATGTGAAGGGACCCACATC
GGCAAAGTTATGACGAAGTTGACGTTCAACA
TGAACCATCCCCTATATGTCGTACAATACCC
ACTGATTATTGGGATCGAACTACTGAATCGC
ATTGTGAACCTTGAATCCCGACAAATTTGGC
TGCCGGAGCAACAAAGGGACTCAGAAGATGA
GAGAGACTCCACGGGAATCCAAGTGATAACA
GACGACCTATCAACGACAATCAGGAACGAAC
CCGCAGGAATTCGAATCACTCAGCTAATGAC
GAGAGAAGACCGAAAGGAAGCCGACTTAGAC
CCGAATGACCGTCCCCTCATGGAGTATATAC
GAGGCCAAACCGACGAACTGCAAACTGATGA
CAAGGGCAAGAGCGTATTACCCTCGGATGAA
ACTCAACCGATAGTAACGCAAAAGGAACTTT
CGCCCGAAACCACGAAGGCCCTAGATCCAAA
AAGAGTCCTTACAATAGAAGAACAAATAGAA
GAAGCAGCAGACGCCCTAGAGAGCGACACTC
AGCTACGACAACTGCTCCAAAAATACAACTC
CAAAGACTCCTATGATTGTGGCAAAACCGAC
GTATCCATTCCCACACTGGAGGGAGCACCAC
TGAAACAATACAAGATTCCACTGGCCGCCTA
CCAAGAAACCCTAGAGAACCTACTCAAAAAC
CGGGAATGCAACTCGACCTACAACTCACCCA
TACTAAAACCCACGGGAAAATGGAGACTCAC
CCGACAACTCAATAAAACAGTTCCCCTATCT
ATGGCAGAAATCGACCACGGTCTCAACCAAA
CAAAGGTACTCACCACAATGGACCTAGCAAA
GACCATGCCAGTAAAAGAAACCGATCAATAC
TTCACCTTCGACGGAATACAGTATACTTGGA
CGTTCGGATACTCGAACTCACCTGCAGACTT
CCTCCACAAGGCGATGGGCGACGCAAAAGAC
ATCGTCTACGTCGACGACATACTAGTCAAGC
GGGAAGAACACCTCGAGAGCCTCCAGCATAC
GTTAAAGGAAGCAGGCGCAAAGATTTCAATA
CAGTGGGCACGCAAAAGGGTGGACTACCTCC
TCGGCACTGAGGGAATGTTACCCCAAACCAA
AGCCCTCCTCGCACTCAAATCGCCCACCACA
CTTCGGAGCTTCTTAGGGATTTGCAACTACC
TCGTGGACGACTACGCAGGAATCGTTCGACC
ACTACTGCAAAAAGACGAACCATGGGAGTGC
CAGGAAGCCGCAGAAAAAGAATTAAAACGAC
AGGCATCCTGCCTAGCATACCCAGAGAAAGC
CTACCTGGAGACCGCATACTCAGATCATAGT
GTACTGTACCAGAGACAGGAAGCAGAGAAAC
CCTATGCCAGCAAAGCCCTCCGGGGAGTGGA

CTCCGAGTGTGAGAAGGCGATATTTGGAAA
ATACAACATTTTCGGAACTTGTTGAACGGA
TCCTGGAAACGAATCATGAATCCTTGGCATA
TAAGAAGATTCGAGAGGGCCGCGTAACCAG
GCCAGCTGGGCACTGACACTCCAAGGCCTA
TGAAATACGCAAAAAACAAGAAAAGTCCAAT
GTCTTGCCGATTTACATGATTGTACCTTTGA
GCAGAGGACGAAAAGATACCTCCGGAGGACA
TGCCCTTCAGGAAGGAGACATGTGCCGACCT
ATACATCGATGGATGCTCCAAGATGAAGGA
CACGCTGGGTCAGGAGTCATTTGGGAAACT
CAGGGATTCAAGAGGGATTTCAGCTGGGAC
CCAGTATGCAGAGTTGGTCGGGGTACACAT
ATGGCATCCGATAACCAAATACGGACCATG
CTGATTCCAACTACGTACAGCACAGCTTCCT
ACCCATATGGAAAAAAAACGAGATGAAGAA
AAACCAATTCGCCATCGAGAACTATTCGAG
AGATGGTTCAGAACAAGGATATGAAGATAT
AGTCAAAGGGCACTCCAAGATACCTGGTCAA
GGGAACGACCAAGCAGATGCCATAGCAAAA
TGGAAGGCACCCCGTGGAAGCTAGAAGACCA
AGACGGACAACAAGAGTCGACGAACCAAGA
ATAAGACTGGTCACAAGAAGTGGGAAGAAC
CTGGAACCAGAGCCCAATTGAACAGTGCCCA
AGACCTGATCAGAATGCAACAAGAGGACGAA
ACACTCGCCGATCATATCCAAGATCCAGCA
TCACTACTGACAACTTGAATGACAACAAAGA
CATGCATAGCCAAATCAAAAGGTTCTCAAT
CTACTGGTATACACAAACGAAAAAGACGGT
GGGTAGTCCCCTCTCAATATCGAGAAATAAT
CGCCCACAATGAACCGTGTAGCGGCCACCGA
AATACGGGACAGATGCTGCGACAGGTGGCCT
AAATGGGGGAAGACATACACAAACATGTGT
CGTGTGTTGTAAGTACCAGCCAACGACCTCA
GCACCCCTACAGAGGAGGGGAGCAGATTAT
ACCTACAAATCGACTGGATAGGACCTGTCAA
CAAAGGGAACAAGTACATGCTAACTGTGAC
TCTAAATGGACAGAGTGTTTACCAGCACCCA
CGGAAACAACCGCTATACTACTGATTAACCA
TAGATGGGGCCTACCGTCAACGATCGATTCA
AGCCACTTCACAGCCGAGGTCATCACAGAA
TGTTGGGAGTAAAAAGACAATTACATGTCG
ACAATCCTCAGGACAGGTGGAAAGAGCCAA
GTGAGCCTCCTAAAGAAATATGTGTCTACCA
ACTGGGATACGAAACTACCCCTAGTCTTGAT
AGCCACACCCAACCGAGCTACAGGCAGGACA
ATCATGACAGGGAGGCTAATGACCCTCCCA
TATATGTCCCAGCGGAGGACGAGACCCCGA
GCCAACTGAATACCTGACGGGACTCAATAGT
AGCACATTCGCTATGGTCAGGGACCAGTTG
CTCAGGGGAGCAAAGCATACTATGACCAAA
CGCAGAATACCAGCTAGGAGACCAGGTGTG
TACGAGAAAAGGAAACAGAAGTGCAGGAAAT
CCTGGACAGGACCACACGAGGTTGTTGACAA
AGTCGTATATCAGCTAAAAATCACAAAGACA
CAGCAATACAAGTGGGTGCACATCAACCAA
ACCCTGTGCTGGTGGAGCGAGATGAGGAATA
AAGGGGGGGGGAACTAAACTAATGGGCTT
AATAATATGTAACATGTTTTCCAGGGAATG
TGCTCACGCTCCTGCTAACGTACCTTGCCAA
GAGCCAAGAGCTGATCAAGCCAGGACCAGA
ATGCTGAGAGACTCCTCCAGCTTCCTAGTAA
AAATAGTAACCCAACAAGTCTATGTCTCCTT
CTTGGTGGTAAAGAAGCACTTCGGGAAGTTA
GCCACCCAAGCAACCCGAATATGGTATTAT
GATATAGTGAACAGAATATCGGAACGATCCT
ACAAAGAACCATGATACTTCCCCCCACCACT
ACACGTTCCAAGAGGTCTATCGGCCTGGGA
GAATACTATCCGGTTTTAGCTCCTTAATCA
ATCCATATCCAACGCCGTAAAGATTGGACA
GGTATCGAGAATTTACAGCTCGATATGCAT

AGCAAATACAACACCAACAAAAGAATCTGG
ATCCACCCTCCGAGACACCGTGGTTCTCGT
TCTATCATGATTAACTTAACATTCCAAGAA
CAATCCGAGGTCTATACGAAGAAGAGAGTA
AATTCAGTGGGTGATCCAAGACTTGTTGCG
GGCAGCGTCAGTGATCTTATACAAGGACAA
ATCTGGTAACTCCAACACTGATAAAAGATG
AGCATCCCCGGAAACAATCAGTCCAACTCA
GCCTTCAACATGGGTACAGCCACACCCATA
CCCAAACTAGACAGCTGGCGTTTTTGCTAA
GGTCAAACCCGGAAATGTATTCCGATTGCG
AACGTGGGTACATGGAATAACGACATTTAC
AAACTCCCTTAGTGGTGGCCTACCAGGAAG
TAAGTACCTGGTGCCTGACCTAAACCTGTG
AAAGAGGTTCATTGGCTCTGCCCAGGGGAA
CCAATACAGCAGAAATAATGTGTGGTCTGT
CTCCAAGGAAACTTGCAAGTTTAAGCTAAG
GAAAGAACCGGAACAGTGGCCATGATAGCT
GGTTGATTAGCACCCCTCTAGACACGGCAA
CGATCAACACTCCATAACCATGGAAGTTCC
CACGTCGCACTGGTCACAATACCAAGAGGA
GCATAGGGGACAAGGTATTATATAACCTAG
ACACCAAGCGGAGGTAGAAATAGTAGACAC
CACAAGTTTGAGATAAGCAGCACTCTGTCC
ACAGTAAAGAATTCCATATGGTGAAATTAT
ATCAGAGATTAACATAACAGATCTTACTTG
GTGGCTGGATATCAGCGAAATCAACAGATG
TCACAGGGGCAACAGGGATGATCATACTAG
TCTAACCTTAACATACCATGTGGCCAAAAT
CTTAAAAGACTAATCGCAGGCACAAGCCAC
AGCCCAGGAACAGGGATAGAGCCAAGGCCT
CCCAAGAATAACCGTAGAAACTATCTTTTT
CCTATAACACGTAGTCAATAGAATAATTTT
ATCGTTTTACCTTCCTTTAGAATTTTTTTTC
TTCCTCTCTCTTGTCACATATAACCATGTTA
ACGTACGCGCGCGTTGTAAGGAATAGCAGC
GACAATCGATGGTTCATGATGGAAGAAGGG
TTTTAAAAATAACCCGTGGCCATTTGACCA
TTAAAAGGGCGGAAGCACGCACGTGTGTTG
CTTCTTCCAAAATTGAGATTAATAATATTT
TTTATATATTATCTTTTTACTTGTTTTTTTTA
ACACACCAAAGCGCAGCAAACTGCAAAACA
TTAAGTAATGAACTGGAGGAGGGCTAGGAT
GCAACCTTGCTTCAAGGAAAGATAAGGCCT
CACATGTCACCCGTGCAGGCGGCAGAGACA
CAGCCAGACATAGTTAAGGCAAAAAGCCCA
ATGGTTTTAAAGTAACGTCAAAATTCGAAA
GAGTAGGCTAAATTATCGATCCTCGCGCTC
GAGACCAGGCTGCGAGCTACATCAATGAAC
TTGCTAATCCAATACTGAATGTTAATATTA
AACGAATTCTATGACCTCAGCACATGGGCA
CAGTGTCGACTCAAAGGCAATGGAAGAAAG
TTTGTCTAAATCACAACCACAGAAAGCCAG
TGATTGTGTGTTTAGGGATTCCGAGCTAGT
GCACACAGGGGGGAGGTAGAAGTACGCCTT
AGCTGAGGGAGCAAAGATAACCCCAAAAAG
TAAATTATTAAATCCAGGGTGAGTGGTCAA
TCAGCTCAAGGCTGTGTGCACAGGGGAGGG
AACAACCCTGCACGTCTGAACGTGAGGGGG
GAAATTAAGAATAACACTCCCAGAGCAGAA
CCACAGTGAGACGAGAATAAGAGTGTTGTA
GGATGCAGACCCAGCTGAACAACAAGGCTG
CAGAAGTTCGCGTTCGCCTAGTTAAGACAA
TATTCTGTATTGCTATTAAAGTAACGTTAA
TAACAACAGGGTAGGCTAAAGTTATTGATA
CTCCAATTAAACCAGGCCACAAGTTACGCC
TCACCATCATTAAGTCAATATGAAATGTTA
TTAATACTTTCTATACAACTCGACCCGAAG
TCAGAACCTTCCTAAGTCAAAATTGAATCG
GGAGCGAATAGTCGATATAAGGTTGAAGAG
TAAGAGCACGGCGAACTGCAAACTTAAGCA

AACGACACCAGCCCAGCAACAGCGAGTCGG
GCCATAAAAGTAGAAATAATAACTCCCGGG
ACAAGAAGAAGCAGGCAGACAAAAACCAGA
AGGTTCAACCGCCCTCACCTACAAGGAGTGA
ATGAGGCCTTGAGTTAACGGCGTCAGCTGGA
CAACGGCGAGCAGAGATAGGGTGGGGCAAA
CTATGGAATGCAACTTCGTGTCAGTAAAGC
AAGTTAAGTTAAAGACATCACAATACATAAT
AACATCAATGTTAACGCTATTCACCGTAAA
CTTATATAACCATGCAGACACCAAGGAATA
GGATCAAGAAACTTACCCCTTAACCTAAGAT
AAATTAATAATAATAAGCATATGCATGAAAA
AAAACTTATGGAAAAGCCAGGATAACGGGAT
CCAGGTCGAATATAAATACATAATAAAAAAA
TAAACCCAGGGTCGGAGAGAAAGGGAGATC
TTGTATCCCCGCAGCGCGGGATAGGAGTGT
GGGGAAACACCTCCACTTGAGTCAAGCACCA
ACGACAGACCTTAGGTTACATACAGAATCAT
ATAGTAAGGGACAAATGTTAACTTAAAACT
TTCTTTGTGCGCCGGGTCGGAGAGAGTGTT
CCAGGTTGTGCAACACGAACAAAGACGCCTA
TTGTAACGTCTGTGTTTTGGGTTCATGAAGA
AAGAGATAATATTGCACCCAGACAGAGAGA
GCAATTTGTAACATCTGATTGTTTGCTCGT
GAGGGAGTGTGACAACGGAATATACCGTGG
CAAGGACATACGGACGTACGGACACACACAT
TGCCTACGGACAGATCCTGACACAGCAAAAA
GCCCCGCCTTGTGGGCGGGATCCAGGGGGAT
TAAAGGAAAGGATCCTGAGCATTGGAAACTA
GCGGGTCGACTCAGGAGCGAGCTCGACCGG
TCAGGAGCGAGCTGCGACCAGCGGGTCGGCT
TTCAGCTCGTCTGGGACTCAGCCGAGTTCGA
CAAAGCCGGCTCTCGGGGCTCGACTCAAGC
CTCCTCTGCAGGCGGGCAAGACCCGCTTGA
CGGCCTGCGTGGGTCAACCTCTGCCGAGTC
GCTGCCGCCGGAGTGGTGAGGGTCGCGGTT
GCTCCGTGCACACCCTGGTCGACGACCCAA
TGCGAGCGAGTTGTGGGCGCCACCTACCGA
AACTGCGACAGCGTATCAACGGGCATCCGAA
CACGGTGACAGGGGAGCGACTGATACGTAT
GCCCCCTCACCACACGACCACCAGGCGTCT
AAGAATCGACTCCGAACACTGACTATTCAA
ACCACTCAAACACACTCGAACCCATAAGCCA
GGAAGGCATCACAACGAGACCGAGTGATG
GTTTATAAAAGTATTCGTAATTTTTATCTTT
TGGGCTGTACTCTTAATCGCATAAATTCCCA
TCAATACGAACTGATTCTTGGCACCTCCTT
ACGCTCACGTGGGGAGTGGTGGCCCACCGGA
GGTGATCTGGTCCCCAATTAGTAAACTTGGT
CGATCTTCGGAAAAGGAGCACCGTCGCAGG
GAGCCGTTCTGTTGTCAGATAACCTGCCGAT
GAGATTGGTGCCTTTAAGAATAATAACTGTT
GTTTTTTTATCGCCGTATAATCTTTCAAGTA
CATAATCGCCGCACGTACTCGCATCCTCTA
AACTGTCCCGTGACCCAAGGTGCTAACAAC
TTGCGTATGATCAATAAGGAGTGCGGTACT
TATTGAGTATAATCATTCATTAACGTTAAG
CATAGTATTTGCATATTTTAACGACTCTCT
CAACCATCGCGTTATGCGGGATCCATGTGC
GTGAAATCAGACAATCGTTGATGATACTGTA
TGTTGACGTTCCGGGATTGAGAGTTCGCATA
AGTTAAGGTTGAATTAGTAATAAATTGATCA
GTGGTTGTGTGATTCTTTCCTGAAATCTATG
GCTTCTGCGTCGAGATCTGAATTTGGTGAC
ACACTAATACATACTCAGTAAATGTTAAAG
GTGAACCGGGGACCTAAAATATAACCACGAA
TGGGAATGTTAAAGCGAGAATGAGACGAAC
ATGCCGAGTTAATCTAAGTTAATCTTGCATA
CACCTGCATAACTGCATACGATTAATTGCCT
CGATAACCCACCCTCGTCACCGAGGCGGGGT
ATAACGACTTGCATTATCATAATTACACCCA

TTAATACGATTACCTCTCGAATTGGCATAAT
CATAATTACATAATTAACATATTTCCGGATA
TTGGCCAAATTTCATTTAATTATTATTATTT
ATTCATAACCCACATATACAACATAATAATC
CACGCATACAACATAACAAATTTTGATAACC
AACATATACAACA
>ERV-Spuma.0-Sca
AAATACAGAAAAGGTGTCATTTGAAACATTC
AATATCTGGTCTGAGAGGGTACAGGGAAAGA
TTAAATAAGACACTTTCCCTGCCCTTGACGC
TCCCGCCCCAGCGCTCAGGCCCTCCCCCCAC
TTAAGCCCCGTAGCCCATCCCCTATCGGAGC
TTCCCGCCAAAAAAGTAGCAAGTGCGGGCTC
CCCACCCACGAGGCAGCAGCAATCAGGAGA
GAGGGAGCAGCAGCTGGAGAGGTCAGAACGC
GAAGGAGGACAGAAAACAGAGAACCAGCAT
GAGGAGGAGAGAGAGAGAGAGAGAGAACTT
AAGGCAAGGCAAGCAGCCGAGTGTAAACTGT
AAAGAAGGTTAGGATTCAAAGTTAAAATGCT
CTGAAGGTCCCTACAACCAACCAGACAGCAT
CTAATCCTTTTACCTTTCTGTAAAGAAAGCC
TAAAAAAAAAGTATAATAATAAAATAACTTA
AGTGGTTATTAGCTTACTTCACTTCCTTAAT
CCCAGGACATCGATGCTATTAAGGGGTAAGT
TCTTCGGTGAAGGTATGGGTTATACCGGGGC
AAAGATAGTTTGACCTGAGTAGCACCCACTC
TCGGAGTCAGGGAGTGAGAATCCCTGTTCAC
TATTGGCCCTTTTTGGTATGGGGGAATTCTA
GTGAATTTGCAAAAACATGGCGCCCAGCGTC
AATATTAGAAGTTTCTAGGTAAAGCCTAGAA
TTTCTAGTTAAGCGAGTAGGGGCTAGAATAT
CTAGTTCCCAAGAAAAGCCTAGAATATTAGA
GTAAAGCGAGGCTAGAATATTAGAAGTTTCT
GAGTAGGGGCTAGAATATTAGAAGTTTCTAC
AACGGTTGGAATATTAGAAGTTTCTAACCGC
TAGAATATTAGAAGTTTCTAGTCTATGTGTC
TTACCTGCCGAGTTTAGTTTGGAAAGTCATC
CTTTTGTAAGGATATTCTGTGGATCGAGGGC
CAGGTTGGGTGTGAAAATCAGTAGACTAGAA
CCTGAGAAAAAGTCTGCAAGACATTTTGGTC
AAATCTTGCATCCATTACTGGTAAAAGGGGC
AATCATCCTCAGAGTAAGGCAGATTGTGCGC
GGAAAATAGGGACGCTTATATTCAGAAACCA
CGACCCTTAACTAAAACAGGAAGATAGTGCC
CTTGGATAGGGCCCAGAAAGGGTACTTATCC
GAGTGCACCTGAAAGGGACAACCAGGAACAC
TCCGAAAATGGCAATTAGGGTAATTCTCTTC
ACGGTCAAGAGAGATGTAGCAATGAAACAGC
AATTAAAAGAGCAAAAGAAATCCTGGACAAC
TCCAGACGGAGATATCTCCCTCTTTGTTAGA
GCCCTGCAATATACAGATTCAGTCAGGGTCC
TTTGTCAGTTTGCATTTGAAAAGCGAGCTGA
CGTATTTGGACACAGACAGAAGCATGCAGCT
GGCAAAGTGTGTTCCAAACTGATTAAAATCA
TAAACAGGACAGAAATAATACAGATCCATAC
AATTAACTCTGAAGTGGTAGGCAAATATATA
CAGAAAAATGAACCATTAGAAATGGAAAGTC
CAGGCCTAGAATCAGATAGCCGAGAGAATTC
TGAAAATTTCCCAACGGGATACAATTTTCCA
CCAAGTCTGTTTCAGGAAGAAAATTGGCCTC
CCACACATAAGAAAGAAGCGTTTATCGATCA
CCACAAGAAAGTGAAGAGGAACAAGGCATA
ATGGCGCCTCCTGTGATGGGACAGGAAGAAT
CATCCGAGAATGAGGAAATAGTCAAACCAAA
AGGGAATAGAAAAAATTCTAAACCATTAGAA
TATAGCGATCACTGGAAACACCAACAACCTT
AACCAGTAAATAAGGATGTACCGTATTATAT
AGAAGCAGAAATGGTACACCATAGAGATCAC
GAAAGAGAGGTTACAGCTAGGGGACTTAAA
TATTAGGCGGAAGTCAACAAATTGATTCTAC
AGAATTACCAAACGTTACCCCCAAAGCTAAA

ATGAGCAAAGGGTATGACAGTTTCGGAAGA
GGTAAGGAACAGCACAGAAACACAAGGCC
AGCACTAAGACCACAGGAAAGGATACAGAA
CCCCAAAAAACTGCTCCTACTCCAATGGAT
ATGCTCCACGAGATAGCCAGGTACCAAGTG
AGAATATATAGAATCGGTGGAAGAGTTTGC
ACCCCCTATTTTGAGGAAGAACCGGTACGA
ATTTCTCCGAATATAGGGACCCAGATAGAG
AGAACACCATGCCCCAGAAGTGAGAAAAT
TTACACCAGGTCCAGAGGAAGGACAGGGA
CGTATAAAATAGTAGCAGCCGTAATGAAGA
AAAGAAACATGGGGAGAGTAGAATTGAGTA
TTAAAACAGTTAGCGAAGAAAATGGGGGCA
CCACCAAATTGATTGATTTACATACAGTGG
TACAGGCGAAGTCTCAGATTTAATAAATGA
GAAATGAGAGAGCTAATTTAATGAAAGAG
CAGCATATGCAGGGGAACACCTCACACCAT
AATATATGAGGTTAATAAGGAAGAGGGGGT
AGATTCCTGAGGGAATTAGGACCCAAGATT
AAAGCCTCTCAATAATACTGTTAACGACCT
AATAGGAAGAGAACTGGTTAAATTTGATTT
GCATTTAAGGATAAACACACAAAAGGGGAA
CAGCTGCCCAAAATAATGTTTCAACACAAA
TAGACCACCAAACCGACAGATACCTGACAA
AATCCAGCACAACCAGCAATGCCACAACAT
TGCGGTCACAAGGGCCACCTCCACCACAAA
TTTACCAAGGGAACAATTCCTAGCCCTCAG
AGAGACAGATTCATTGAGGATAGGAGAAGA
GTCAGGGGAGGATTGAGCAAGGTAGATATC
AGGGTATCCGGGACGCTCGCGAGGGGGATT
GGCAGAGGAGGCCCCGGTATATATTGACAG
CTCCCCTTAAACAGGCTATACAAAATCTAA
AGACAAAGGAGAATTAGTAGTAGTATACAA
TTTATAGTGGACACAGGGGCTGAGATATCA
AAGAAGCTGGAAATCCGACAGGAAAGAAAT
AGGAGCTTTAGGGAATAAAGATATTGGACC
TTAGGAGAATTACTTGCAGTATCAGGAAAT
TGGCAGGGAAAGATTTAATAAAAATAGATG
AAGGATATTAAAACCAATTGATAACATAGA
AAACAGAAAGAGAAAAGTATTTTAAATGAC
AGAAGATGGAAGAAGTACTGATGGGAGCAA
AGGTAAGAATGACACAGGTTTAATTAGCAA
CACACAATTCACGGGGGACAACACAGACCT
ATCCTTTGACTAGAGGAGCTAAGAGCGAGC
AATCAAGGAATTGGAGCAGCAGGGGATTAT
ACATATGCCTCAACAAATAGTCCACTTCAG
AACCAGATGGTACATTTAGAATGGTAACAA
TTTAAATAAAGTCACAAAGAAGGACAAGAG
AATCCACAAGCTACCTTAGAGCAAGTCGCG
ACTTAACTAGTATAGATTTGGCGAATGGAT
TCCATTAGACCCAGACAGCAGGGAGAAAAC
TTTGGAACTAAGCATTATGTATATTGTAGA
GATATGTTAATTCTCCGAATCACTTTCAGG
GGAGCTGATTAAAGATGATTTGGCTTTAGT
GATATATTAATTGGAGATGACGATCAGGAC
AAAGAGTGGCCAGGATTATTAAGACACTTA
CTTTAAAATAGGGCTAAAGAAATGTCAAAT
GAAGTCAACTATTTAGGTTATTCAGTATCG
GAGAGGCCAGTATTGAAATGAGGAAGAAGG
TACTGCGCCTGTTTCGAAAAAAGGGGTTCA
GGAATGTTGGGATATCTAAGGCCAGTAGTG
GTCTTTATGCTAAAGCCATTTATCAAACCT
TTTTATTTGGACCAGCGAGGCCCAGAGAGG
TTAAAGACAGCAATTGCGATATCGGGGCCA
GACAAGAAGAGGATGATTTAAGCATTAAAT
TAAAAATGGATATGGTATGGTGCTTGCTAA
CAGACACCTATCAAACATCTGACAGGAAAC
CTGAACAAAAGTTTTCAAACATCGAGAAAT
CATAACAAAGAGAATAGCAGAGATAGAAAA
GGTAAGAAAATTTATGTCACAACTGAGTTT
AGGAGCTAACCAAGAGACAGATTTGCCAGC

TACAGCTCGATGGGAAAGATGGGAAACCAT
CCTGACTTAATGTTTATTCATAACATTAAA
AACTTGATGAACCAGTTAGTAAAACGGAGT
TTGGGTATTATATACTGATGGTAGTAAAGT
GAAGAACAAGCCAGATGGGCATTCATTTTA
AAACATAGTAGTGGAGGAACAGGGAGTAA
GCTCAGACAGCTGAGGTAGAGGGAGTATT
AGGAGTGTCACCAGCGAAAGCTGCAGGAA
TAACCGATAGCTTTTTCGTGCAGCAAGGAT
TTTGGAGTTTTGGAAACTAAACGGTTGGAC
AATAAACTACTGGAACAGAAAGAACAGTGG
ATAAAATTTTAGCCGATATGGAGATCCAGA
AAAGCACACACAAAAGGAATTGGGGAACT
AATCAGGAAGTGGATGAATTAGCCAGACTT
TCTTGGGGACTCTTGGGAACTCAGCAGCCG
ATAATTAAGCAAGTACATGAAGCCACAGG
CATGTTGTAGTCGCAAGGGAGTTGAAGAAG
AAATAGCCTATTGGAGACAACATTTAACTG
AAAATGTGAGAGATGTATGGCCTGTGGAGG
AATAAAACATATGGGAGCATAAAAACTGAG
AGGAATGGTCATTAGATTATGCAGGACCAC
AAGCAGCAAAGGTAATTTGTATTTTTTAGT
GCTGCACGGGAGAAATTGACCTTAGAGCA
AAATGGAGCCACAACCAAAAGGGTATTGG
GACCTCCAGAGGTAGACCAGAATCGATCCG
GCGCCACACTTTAGAAACAATACTGTTATT
TGACAGAGGAATAACAATCAATTGGGCCG
ACCTGAGAGTAATGGAATGGCTGAGAGGTC
GTAAAGGATTGGATCATAAAGAATCAGAAT
GGGATAAAGACATTGAAAGAATAGTATTTG
AACCTTGGAACCTCTGTCCCCCAGGGACA
AGTGAATCTGGGAATTATCAAGTAGGAGAG
GAAGCTACCAACTAAGACTGCAGGGAAAA
GCTGTAAAATGTAAAGACAAGATTGTTCA
CACATACAGTTGAATTAGAGAAGCATGGT
AAAGAAATTGTATCACAATTAAAACACATG
CATCAGAGGAGTTTGAGATTCTTCGAATCT
GCAATGACAGAAGGAGGCTTAACAGGCTT
ACATCAAGGATAAGGAAAATAAACCAGTT
TGGTGGCAAAAGAAATCCGGAAGTCCTGGA
TGGGATAATCCAAATTAAAGAATAGGGCC
TTGATCTGTTCATTTGATTTTTCAGATGGA
ACAAACCAGTTCACAAGAAGGAAGAGAAC
GAAGAATCTTTTAGACAGGATTGGGGGGA
ACAATTGGCTACATATTTGAGGGAATTAAA
CGGAGGAAAACTTTACGGGTTAGAAACAAA
AAATTATTACTGAACAGTGAAACCTCCGGT
TAACTGGACATTAGCTGATGGATGATCTGT
GGTATGTACTACGCGGTGGAGTTATTAAG
ACAGCAGCGACTGACGAGGAACCCCAATA
CAATAGTTTAAGAATTTTTGTAGCTTGATA
AAAATTGTTACCAATGCAGCAAAACAATAT
AGCTGTAATCGTTATTAAAAAAAAAAATTT
ATAGGGACTGTTATATATATATGATAAGG
TGTCGTTAAAGAACTTGATCCGCATTTCTA
ATGTTTTAATAGAGGGGTAGTTAGTATGTA
TCCCAAAGCCAAGAAGGGATGTTTCGATA
ATTGTTAACAGTTGAAAATATGTAAAGTTG
TCTATTTGTGTGGTTAGGTAATGAATAATG
AGATAAAATTTTTACTATGTATTTAACAAT
GTTTGGAAAAAAAAAAAAGAGACGGTTCA
CAAATGCTGAAATGAAAAATATACACGTT
TATTCTCTCATGTAAACCAGGGGTTGGCAG
TCACGACAATGTTAGACATTAATGGGGAA
CAAGCAGGATCAATTGAACATTTTAAATTA
GGGATCAGTGGGTGGTACATGATATCACAA
CAATCAGAAATGGAAAACAATTTGGACTA
AGACTCGTATTGACACTGGTCTGTGATATG
ACAAAGATCGGGGATGTATGTTAGTACAAT
GACAATAAACCAAATTATAGTAATGTTTCA
CGGGGTGTTCTTGAAAATACAGAAAAATGT

ACATGGAAGTCTGGCAATATCTGGTCTGAGA
GAAAGATCCCACAAAAGGTTAATAGCAGCTG
GAAACTGTGGCCAGCCTTTGCATGTTCTGGA
AAAGGCGTCATTTGAAACATTGAAGTCTGGC
TCTGAGAGGGTACAGGGAAAGAATAAACAT
ACACTTTCCCTGCCCTTGGCGCCTGTCTGAC
AGCGCTCAGGCCCTCCCCCCACCATGCCCCT
GTAGCCCATCCCCTATCGGAGGTCCCGATCT
AAAAAGTAGCAAGTGCGGGCTCCTCCTTCCC
GAGGCAGCAGCAATCAGGAGAGCAGAACGCA
CAGCTGGAGAGGTCAAACGGAGCACAGAGG
GAAAACAGAGAACCAGCATCAGGAGAGAAG
AGAGAGAGAGAGAGAGAGAACTCGGTCATG
GGCAAGCAGCCGAGTGTAAACTGTAATAGA
GCTAGGATTTAAAGTTAAAATGCTAAGGTG
TCCCTACAACCAACCAGACAGCATCCAGAA
TTTTACCTTTCTGTAAAGAAAGTGTTTGCAA
AAAGTATAATAATAAAATAACTTAACCTGAA
ATTAGCTTACTTCACTTCCTTAATAACGCAC
CATCGATGCTATTAAGGGGTAAGTAGGTAAA
TGAAGGTATGGGTTATACCGGGGGATAACT
GTTTGACCTGAATAGCACCCACTGAAGCCTC
CAGGGAGTGAGAATCCCTGTTCACCATTTAC
CCTTTTTGGTATAGGGGGAATTCTAAGAAGA
GTGGTGAATTTGCAAAAACA

Supplementary Table S5a The conserved domains of ERV genomes.

ERV-Epcilon.0-Ebu	ERV-Spuma.a-Ebu	ERV-Spuma.0-Pma	ERV-Spuma.0-Sca	ERV-Spuma.0-Lca	ERV-Spuma.0-Lre	ERV-Spuma.a-Etr	ERV-Spuma.a-Lca	ERV-Spuma.a-Lre	ERV-Spuma.b-Lre
RT_ZFREV_like*	retropepsin_like_LTR_2	RT_LTR	RT_LTR	rve	RT_LTR	RT_LTR	transpos_IS481	transpos_IS481	RT_LTR
RT_Rtv	RT_LTR	RT_ZFREV_like	RT_ZFREV_like	transpos_IS481	RT_ZFREV_like	RT_ZFREV_like	rve	PHA03247	RNase_HI_RT_Ty3
RT_LTR	retropepsin_like	RVT_1	RT_Rtv	RT_LTR	RVT_1	RNase_HI_eukaryote_like	Integrase_H2C2	RT_LTR	RT_RNaseH
RVT_1	gag-asp_proteas	RNase_HI_eukaryote_like	RVT_1	RT_ZFREV_like	RNase_HI_eukaryote_like	RVT_1	transpos_ISNCY_2	RNase_HI_RT_Ty3	RT_RNaseH_2
HERV-Rb-like_HR1-HR2	retropepsin_like_LTR_1	RT_RNaseH_2	RNase_H	RVT_1	RT_Rtv	RT_Rtv	transpos_IS3	RT_RNaseH	RVT_1
Ebola_RSV-like_HR1-HR2	retropepsin_like_bacteria	RT_RNaseH	RNase_HI_prokaryote_like	RNase_HI_eukaryote_like	RT_RNaseH_2	RT_RNaseH_2	PHA03247	RT_RNaseH_2	RT_Rtv
ENVV1-like_HR1-HR2	Asp_protease_2	RT_Rtv	RNase_HI_eukaryote_like	RT_Rtv	RT_RNaseH	RT_RNaseH	transpos_IS30	RVT_1	RT_ZFREV_like
Ebola-like_HR1-HR2	RP_DDI	RNase_HI_RT_Ty3	RnhA	RT_RNaseH_2	RNase_HI_RT_Ty3	RNase_HI_RT_Ty3	RT_LTR	RT_ZFREV_like	Integrase_H2C2
RT_DIRS1	RNase_HI_RT_Ty3	RnhA	transpos_IS3	RT_RNaseH	RnhA	RnhA	RNase_HI_RT_Ty3	RT_Rtv	transpos_IS481
RT_like	RT_RNaseH_2	RNase_H	rnhA	RNase_HI_RT_Ty3	RNase_H_Dikarya_like	RNase_H	RT_RNaseH	Integrase_H2C2	RNase_HI_RT_DIRS1
TLV_coat	RT_RNaseH	RNase_H_Dikarya_like	transpos_IS481	RnhA	RNase_H	RNase_H_Dikarya_like	RT_RNaseH_2	retropepsin_like	RT_DIRS1
Ebola_HIV-1-like_HR1-HR2	RT_LTR	RNase_HI_prokaryote_like	RNase_HI_RT_Bel	RNase_H_Dikarya_like	RNase_HI_prokaryote_like	RNase_HI_prokaryote_like	RVT_1	RT_DIRS1	gag-asp_proteas
HTLV-1-like_HR1-HR2	RVT_1	RNase_HI_RT_Bel	RT_DIRS1	RNase_H	RNase_HI_RT_Bel	RNase_HI_RT_Bel	RT_Rtv	retropepsin_like_LTR_2	retropepsin_like
RT_nLTR_like	Integrase_H2C2	rnhA	RNase_H_like	RNase_HI_prokaryote_like	Integrase_H2C2	rnhA	RT_ZFREV_like	gag-asp_proteas	rve
RNase_HI_RT_Bel	transpos_IS481	Integrase_H2C2	RNase_H_Dikarya_like	RNase_HI_RT_Bel	rnhA	rve	retropepsin_like	retropepsin_like_bacteria	retropepsin_like_bacteria
RNase_H	transpos_IS3	rve	PRK08719	rnhA	rve	Integrase_H2C2	gag-asp_proteas	RNase_HI_RT_DIRS1	retropepsin_like_LTR_2
RNase_HI_eukaryote_like	rve	Rnase_HI_RT_non_LTR	rve	Rnase_HI_RT_non_LTR	Rnase_HI_RT_non_LTR	RNase_HI_like	RT_DIRS1	Asp_protease_2	RT_like
rve	zf-H2C2	PRK08719	RNase_HI_like	PRK08719	PRK08719	Rnase_HI_RT_non_LTR	RNase_HI_RT_DIRS1	RVP	Asp_protease_2
RNase_HI_prokaryote_like	RT_Rtv	RNase_H_like	Rnase_HI_RT_non_LTR	RNase_H_like	RNase_H_like	PRK08719	retropepsin_like_LTR_2	zf-H2C2	PHA03247
RT_ZFREV_like	rve_3	RNase_HI_like		RT_DIRS1	RT_DIRS1	RT_DIRS1	Asp_protease_2	RT_like	ZnF_C2HC
RnhA		RT_DIRS1		RNase_HI_like	RNase_HI_like	transpos_IS481	RT_like	PHA03247	
RT_RNaseH_2		retropepsin_like		RNase_HI_bacteria_like	RNase_HI_bacteria_like	RNase_H_like			
RVP		transpos_IS481		retropepsin_like	transpos_IS481	RT_like			
Rnase_HI_RT_non_LTR		RNase_HI_bacteria_like		RT_like	retropepsin_like	RNase_HI_bacteria_like			
rnhA		RT_like		retropepsin_like_LTR_2	RT_like	retropepsin_like			
transpos_IS481		PRK12678			retropepsin_like_LTR_2	RNase_HI_RT_DIRS1			
RP_RTVL_H_like		retropepsin_like_LTR_2				retropepsin_like_LTR_2			
HIV_retropepsin_like		RNase_HI_RT_DIRS1							
RNase_H_like									
RT_RNaseH									
Integrase_H2C2									
transpos_IS3									
zf-H2C2									
retropepsin_like_bacteria									
MLVIN_C									
zf-CCHC									

*Red：Duplicate Values

Supplementary Table S5b The intersection dates of ERV genomes con

Group	Intersection_Number	Intersection_Elements
ERV-Epcilon.0-Ebu	12	HERV-Rb-like_HR1-HR2,Ebola_RSV-like_HR1-HR2,ENVV1-like_HR1-HR2,Ebola-like_HR1-HR2,TLV_coat,Ebola_HIV-1-like_HR1-HR2,HTLV-1-like_HR1-HR2,RT_nLTR_like,RP_RTVL_H_like,HIV_retropepsin_like,MLVIN_C,zf-CCHC
ERV-Epcilon.0-Ebu,ERV-S	8	RNase_HI_RT_Bel,RNase_H,RNase_HI_eukaryote_like,RNase_HI_prokaryote_like,RnhA,Rnase_HI_RT_non_LTR,rnhA,RNase_H_like
ERV-Epcilon.0-Ebu,ERV-S	4	RT_Rtv,RT_LTR,RVT_1,transpos_IS481
ERV-Spuma.a-Ebu,ERV-Sp	3	retropepsin_like_LTR_2,retropepsin_like,RNase_HI_RT_Ty3
ERV-Spuma.a-Ebu	3	retropepsin_like_LTR_1,RP_DDI,rve_3
ERV-Spuma.0-Pma,ERV-S	3	RNase_H_Dikarya_like,PRK08719,RNase_HI_like
ERV-Spuma.a-Lca	2	transpos_ISNCY_2,transpos_IS30
ERV-Spuma.a-Ebu,ERV-Sp	2	gag-asp_proteas,Asp_protease_2
ERV-Epcilon.0-Ebu,ERV-S	2	RT_RNaseH_2,RT_RNaseH
ERV-Epcilon.0-Ebu,ERV-S	2	RT_ZFREV_like,RT_DIRS1
ERV-Spuma.b-Lre	1	ZnF_C2HC
ERV-Spuma.a-Lca,ERV-Sp	1	PHA03247
ERV-Spuma.0-Pma,ERV-S	1	RNase_HI_RT_DIRS1
ERV-Spuma.0-Pma,ERV-S	1	RNase_HI_bacteria_like
ERV-Spuma.0-Pma	1	PRK12678
ERV-Epcilon.0-Ebu,ERV-S	1	RVP
ERV-Epcilon.0-Ebu,ERV-S	1	retropepsin_like_bacteria
ERV-Epcilon.0-Ebu,ERV-S	1	zf-H2C2
ERV-Epcilon.0-Ebu,ERV-S	1	transpos_IS3
ERV-Epcilon.0-Ebu,ERV-S	1	rve
ERV-Epcilon.0-Ebu,ERV-S	1	Integrase_H2C2
ERV-Epcilon.0-Ebu,ERV-S	1	RT_like
ERV-Spuma.a-Lre,ERV-Spu	0	
ERV-Spuma.a-Lre	0	
ERV-Spuma.a-Lca,ERV-Sp	0	
ERV-Spuma.a-Lca,ERV-Sp	0	
ERV-Spuma.a-Etr,ERV-Spu	0	
ERV-Spuma.a-Etr,ERV-Spu	0	
ERV-Spuma.a-Etr,ERV-Spu	0	
ERV-Spuma.a-Etr,ERV-Spu	0	
ERV-Spuma.a-Etr,ERV-Spu	0	
ERV-Spuma.a-Etr,ERV-Spu	0	
ERV-Spuma.a-Etr,ERV-Spu	0	
ERV-Spuma.a-Etr	0	
ERV-Spuma.a-Ebu,ERV-Sp	0	
ERV-Spuma.a-Ebu,ERV-Sp	0	
ERV-Spuma.a-Ebu,ERV-Sp	0	
ERV-Spuma.a-Ebu,ERV-Sp	0	
ERV-Spuma.a-Ebu,ERV-Sp	0	
ERV-Spuma.a-Ebu,ERV-Sp	0	
ERV-Spuma.a-Ebu,ERV-Sp	0	
ERV-Spuma.a-Ebu,ERV-Sp	0	
ERV-Spuma.a-Ebu,ERV-Sp	0	
ERV-Spuma.a-Ebu,ERV-Sp	0	
ERV-Spuma.a-Ebu,ERV-Sp	0	
ERV-Spuma.a-Ebu,ERV-Sp	0	
ERV-Spuma.a-Ebu,ERV-Sp	0	
ERV-Spuma.a-Ebu,ERV-Sp	0	
ERV-Spuma.a-Ebu,ERV-Sp	0	
ERV-Spuma.a-Ebu,ERV-Sp	0	
ERV-Spuma.a-Ebu,ERV-Sp	0	
ERV-Spuma.a-Ebu,ERV-Sp	0	
ERV-Spuma.a-Ebu,ERV-Sp	0	
ERV-Spuma.a-Ebu,ERV-Sp	0	
ERV-Spuma.a-Ebu,ERV-Sp	0	
ERV-Spuma.a-Ebu,ERV-Sp	0	
ERV-Spuma.a-Ebu,ERV-Sp	0	
ERV-Spuma.a-Ebu,ERV-Sp	0	
ERV-Spuma.a-Ebu,ERV-Sp	0	
ERV-Spuma.a-Ebu,ERV-Sp	0	
ERV-Spuma.a-Ebu,ERV-Sp	0	
ERV-Spuma.a-Ebu,ERV-Sp	0	
ERV-Spuma.a-Ebu,ERV-Sp	0	
ERV-Spuma.a-Ebu,ERV-Sp	0	
ERV-Spuma.a-Ebu,ERV-Sp	0	
ERV-Spuma.a-Ebu,ERV-Sp	0	
ERV-Spuma.a-Ebu,ERV-Sp	0	
ERV-Spuma.a-Ebu,ERV-Sp	0	
ERV-Spuma.a-Ebu,ERV-Sp	0	
ERV-Spuma.a-Ebu,ERV-Sp	0	
ERV-Spuma.a-Ebu,ERV-Sp	0	
ERV-Spuma.a-Ebu,ERV-Sp	0	
ERV-Spuma.a-Ebu,ERV-Sp	0	

ERV-Spuma.a-Ebu,ERV-Sp0
ERV-Spuma.a-Ebu,ERV-Sp0
ERV-Spuma.a-Ebu,ERV-Sp0
ERV-Spuma.a-Ebu,ERV-Sp0
ERV-Spuma.a-Ebu,ERV-Sp0
ERV-Spuma.a-Ebu,ERV-Sp0
ERV-Spuma.a-Ebu,ERV-Sp0
ERV-Spuma.a-Ebu,ERV-Sp0
ERV-Spuma.a-Ebu,ERV-Sp0
ERV-Spuma.a-Ebu,ERV-Sp0
ERV-Spuma.a-Ebu,ERV-Sp0
ERV-Spuma.a-Ebu,ERV-Sp0
ERV-Spuma.a-Ebu,ERV-Sp0
ERV-Spuma.a-Ebu,ERV-Sp0
ERV-Spuma.a-Ebu,ERV-Sp0
ERV-Spuma.a-Ebu,ERV-Sp0
ERV-Spuma.a-Ebu,ERV-Sp0
ERV-Spuma.a-Ebu,ERV-Sp0
ERV-Spuma.a-Ebu,ERV-Sp0
ERV-Spuma.a-Ebu,ERV-Sp0
ERV-Spuma.a-Ebu,ERV-Sp0
ERV-Spuma.a-Ebu,ERV-Sp0
ERV-Spuma.a-Ebu,ERV-Sp0
ERV-Spuma.a-Ebu,ERV-Sp0
ERV-Spuma.a-Ebu,ERV-Sp0
ERV-Spuma.a-Ebu,ERV-Sp0
ERV-Spuma.a-Ebu,ERV-Sp0
ERV-Spuma.a-Ebu,ERV-Sp0
ERV-Spuma.a-Ebu,ERV-Sp0
ERV-Spuma.a-Ebu,ERV-Sp0
ERV-Spuma.a-Ebu,ERV-Sp0
ERV-Spuma.a-Ebu,ERV-Sp0
ERV-Spuma.a-Ebu,ERV-Sp0
ERV-Spuma.a-Ebu,ERV-Sp0
ERV-Spuma.a-Ebu,ERV-Sp0
ERV-Spuma.a-Ebu,ERV-Sp0
ERV-Spuma.a-Ebu,ERV-Sp0
ERV-Spuma.a-Ebu,ERV-Sp0
ERV-Spuma.a-Ebu,ERV-Sp0
ERV-Spuma.a-Ebu,ERV-Sp0
ERV-Spuma.a-Ebu,ERV-Sp0
ERV-Spuma.a-Ebu,ERV-Sp0
ERV-Spuma.a-Ebu,ERV-Sp0
ERV-Spuma.a-Ebu,ERV-Sp0
ERV-Spuma.a-Ebu,ERV-Sp0
ERV-Spuma.a-Ebu,ERV-Sp0
ERV-Spuma.a-Ebu,ERV-Sp0
ERV-Spuma.a-Ebu,ERV-Sp0
ERV-Spuma.a-Ebu,ERV-Sp0
ERV-Spuma.a-Ebu,ERV-Sp0
ERV-Spuma.a-Ebu,ERV-Sp0
ERV-Spuma.a-Ebu,ERV-Sp0
ERV-Spuma.a-Ebu,ERV-Sp0
ERV-Spuma.a-Ebu,ERV-Sp0
ERV-Spuma.a-Ebu,ERV-Sp0
ERV-Spuma.a-Ebu,ERV-Sp0
ERV-Spuma.a-Ebu,ERV-Sp0
ERV-Spuma.a-Ebu,ERV-Sp0
ERV-Spuma.a-Ebu,ERV-Sp0
ERV-Spuma.a-Ebu,ERV-Sp0
ERV-Spuma.a-Ebu,ERV-Sp0
ERV-Spuma.a-Ebu,ERV-Sp0
ERV-Spuma.a-Ebu,ERV-Sp0
ERV-Spuma.a-Ebu,ERV-Sp0
ERV-Spuma.a-Ebu,ERV-Sp0
ERV-Spuma.a-Ebu,ERV-Sp0
ERV-Spuma.a-Ebu,ERV-Sp0
ERV-Spuma.a-Ebu,ERV-Sp0
ERV-Spuma.a-Ebu,ERV-Sp0
ERV-Spuma.a-Ebu,ERV-Sp0
ERV-Spuma.a-Ebu,ERV-Sp0
ERV-Spuma.a-Ebu,ERV-Sp0
ERV-Spuma.a-Ebu,ERV-Sp0
ERV-Spuma.a-Ebu,ERV-Sp0
ERV-Spuma.a-Ebu,ERV-Sp0
ERV-Spuma.a-Ebu,ERV-Sp0
ERV-Spuma.a-Ebu,ERV-Sp0
ERV-Spuma.a-Ebu,ERV-Sp0
ERV-Spuma.a-Ebu,ERV-Sp0
ERV-Spuma.a-Ebu,ERV-Sp0
ERV-Spuma.a-Ebu,ERV-Sp0
ERV-Spuma.a-Ebu,ERV-Sp0
ERV-Spuma.a-Ebu,ERV-Sp0
ERV-Spuma.a-Ebu,ERV-Sp0
ERV-Spuma.a-Ebu,ERV-Sp0
ERV-Spuma.a-Ebu,ERV-Sp0
ERV-Spuma.a-Ebu,ERV-Sp0
ERV-Spuma.a-Ebu,ERV-Sp0
ERV-Spuma.a-Ebu,ERV-Sp0
ERV-Spuma.a-Ebu,ERV-Sp0
ERV-Spuma.a-Ebu,ERV-Sp0
ERV-Spuma.a-Ebu,ERV-Sp0
ERV-Spuma.a-Ebu,ERV-Sp0
ERV-Spuma.a-Ebu,ERV-Sp0
ERV-Spuma.a-Ebu,ERV-Sp0
ERV-Spuma.a-Ebu,ERV-Sp0
ERV-Spuma.a-Ebu,ERV-Sp0
ERV-Spuma.a-Ebu,ERV-Sp0
ERV-Spuma.a-Ebu,ERV-Sp0
ERV-Spuma.a-Ebu,ERV-Sp0
ERV-Spuma.a-Ebu,ERV-Sp0
ERV-Spuma.a-Ebu,ERV-Sp0
ERV-Spuma.a-Ebu,ERV-Sp0
ERV-Spuma.a-Ebu,ERV-Sp0
ERV-Spuma.a-Ebu,ERV-Sp0
ERV-Spuma.a-Ebu,ERV-Sp0
ERV-Spuma.a-Ebu,ERV-Sp0
ERV-Spuma.a-Ebu,ERV-Sp0
ERV-Spuma.a-Ebu,ERV-Sp0
ERV-Spuma.a-Ebu,ERV-Sp0
ERV-Spuma.a-Ebu,ERV-Sp0
ERV-Spuma.a-Ebu,ERV-Sp0
ERV-Spuma.a-Ebu,ERV-Sp0
ERV-Spuma.a-Ebu,ERV-Sp0
ERV-Spuma.a-Ebu,ERV-Sp0
ERV-Spuma.a-Ebu,ERV-Sp0
ERV-Spuma.a-Ebu,ERV-Sp0
ERV-Spuma.a-Ebu,ERV-Sp0
ERV-Spuma.a-Ebu,ERV-Sp0
ERV-Spuma.a-Ebu,ERV-Sp0
ERV-Spuma.a-Ebu,ERV-Sp0
ERV-Spuma.a-Ebu,ERV-Sp0
ERV-Spuma.a-Ebu,ERV-Sp0
ERV-Spuma.a-Ebu,ERV-Sp0
ERV-Spuma.a-Ebu,ERV-Sp0
ERV-Spuma.a-Ebu,ERV-Sp0
ERV-Spuma.a-Ebu,ERV-Sp0
ERV-Spuma.a-Ebu,ERV-Sp0
ERV-Spuma.a-Ebu,ERV-Sp0
ERV-Spuma.a-Ebu,ERV-Sp0
ERV-Spuma.a-Ebu,ERV-Sp0
ERV-Spuma.a-Ebu,ERV-Sp0
ERV-Spuma.a-Ebu,ERV-Sp0
ERV-Spuma.a-Ebu,ERV-Sp0
ERV-Spuma.a-Ebu,ERV-Sp0
ERV-Spuma.a-Ebu,ERV-Sp0
ERV-Spuma.a-Ebu,ERV-Sp0
ERV-Spuma.a-Ebu,ERV-Sp0
ERV-Spuma.a-Ebu,ERV-Sp0
ERV-Spuma.a-Ebu,ERV-Sp0
ERV-Spuma.a-Ebu,ERV-Sp0
ERV-Spuma.a-Ebu,ERV-Sp0

ERV-Spuma.a-Ebu,ERV-Sp	0
ERV-Spuma.a-Ebu,ERV-Sp	0
ERV-Spuma.a-Ebu,ERV-Sp	0
ERV-Spuma.a-Ebu,ERV-Sp	0
ERV-Spuma.a-Ebu,ERV-Sp	0
ERV-Spuma.a-Ebu,ERV-Sp	0
ERV-Spuma.a-Ebu,ERV-Sp	0
ERV-Spuma.a-Ebu,ERV-Sp	0
ERV-Spuma.a-Ebu,ERV-Sp	0
ERV-Spuma.a-Ebu,ERV-Sp	0
ERV-Spuma.a-Ebu,ERV-Sp	0
ERV-Spuma.a-Ebu,ERV-Sp	0
ERV-Spuma.a-Ebu,ERV-Sp	0
ERV-Spuma.a-Ebu,ERV-Sp	0
ERV-Spuma.a-Ebu,ERV-Sp	0
ERV-Spuma.a-Ebu,ERV-Sp	0
ERV-Spuma.a-Ebu,ERV-Sp	0
ERV-Spuma.a-Ebu,ERV-Sp	0
ERV-Spuma.a-Ebu,ERV-Sp	0
ERV-Spuma.a-Ebu,ERV-Sp	0
ERV-Spuma.a-Ebu,ERV-Sp	0
ERV-Spuma.a-Ebu,ERV-Sp	0
ERV-Spuma.a-Ebu,ERV-Sp	0
ERV-Spuma.a-Ebu,ERV-Sp	0
ERV-Spuma.a-Ebu,ERV-Sp	0
ERV-Spuma.a-Ebu,ERV-Sp	0
ERV-Spuma.a-Ebu,ERV-Sp	0
ERV-Spuma.a-Ebu,ERV-Sp	0
ERV-Spuma.a-Ebu,ERV-Sp	0
ERV-Spuma.a-Ebu,ERV-Sp	0
ERV-Spuma.a-Ebu,ERV-Sp	0
ERV-Spuma.a-Ebu,ERV-Sp	0
ERV-Spuma.a-Ebu,ERV-Sp	0
ERV-Spuma.a-Ebu,ERV-Sp	0
ERV-Spuma.a-Ebu,ERV-Sp	0
ERV-Spuma.a-Ebu,ERV-Sp	0
ERV-Spuma.a-Ebu,ERV-Sp	0
ERV-Spuma.a-Ebu,ERV-Sp	0
ERV-Spuma.a-Ebu,ERV-Sp	0
ERV-Spuma.a-Ebu,ERV-Sp	0
ERV-Spuma.a-Ebu,ERV-Sp	0
ERV-Spuma.a-Ebu,ERV-Sp	0
ERV-Spuma.a-Ebu,ERV-Sp	0
ERV-Spuma.a-Ebu,ERV-Sp	0
ERV-Spuma.a-Ebu,ERV-Sp	0
ERV-Spuma.a-Ebu,ERV-Sp	0
ERV-Spuma.a-Ebu,ERV-Sp	0
ERV-Spuma.a-Ebu,ERV-Sp	0
ERV-Spuma.a-Ebu,ERV-Sp	0
ERV-Spuma.a-Ebu,ERV-Sp	0
ERV-Spuma.a-Ebu,ERV-Sp	0
ERV-Spuma.a-Ebu,ERV-Sp	0
ERV-Spuma.a-Ebu,ERV-Sp	0
ERV-Spuma.a-Ebu,ERV-Sp	0
ERV-Spuma.a-Ebu,ERV-Sp	0
ERV-Spuma.a-Ebu,ERV-Sp	0
ERV-Spuma.a-Ebu,ERV-Sp	0
ERV-Spuma.a-Ebu,ERV-Sp	0
ERV-Spuma.a-Ebu,ERV-Sp	0
ERV-Spuma.a-Ebu,ERV-Sp	0
ERV-Spuma.a-Ebu,ERV-Sp	0
ERV-Spuma.a-Ebu,ERV-Sp	0
ERV-Spuma.a-Ebu,ERV-Sp	0
ERV-Spuma.a-Ebu,ERV-Sp	0
ERV-Spuma.a-Ebu,ERV-Sp	0
ERV-Spuma.a-Ebu,ERV-Sp	0
ERV-Spuma.a-Ebu,ERV-Sp	0
ERV-Spuma.a-Ebu,ERV-Sp	0
ERV-Spuma.a-Ebu,ERV-Sp	0
ERV-Spuma.a-Ebu,ERV-Sp	0
ERV-Spuma.0-Sca,ERV-Sp	0
ERV-Spuma.0-Sca,ERV-Sp	0
ERV-Spuma.0-Sca,ERV-Sp	0
ERV-Spuma.0-Sca,ERV-Sp	0
ERV-Spuma.0-Sca,ERV-Sp	0
ERV-Spuma.0-Sca,ERV-Sp	0
ERV-Spuma.0-Sca,ERV-Sp	0
ERV-Spuma.0-Sca,ERV-Sp	0
ERV-Spuma.0-Sca,ERV-Sp	0
ERV-Spuma.0-Sca,ERV-Sp	0
ERV-Spuma.0-Sca,ERV-Sp	0
ERV-Spuma.0-Sca,ERV-Sp	0
ERV-Spuma.0-Sca,ERV-Sp	0
ERV-Spuma.0-Sca,ERV-Sp	0
ERV-Spuma.0-Sca,ERV-Sp	0
ERV-Spuma.0-Sca,ERV-Sp	0
ERV-Spuma.0-Sca,ERV-Sp	0
ERV-Spuma.0-Sca,ERV-Sp	0
ERV-Spuma.0-Sca,ERV-Sp	0
ERV-Spuma.0-Sca,ERV-Sp	0
ERV-Spuma.0-Sca,ERV-Sp	0
ERV-Spuma.0-Sca,ERV-Sp	0
ERV-Spuma.0-Sca,ERV-Sp	0
ERV-Spuma.0-Sca,ERV-Sp	0
ERV-Spuma.0-Sca,ERV-Sp	0
ERV-Spuma.0-Sca,ERV-Sp	0
ERV-Spuma.0-Sca,ERV-Sp	0
ERV-Spuma.0-Sca,ERV-Sp	0
ERV-Spuma.0-Sca,ERV-Sp	0
ERV-Spuma.0-Sca,ERV-Sp	0
ERV-Spuma.0-Sca,ERV-Sp	0
ERV-Spuma.0-Sca,ERV-Sp	0
ERV-Spuma.0-Sca,ERV-Sp	0
ERV-Spuma.0-Sca,ERV-Sp	0
ERV-Spuma.0-Sca,ERV-Sp	0
ERV-Spuma.0-Sca,ERV-Sp	0
ERV-Spuma.0-Sca,ERV-Sp	0
ERV-Spuma.0-Sca,ERV-Sp	0
ERV-Spuma.0-Sca,ERV-Sp	0
ERV-Spuma.0-Sca,ERV-Sp	0
ERV-Spuma.0-Sca,ERV-Sp	0
ERV-Spuma.0-Sca,ERV-Sp	0
ERV-Spuma.0-Sca,ERV-Sp	0
ERV-Spuma.0-Sca,ERV-Sp	0
ERV-Spuma.0-Sca,ERV-Sp	0
ERV-Spuma.0-Sca,ERV-Sp	0
ERV-Spuma.0-Sca,ERV-Sp	0
ERV-Spuma.0-Sca,ERV-Sp	0
ERV-Spuma.0-Sca,ERV-Sp	0
ERV-Spuma.0-Sca,ERV-Sp	0
ERV-Spuma.0-Sca,ERV-Sp	0
ERV-Spuma.0-Sca,ERV-Sp	0
ERV-Spuma.0-Sca,ERV-Sp	0
ERV-Spuma.0-Sca,ERV-Sp	0
ERV-Spuma.0-Sca,ERV-Sp	0
ERV-Spuma.0-Sca,ERV-Sp	0
ERV-Spuma.0-Sca,ERV-Sp	0
ERV-Spuma.0-Sca,ERV-Sp	0
ERV-Spuma.0-Sca,ERV-Sp	0
ERV-Spuma.0-Sca,ERV-Sp	0
ERV-Spuma.0-Sca,ERV-Sp	0
ERV-Spuma.0-Sca,ERV-Sp	0
ERV-Spuma.0-Sca,ERV-Sp	0
ERV-Spuma.0-Sca	0
ERV-Spuma.0-Pma,ERV-S[	0
ERV-Spuma.0-Pma,ERV-S[	0
ERV-Spuma.0-Pma,ERV-S[	0
ERV-Spuma.0-Pma,ERV-S[	0
ERV-Spuma.0-Pma,ERV-S[	0
ERV-Spuma.0-Pma,ERV-S[	0
ERV-Spuma.0-Pma,ERV-S[	0
ERV-Spuma.0-Pma,ERV-S[	0

ROWS_PLACEHOLDER

ERV-Spuma.0-Lca,ERV-Sp	0
ERV-Spuma.0-Lca,ERV-Sp	0
ERV-Spuma.0-Lca,ERV-Sp	0
ERV-Spuma.0-Lca,ERV-Sp	0
ERV-Spuma.0-Lca,ERV-Sp	0
ERV-Spuma.0-Lca,ERV-Sp	0
ERV-Spuma.0-Lca,ERV-Sp	0
ERV-Spuma.0-Lca,ERV-Sp	0
ERV-Spuma.0-Lca,ERV-Sp	0
ERV-Spuma.0-Lca,ERV-Sp	0
ERV-Spuma.0-Lca,ERV-Sp	0
ERV-Spuma.0-Lca,ERV-Sp	0
ERV-Spuma.0-Lca,ERV-Sp	0
ERV-Spuma.0-Lca,ERV-Sp	0
ERV-Spuma.0-Lca,ERV-Sp	0
ERV-Spuma.0-Lca,ERV-Sp	0
ERV-Spuma.0-Lca,ERV-Sp	0
ERV-Spuma.0-Lca,ERV-Sp	0
ERV-Spuma.0-Lca,ERV-Sp	0
ERV-Spuma.0-Lca,ERV-Sp	0
ERV-Spuma.0-Lca,ERV-Sp	0
ERV-Spuma.0-Lca	0
ERV-Epcilon.0-Ebu,ERV-S	0
ERV-Epcilon.0-Ebu,ERV-S	0
ERV-Epcilon.0-Ebu,ERV-S	0
ERV-Epcilon.0-Ebu,ERV-S	0
ERV-Epcilon.0-Ebu,ERV-S	0
ERV-Epcilon.0-Ebu,ERV-S	0
ERV-Epcilon.0-Ebu,ERV-S	0
ERV-Epcilon.0-Ebu,ERV-S	0
ERV-Epcilon.0-Ebu,ERV-S	0
ERV-Epcilon.0-Ebu,ERV-S	0
ERV-Epcilon.0-Ebu,ERV-S	0
ERV-Epcilon.0-Ebu,ERV-S	0
ERV-Epcilon.0-Ebu,ERV-S	0
ERV-Epcilon.0-Ebu,ERV-S	0
ERV-Epcilon.0-Ebu,ERV-S	0
ERV-Epcilon.0-Ebu,ERV-S	0
ERV-Epcilon.0-Ebu,ERV-S	0
ERV-Epcilon.0-Ebu,ERV-S	0
ERV-Epcilon.0-Ebu,ERV-S	0
ERV-Epcilon.0-Ebu,ERV-S	0
ERV-Epcilon.0-Ebu,ERV-S	0
ERV-Epcilon.0-Ebu,ERV-S	0
ERV-Epcilon.0-Ebu,ERV-S	0
ERV-Epcilon.0-Ebu,ERV-S	0
ERV-Epcilon.0-Ebu,ERV-S	0
ERV-Epcilon.0-Ebu,ERV-S	0
ERV-Epcilon.0-Ebu,ERV-S	0
ERV-Epcilon.0-Ebu,ERV-S	0
ERV-Epcilon.0-Ebu,ERV-S	0
ERV-Epcilon.0-Ebu,ERV-S	0
ERV-Epcilon.0-Ebu,ERV-S	0
ERV-Epcilon.0-Ebu,ERV-S	0
ERV-Epcilon.0-Ebu,ERV-S	0
ERV-Epcilon.0-Ebu,ERV-S	0
ERV-Epcilon.0-Ebu,ERV-S	0
ERV-Epcilon.0-Ebu,ERV-S	0
ERV-Epcilon.0-Ebu,ERV-S	0
ERV-Epcilon.0-Ebu,ERV-S	0
ERV-Epcilon.0-Ebu,ERV-S	0
ERV-Epcilon.0-Ebu,ERV-S	0
ERV-Epcilon.0-Ebu,ERV-S	0
ERV-Epcilon.0-Ebu,ERV-S	0
ERV-Epcilon.0-Ebu,ERV-S	0
ERV-Epcilon.0-Ebu,ERV-S	0
ERV-Epcilon.0-Ebu,ERV-S	0
ERV-Epcilon.0-Ebu,ERV-S	0
ERV-Epcilon.0-Ebu,ERV-S	0
ERV-Epcilon.0-Ebu,ERV-S	0
ERV-Epcilon.0-Ebu,ERV-S	0
ERV-Epcilon.0-Ebu,ERV-S	0
ERV-Epcilon.0-Ebu,ERV-S	0
ERV-Epcilon.0-Ebu,ERV-S	0
ERV-Epcilon.0-Ebu,ERV-S	0
ERV-Epcilon.0-Ebu,ERV-S	0
ERV-Epcilon.0-Ebu,ERV-S	0
ERV-Epcilon.0-Ebu,ERV-S	0
ERV-Epcilon.0-Ebu,ERV-S	0
ERV-Epcilon.0-Ebu,ERV-S	0
ERV-Epcilon.0-Ebu,ERV-S	0
ERV-Epcilon.0-Ebu,ERV-S	0
ERV-Epcilon.0-Ebu,ERV-S	0
ERV-Epcilon.0-Ebu,ERV-S	0
ERV-Epcilon.0-Ebu,ERV-S	0
ERV-Epcilon.0-Ebu,ERV-S	0
ERV-Epcilon.0-Ebu,ERV-S	0
ERV-Epcilon.0-Ebu,ERV-S	0
ERV-Epcilon.0-Ebu,ERV-S	0
ERV-Epcilon.0-Ebu,ERV-S	0
ERV-Epcilon.0-Ebu,ERV-S	0
ERV-Epcilon.0-Ebu,ERV-S	0
ERV-Epcilon.0-Ebu,ERV-S	0
ERV-Epcilon.0-Ebu,ERV-S	0
ERV-Epcilon.0-Ebu,ERV-S	0
ERV-Epcilon.0-Ebu,ERV-S	0
ERV-Epcilon.0-Ebu,ERV-S	0
ERV-Epcilon.0-Ebu,ERV-S	0
ERV-Epcilon.0-Ebu,ERV-S	0
ERV-Epcilon.0-Ebu,ERV-S	0
ERV-Epcilon.0-Ebu,ERV-S	0
ERV-Epcilon.0-Ebu,ERV-S	0
ERV-Epcilon.0-Ebu,ERV-S	0
ERV-Epcilon.0-Ebu,ERV-S	0
ERV-Epcilon.0-Ebu,ERV-S	0
ERV-Epcilon.0-Ebu,ERV-S	0
ERV-Epcilon.0-Ebu,ERV-S	0
ERV-Epcilon.0-Ebu,ERV-S	0
ERV-Epcilon.0-Ebu,ERV-S	0
ERV-Epcilon.0-Ebu,ERV-S	0
ERV-Epcilon.0-Ebu,ERV-S	0
ERV-Epcilon.0-Ebu,ERV-S	0
ERV-Epcilon.0-Ebu,ERV-S	0
ERV-Epcilon.0-Ebu,ERV-S	0
ERV-Epcilon.0-Ebu,ERV-S	0
ERV-Epcilon.0-Ebu,ERV-S	0
ERV-Epcilon.0-Ebu,ERV-S	0
ERV-Epcilon.0-Ebu,ERV-S	0
ERV-Epcilon.0-Ebu,ERV-S	0
ERV-Epcilon.0-Ebu,ERV-S	0
ERV-Epcilon.0-Ebu,ERV-S	0
ERV-Epcilon.0-Ebu,ERV-S	0
ERV-Epcilon.0-Ebu,ERV-S	0
ERV-Epcilon.0-Ebu,ERV-S	0
ERV-Epcilon.0-Ebu,ERV-S	0
ERV-Epcilon.0-Ebu,ERV-S	0
ERV-Epcilon.0-Ebu,ERV-S	0
ERV-Epcilon.0-Ebu,ERV-S	0
ERV-Epcilon.0-Ebu,ERV-S	0
ERV-Epcilon.0-Ebu,ERV-S	0
ERV-Epcilon.0-Ebu,ERV-S	0
ERV-Epcilon.0-Ebu,ERV-S	0
ERV-Epcilon.0-Ebu,ERV-S	0
ERV-Epcilon.0-Ebu,ERV-S	0
ERV-Epcilon.0-Ebu,ERV-S	0
ERV-Epcilon.0-Ebu,ERV-S	0
ERV-Epcilon.0-Ebu,ERV-S	0
ERV-Epcilon.0-Ebu,ERV-S	0
ERV-Epcilon.0-Ebu,ERV-S	0
ERV-Epcilon.0-Ebu,ERV-S	0
ERV-Epcilon.0-Ebu,ERV-S	0
ERV-Epcilon.0-Ebu,ERV-S	0
ERV-Epcilon.0-Ebu,ERV-S	0

ERV-Epcilon.0-Ebu,ERV-S	0
ERV-Epcilon.0-Ebu,ERV-S	0
ERV-Epcilon.0-Ebu,ERV-S	0
ERV-Epcilon.0-Ebu,ERV-S	0
ERV-Epcilon.0-Ebu,ERV-S	0
ERV-Epcilon.0-Ebu,ERV-S	0
ERV-Epcilon.0-Ebu,ERV-S	0
ERV-Epcilon.0-Ebu,ERV-S	0
ERV-Epcilon.0-Ebu,ERV-S	0
ERV-Epcilon.0-Ebu,ERV-S	0
ERV-Epcilon.0-Ebu,ERV-S	0
ERV-Epcilon.0-Ebu,ERV-S	0
ERV-Epcilon.0-Ebu,ERV-S	0
ERV-Epcilon.0-Ebu,ERV-S	0
ERV-Epcilon.0-Ebu,ERV-S	0
ERV-Epcilon.0-Ebu,ERV-S	0
ERV-Epcilon.0-Ebu,ERV-S	0
ERV-Epcilon.0-Ebu,ERV-S	0
ERV-Epcilon.0-Ebu,ERV-S	0
ERV-Epcilon.0-Ebu,ERV-S	0
ERV-Epcilon.0-Ebu,ERV-S	0
ERV-Epcilon.0-Ebu,ERV-S	0
ERV-Epcilon.0-Ebu,ERV-S	0
ERV-Epcilon.0-Ebu,ERV-S	0
ERV-Epcilon.0-Ebu,ERV-S	0
ERV-Epcilon.0-Ebu,ERV-S	0
ERV-Epcilon.0-Ebu,ERV-S	0
ERV-Epcilon.0-Ebu,ERV-S	0
ERV-Epcilon.0-Ebu,ERV-S	0
ERV-Epcilon.0-Ebu,ERV-S	0
ERV-Epcilon.0-Ebu,ERV-S	0
ERV-Epcilon.0-Ebu,ERV-S	0
ERV-Epcilon.0-Ebu,ERV-S	0
ERV-Epcilon.0-Ebu,ERV-S	0
ERV-Epcilon.0-Ebu,ERV-S	0
ERV-Epcilon.0-Ebu,ERV-S	0
ERV-Epcilon.0-Ebu,ERV-S	0
ERV-Epcilon.0-Ebu,ERV-S	0
ERV-Epcilon.0-Ebu,ERV-S	0
ERV-Epcilon.0-Ebu,ERV-S	0
ERV-Epcilon.0-Ebu,ERV-S	0
ERV-Epcilon.0-Ebu,ERV-S	0
ERV-Epcilon.0-Ebu,ERV-S	0
ERV-Epcilon.0-Ebu,ERV-S	0
ERV-Epcilon.0-Ebu,ERV-S	0
ERV-Epcilon.0-Ebu,ERV-S	0
ERV-Epcilon.0-Ebu,ERV-S	0
ERV-Epcilon.0-Ebu,ERV-S	0
ERV-Epcilon.0-Ebu,ERV-S	0
ERV-Epcilon.0-Ebu,ERV-S	0
ERV-Epcilon.0-Ebu,ERV-S	0
ERV-Epcilon.0-Ebu,ERV-S	0
ERV-Epcilon.0-Ebu,ERV-S	0
ERV-Epcilon.0-Ebu,ERV-S	0
ERV-Epcilon.0-Ebu,ERV-S	0
ERV-Epcilon.0-Ebu,ERV-S	0
ERV-Epcilon.0-Ebu,ERV-S	0
ERV-Epcilon.0-Ebu,ERV-S	0
ERV-Epcilon.0-Ebu,ERV-S	0
ERV-Epcilon.0-Ebu,ERV-S	0
ERV-Epcilon.0-Ebu,ERV-S	0
ERV-Epcilon.0-Ebu,ERV-S	0
ERV-Epcilon.0-Ebu,ERV-S	0
ERV-Epcilon.0-Ebu,ERV-S	0
ERV-Epcilon.0-Ebu,ERV-S	0
ERV-Epcilon.0-Ebu,ERV-S	0
ERV-Epcilon.0-Ebu,ERV-S	0
ERV-Epcilon.0-Ebu,ERV-S	0
ERV-Epcilon.0-Ebu,ERV-S	0
ERV-Epcilon.0-Ebu,ERV-S	0
ERV-Epcilon.0-Ebu,ERV-S	0
ERV-Epcilon.0-Ebu,ERV-S	0
ERV-Epcilon.0-Ebu,ERV-S	0
ERV-Epcilon.0-Ebu,ERV-S	0
ERV-Epcilon.0-Ebu,ERV-S	0
ERV-Epcilon.0-Ebu,ERV-S	0
ERV-Epcilon.0-Ebu,ERV-S	0
ERV-Epcilon.0-Ebu,ERV-S	0
ERV-Epcilon.0-Ebu,ERV-S	0
ERV-Epcilon.0-Ebu,ERV-S	0
ERV-Epcilon.0-Ebu,ERV-S	0
ERV-Epcilon.0-Ebu,ERV-S	0
ERV-Epcilon.0-Ebu,ERV-S	0
ERV-Epcilon.0-Ebu,ERV-S	0
ERV-Epcilon.0-Ebu,ERV-S	0
ERV-Epcilon.0-Ebu,ERV-S	0
ERV-Epcilon.0-Ebu,ERV-S	0
ERV-Epcilon.0-Ebu,ERV-S	0
ERV-Epcilon.0-Ebu,ERV-S	0
ERV-Epcilon.0-Ebu,ERV-S	0
ERV-Epcilon.0-Ebu,ERV-S	0
ERV-Epcilon.0-Ebu,ERV-S	0
ERV-Epcilon.0-Ebu,ERV-S	0
ERV-Epcilon.0-Ebu,ERV-S	0
ERV-Epcilon.0-Ebu,ERV-S	0
ERV-Epcilon.0-Ebu,ERV-S	0
ERV-Epcilon.0-Ebu,ERV-S	0
ERV-Epcilon.0-Ebu,ERV-S	0
ERV-Epcilon.0-Ebu,ERV-S	0
ERV-Epcilon.0-Ebu,ERV-S	0
ERV-Epcilon.0-Ebu,ERV-S	0
ERV-Epcilon.0-Ebu,ERV-S	0
ERV-Epcilon.0-Ebu,ERV-S	0
ERV-Epcilon.0-Ebu,ERV-S	0
ERV-Epcilon.0-Ebu,ERV-S	0
ERV-Epcilon.0-Ebu,ERV-S	0
ERV-Epcilon.0-Ebu,ERV-S	0
ERV-Epcilon.0-Ebu,ERV-S	0
ERV-Epcilon.0-Ebu,ERV-S	0
ERV-Epcilon.0-Ebu,ERV-S	0
ERV-Epcilon.0-Ebu,ERV-S	0
ERV-Epcilon.0-Ebu,ERV-S	0
ERV-Epcilon.0-Ebu,ERV-S	0
ERV-Epcilon.0-Ebu,ERV-S	0
ERV-Epcilon.0-Ebu,ERV-S	0
ERV-Epcilon.0-Ebu,ERV-S	0
ERV-Epcilon.0-Ebu,ERV-S	0
ERV-Epcilon.0-Ebu,ERV-S	0
ERV-Epcilon.0-Ebu,ERV-S	0
ERV-Epcilon.0-Ebu,ERV-S	0
ERV-Epcilon.0-Ebu,ERV-S	0
ERV-Epcilon.0-Ebu,ERV-S	0
ERV-Epcilon.0-Ebu,ERV-S	0
ERV-Epcilon.0-Ebu,ERV-S	0
ERV-Epcilon.0-Ebu,ERV-S	0
ERV-Epcilon.0-Ebu,ERV-S	0
ERV-Epcilon.0-Ebu,ERV-S	0
ERV-Epcilon.0-Ebu,ERV-S	0
ERV-Epcilon.0-Ebu,ERV-S	0
ERV-Epcilon.0-Ebu,ERV-S	0
ERV-Epcilon.0-Ebu,ERV-S	0
ERV-Epcilon.0-Ebu,ERV-S	0
ERV-Epcilon.0-Ebu,ERV-S	0
ERV-Epcilon.0-Ebu,ERV-S	0
ERV-Epcilon.0-Ebu,ERV-S	0
ERV-Epcilon.0-Ebu,ERV-S	0
ERV-Epcilon.0-Ebu,ERV-S	0
ERV-Epcilon.0-Ebu,ERV-S	0
ERV-Epcilon.0-Ebu,ERV-S	0
ERV-Epcilon.0-Ebu,ERV-S	0
ERV-Epcilon.0-Ebu,ERV-S	0
ERV-Epcilon.0-Ebu,ERV-S	0

ERV-Epcilon.0-Ebu,ERV-S|0
ERV-Epcilon.0-Ebu,ERV-S|0
ERV-Epcilon.0-Ebu,ERV-S|0
ERV-Epcilon.0-Ebu,ERV-S|0
ERV-Epcilon.0-Ebu,ERV-S|0
ERV-Epcilon.0-Ebu,ERV-S|0
ERV-Epcilon.0-Ebu,ERV-S|0
ERV-Epcilon.0-Ebu,ERV-S|0
ERV-Epcilon.0-Ebu,ERV-S|0
ERV-Epcilon.0-Ebu,ERV-S|0
ERV-Epcilon.0-Ebu,ERV-S|0
ERV-Epcilon.0-Ebu,ERV-S|0
ERV-Epcilon.0-Ebu,ERV-S|0
ERV-Epcilon.0-Ebu,ERV-S|0
ERV-Epcilon.0-Ebu,ERV-S|0
ERV-Epcilon.0-Ebu,ERV-S|0
ERV-Epcilon.0-Ebu,ERV-S|0
ERV-Epcilon.0-Ebu,ERV-S|0
ERV-Epcilon.0-Ebu,ERV-S|0
ERV-Epcilon.0-Ebu,ERV-S|0
ERV-Epcilon.0-Ebu,ERV-S|0
ERV-Epcilon.0-Ebu,ERV-S|0
ERV-Epcilon.0-Ebu,ERV-S|0
ERV-Epcilon.0-Ebu,ERV-S|0
ERV-Epcilon.0-Ebu,ERV-S|0
ERV-Epcilon.0-Ebu,ERV-S|0
ERV-Epcilon.0-Ebu,ERV-S|0
ERV-Epcilon.0-Ebu,ERV-S|0
ERV-Epcilon.0-Ebu,ERV-S|0
ERV-Epcilon.0-Ebu,ERV-S|0
ERV-Epcilon.0-Ebu,ERV-S|0
ERV-Epcilon.0-Ebu,ERV-S|0
ERV-Epcilon.0-Ebu,ERV-S|0
ERV-Epcilon.0-Ebu,ERV-S|0
ERV-Epcilon.0-Ebu,ERV-S|0
ERV-Epcilon.0-Ebu,ERV-S|0
ERV-Epcilon.0-Ebu,ERV-S|0
ERV-Epcilon.0-Ebu,ERV-S|0
ERV-Epcilon.0-Ebu,ERV-S|0
ERV-Epcilon.0-Ebu,ERV-S|0
ERV-Epcilon.0-Ebu,ERV-S|0
ERV-Epcilon.0-Ebu,ERV-S|0
ERV-Epcilon.0-Ebu,ERV-S|0
ERV-Epcilon.0-Ebu,ERV-S|0
ERV-Epcilon.0-Ebu,ERV-S|0
ERV-Epcilon.0-Ebu,ERV-S|0
ERV-Epcilon.0-Ebu,ERV-S|0
ERV-Epcilon.0-Ebu,ERV-S|0
ERV-Epcilon.0-Ebu,ERV-S|0
ERV-Epcilon.0-Ebu,ERV-S|0
ERV-Epcilon.0-Ebu,ERV-S|0
ERV-Epcilon.0-Ebu,ERV-S|0
ERV-Epcilon.0-Ebu,ERV-S|0
ERV-Epcilon.0-Ebu,ERV-S|0
ERV-Epcilon.0-Ebu,ERV-S|0
ERV-Epcilon.0-Ebu,ERV-S|0
ERV-Epcilon.0-Ebu,ERV-S|0
ERV-Epcilon.0-Ebu,ERV-S|0
ERV-Epcilon.0-Ebu,ERV-S|0
ERV-Epcilon.0-Ebu,ERV-S|0
ERV-Epcilon.0-Ebu,ERV-S|0
ERV-Epcilon.0-Ebu,ERV-S|0
ERV-Epcilon.0-Ebu,ERV-S|0
ERV-Epcilon.0-Ebu,ERV-S|0
ERV-Epcilon.0-Ebu,ERV-S|0
ERV-Epcilon.0-Ebu,ERV-S|0
ERV-Epcilon.0-Ebu,ERV-S|0
ERV-Epcilon.0-Ebu,ERV-S|0
ERV-Epcilon.0-Ebu,ERV-S|0
ERV-Epcilon.0-Ebu,ERV-S|0
ERV-Epcilon.0-Ebu,ERV-S|0
ERV-Epcilon.0-Ebu,ERV-S|0
ERV-Epcilon.0-Ebu,ERV-S|0
ERV-Epcilon.0-Ebu,ERV-S|0
ERV-Epcilon.0-Ebu,ERV-S|0
ERV-Epcilon.0-Ebu,ERV-S|0
ERV-Epcilon.0-Ebu,ERV-S|0
ERV-Epcilon.0-Ebu,ERV-S|0
ERV-Epcilon.0-Ebu,ERV-S|0
ERV-Epcilon.0-Ebu,ERV-S|0
ERV-Epcilon.0-Ebu,ERV-S|0
ERV-Epcilon.0-Ebu,ERV-S|0
ERV-Epcilon.0-Ebu,ERV-S|0
ERV-Epcilon.0-Ebu,ERV-S|0
ERV-Epcilon.0-Ebu,ERV-S|0
ERV-Epcilon.0-Ebu,ERV-S|0
ERV-Epcilon.0-Ebu,ERV-S|0
ERV-Epcilon.0-Ebu,ERV-S|0
ERV-Epcilon.0-Ebu,ERV-S|0
ERV-Epcilon.0-Ebu,ERV-S|0
ERV-Epcilon.0-Ebu,ERV-S|0
ERV-Epcilon.0-Ebu,ERV-S|0
ERV-Epcilon.0-Ebu,ERV-S|0
ERV-Epcilon.0-Ebu,ERV-S|0
ERV-Epcilon.0-Ebu,ERV-S|0
ERV-Epcilon.0-Ebu,ERV-S|0
ERV-Epcilon.0-Ebu,ERV-S|0
ERV-Epcilon.0-Ebu,ERV-S|0
ERV-Epcilon.0-Ebu,ERV-S|0
ERV-Epcilon.0-Ebu,ERV-S|0
ERV-Epcilon.0-Ebu,ERV-S|0
ERV-Epcilon.0-Ebu,ERV-S|0
ERV-Epcilon.0-Ebu,ERV-S|0
ERV-Epcilon.0-Ebu,ERV-S|0
ERV-Epcilon.0-Ebu,ERV-S|0
ERV-Epcilon.0-Ebu,ERV-S|0
ERV-Epcilon.0-Ebu,ERV-S|0
ERV-Epcilon.0-Ebu,ERV-S|0
ERV-Epcilon.0-Ebu,ERV-S|0
ERV-Epcilon.0-Ebu,ERV-S|0
ERV-Epcilon.0-Ebu,ERV-S|0
ERV-Epcilon.0-Ebu,ERV-S|0
ERV-Epcilon.0-Ebu,ERV-S|0
ERV-Epcilon.0-Ebu,ERV-S|0
ERV-Epcilon.0-Ebu,ERV-S|0
ERV-Epcilon.0-Ebu,ERV-S|0
ERV-Epcilon.0-Ebu,ERV-S|0
ERV-Epcilon.0-Ebu,ERV-S|0
ERV-Epcilon.0-Ebu,ERV-S|0
ERV-Epcilon.0-Ebu,ERV-S|0
ERV-Epcilon.0-Ebu,ERV-S|0
ERV-Epcilon.0-Ebu,ERV-S|0
ERV-Epcilon.0-Ebu,ERV-S|0
ERV-Epcilon.0-Ebu,ERV-S|0
ERV-Epcilon.0-Ebu,ERV-S|0
ERV-Epcilon.0-Ebu,ERV-S|0
ERV-Epcilon.0-Ebu,ERV-S|0
ERV-Epcilon.0-Ebu,ERV-S|0
ERV-Epcilon.0-Ebu,ERV-S|0
ERV-Epcilon.0-Ebu,ERV-S|0
ERV-Epcilon.0-Ebu,ERV-S|0
ERV-Epcilon.0-Ebu,ERV-S|0
ERV-Epcilon.0-Ebu,ERV-S|0
ERV-Epcilon.0-Ebu,ERV-S|0
ERV-Epcilon.0-Ebu,ERV-S|0
ERV-Epcilon.0-Ebu,ERV-S|0
ERV-Epcilon.0-Ebu,ERV-S|0
ERV-Epcilon.0-Ebu,ERV-S|0
ERV-Epcilon.0-Ebu,ERV-S|0
ERV-Epcilon.0-Ebu,ERV-S|0
ERV-Epcilon.0-Ebu,ERV-S|0
ERV-Epcilon.0-Ebu,ERV-S|0

ERV-Epcilon.0-Ebu,ERV-S	0
ERV-Epcilon.0-Ebu,ERV-S	0
ERV-Epcilon.0-Ebu,ERV-S	0
ERV-Epcilon.0-Ebu,ERV-S	0
ERV-Epcilon.0-Ebu,ERV-S	0
ERV-Epcilon.0-Ebu,ERV-S	0
ERV-Epcilon.0-Ebu,ERV-S	0
ERV-Epcilon.0-Ebu,ERV-S	0
ERV-Epcilon.0-Ebu,ERV-S	0
ERV-Epcilon.0-Ebu,ERV-S	0
ERV-Epcilon.0-Ebu,ERV-S	0
ERV-Epcilon.0-Ebu,ERV-S	0
ERV-Epcilon.0-Ebu,ERV-S	0
ERV-Epcilon.0-Ebu,ERV-S	0
ERV-Epcilon.0-Ebu,ERV-S	0
ERV-Epcilon.0-Ebu,ERV-S	0
ERV-Epcilon.0-Ebu,ERV-S	0
ERV-Epcilon.0-Ebu,ERV-S	0
ERV-Epcilon.0-Ebu,ERV-S	0
ERV-Epcilon.0-Ebu,ERV-S	0
ERV-Epcilon.0-Ebu,ERV-S	0
ERV-Epcilon.0-Ebu,ERV-S	0
ERV-Epcilon.0-Ebu,ERV-S	0
ERV-Epcilon.0-Ebu,ERV-S	0
ERV-Epcilon.0-Ebu,ERV-S	0
ERV-Epcilon.0-Ebu,ERV-S	0
ERV-Epcilon.0-Ebu,ERV-S	0
ERV-Epcilon.0-Ebu,ERV-S	0
ERV-Epcilon.0-Ebu,ERV-S	0
ERV-Epcilon.0-Ebu,ERV-S	0
ERV-Epcilon.0-Ebu,ERV-S	0
ERV-Epcilon.0-Ebu,ERV-S	0
ERV-Epcilon.0-Ebu,ERV-S	0
ERV-Epcilon.0-Ebu,ERV-S	0
ERV-Epcilon.0-Ebu,ERV-S	0
ERV-Epcilon.0-Ebu,ERV-S	0
ERV-Epcilon.0-Ebu,ERV-S	0
ERV-Epcilon.0-Ebu,ERV-S	0
ERV-Epcilon.0-Ebu,ERV-S	0
ERV-Epcilon.0-Ebu,ERV-S	0
ERV-Epcilon.0-Ebu,ERV-S	0
ERV-Epcilon.0-Ebu,ERV-S	0
ERV-Epcilon.0-Ebu,ERV-S	0
ERV-Epcilon.0-Ebu,ERV-S	0
ERV-Epcilon.0-Ebu,ERV-S	0
ERV-Epcilon.0-Ebu,ERV-S	0
ERV-Epcilon.0-Ebu,ERV-S	0
ERV-Epcilon.0-Ebu,ERV-S	0
ERV-Epcilon.0-Ebu,ERV-S	0
ERV-Epcilon.0-Ebu,ERV-S	0
ERV-Epcilon.0-Ebu,ERV-S	0
ERV-Epcilon.0-Ebu,ERV-S	0
ERV-Epcilon.0-Ebu,ERV-S	0
ERV-Epcilon.0-Ebu,ERV-S	0
ERV-Epcilon.0-Ebu,ERV-S	0
ERV-Epcilon.0-Ebu,ERV-S	0
ERV-Epcilon.0-Ebu,ERV-S	0
ERV-Epcilon.0-Ebu,ERV-S	0
ERV-Epcilon.0-Ebu,ERV-S	0
ERV-Epcilon.0-Ebu,ERV-S	0
ERV-Epcilon.0-Ebu,ERV-S	0
ERV-Epcilon.0-Ebu,ERV-S	0
ERV-Epcilon.0-Ebu,ERV-S	0
ERV-Epcilon.0-Ebu,ERV-S	0
ERV-Epcilon.0-Ebu,ERV-S	0
ERV-Epcilon.0-Ebu,ERV-S	0
ERV-Epcilon.0-Ebu,ERV-S	0
ERV-Epcilon.0-Ebu,ERV-S	0
ERV-Epcilon.0-Ebu,ERV-S	0
ERV-Epcilon.0-Ebu,ERV-S	0
ERV-Epcilon.0-Ebu,ERV-S	0
ERV-Epcilon.0-Ebu,ERV-S	0
ERV-Epcilon.0-Ebu,ERV-S	0
ERV-Epcilon.0-Ebu,ERV-S	0
ERV-Epcilon.0-Ebu,ERV-S	0
ERV-Epcilon.0-Ebu,ERV-S	0
ERV-Epcilon.0-Ebu,ERV-S	0
ERV-Epcilon.0-Ebu,ERV-S	0
ERV-Epcilon.0-Ebu,ERV-S	0
ERV-Epcilon.0-Ebu,ERV-S	0
ERV-Epcilon.0-Ebu,ERV-S	0
ERV-Epcilon.0-Ebu,ERV-S	0
ERV-Epcilon.0-Ebu,ERV-S	0
ERV-Epcilon.0-Ebu,ERV-S	0
ERV-Epcilon.0-Ebu,ERV-S	0
ERV-Epcilon.0-Ebu,ERV-S	0
ERV-Epcilon.0-Ebu,ERV-S	0
ERV-Epcilon.0-Ebu,ERV-S	0
ERV-Epcilon.0-Ebu,ERV-S	0
ERV-Epcilon.0-Ebu,ERV-S	0
ERV-Epcilon.0-Ebu,ERV-S	0
ERV-Epcilon.0-Ebu,ERV-S	0
ERV-Epcilon.0-Ebu,ERV-S	0
ERV-Epcilon.0-Ebu,ERV-S	0
ERV-Epcilon.0-Ebu,ERV-S	0
ERV-Epcilon.0-Ebu,ERV-S	0

Supplementary Table S6. The representative retrovirus sequences used for phylogenetic reconstruction

Name	Accession No./Source
Bovine foamy virus (BFV)	NC 001831.1
Equine foamy virus (EFV)	NC_002201.1
Feline foamy virus (FFV)	NC_001871.1
SFVspm	EU010385.1
SFVgor	HM245790.1
SFVcpz	NC_001364.1
SFVmac	NC_010819.1
SFVagm	NC_010820.1
Bovine leukemia virus (BLV)	NP_056895.1
Human T-cell leukemia virus type 1 (HTLV-1)	NP_057860.1
Human T-cell leukemia virus type 2 (HTLV-2)	NP_041003.2
Bovine immunodeficiency virus (BIV)	NP_040563.1
Human immunodeficiency virus type 2 (HIV-2)	NP_663784.1
Human immunodeficiency virus type 1 (HIV-1)	ABK51636.1
SIVcol	AAK01033.1
SIVcpz	ABU53017.1
Jembrana disease virus (JDV)	AAA64389.1
Caprine arthritis-encephalitis virus (CAEV)	NP_040939.1
Maedi-visna virus (MVV)	YP_536867.1
Equine infectious anemia virus (EIAV)	NP_056902.1
Lymphoproliferative disease virus (LDV)	AAA62195.1
Avian leukosis virus (ALV)	YP_004222728.1
Python molurus endogenous retrovirus (Python-molurus)	AAN77283.1
Mouse mammary tumor virus (MMTV)	NP_056880.1
Jaagsiekte sheep retrovirus (JSRV)	NP_041186.1
Simian retrovirus 2 (SRV-2)	AAA47562.1
Simian retrovirus 1 (SRV-1)	AAA47732.1
Snakehead retrovirus (SnRV)	NP_043924.1
Walleye epidermal hyperplasia virus type 1 (WEHV-1)	AF133051
Walleye epidermal hyperplasia virus type 2 (WEHV-2)	AF133052
Walleye dermal sarcoma virus (WDSV)	NP_045937.1
Feline leukemia virus (FLV)	NP_955577.1
Moloney murine leukemia virus (MMLV)	NP_057933.2
Baboon endogenous retrovirus (ERV-Baboon)	BAA89659.1
Gibbon ape leukemia virus (GALV)	NP_056790.1
RD114 retrovirus	NC_009889
Rhinolophus ferrumequinum retrovirus (RfRV)	JQ303225
CmiFLERV	Ref. 1
Porcine endogenous retrovirus C (PERV-A)	EU789636
Porcine endogenous retrovirus C (PERV-C)	HM159246
Xenopus tropicalis endogenous retrovirus 1 (XTERV1)	HM765512
Zebrafish endogenous retrovirus (ZFERV)	AF503912
Atlantic salmon swim bladder sarcoma virus (SSSV)	DQ174103
Sloth endogenous foamy virus (SloEFV)	Ref. 2
HERV-Fc1	Ref. 3
HERV-E	Ref. 3
Xen1	Ref. 3
Coelacanth endogenous foamy virus (CoEFV)	Ref. 4
CmiERV	Ref. 5

References

Ref. 1	Aiewsakun, P. and A. Katzourakis (2017). "Marine origin of retroviruses in the early Palaeozoic Era." Nat Commun 8: 13954.
Ref. 2	Katzourakis, A., et al. (2009). "Macroevolution of complex retroviruses." Science 325(5947): 1512.
Ref. 3	Jern, P., et al. (2005). "Use of endogenous retroviral sequences (ERVs) and structural markers for retroviral phylogenetic inference and taxonomy." Retrovirology 2: 50
Ref. 4	Han, G. "Worobey M.(2012a)'An Endogenous Foamy-Like Viral Element in the Coelacanth Genome'." PLoS Pathogens 8: e1002790.
Ref. 5	Han, G.-Z. (2015). "Extensive retroviral diversity in shark." Retrovirology 12(1): 34.

Supplementary Table S7. Dating of Small spotted catshark and Jawless fish ERVs insertion based on LTR-LTR divergence.

Contig number	Integration time (MYA)	Divergence	Host genus	Nucleotide Replacement Rate/Year
CACTIT020000509:15172..24555	35.92	0.0359	*Scyliorhinus canicula*	5.00E-10
CACTIS020002783:218952..226166	15.25	0.0153	*Scyliorhinus canicula*	5.00E-10
CACTIS020009847:53637..62739	15.00	0.0150	*Scyliorhinus canicula*	5.00E-10
CACTIS020015143:83414..93643	14.00	0.0140	*Scyliorhinus canicula*	5.00E-10
CACTIS020023086:60523..69648	13.00	0.0130	*Scyliorhinus canicula*	5.00E-10
CACTIT020000196:10597..20035	10.34	0.0103	*Scyliorhinus canicula*	5.00E-10
CACTIS020021572:66596..75899	10.00	0.0100	*Scyliorhinus canicula*	5.00E-10
CACTIS020013165:19060..28338	9.00	0.0090	*Scyliorhinus canicula*	5.00E-10
CACTIS020019354:71904..81018	6.00	0.0060	*Scyliorhinus canicula*	5.00E-10
CACTIS020005886:179451..188846	5.00	0.0050	*Scyliorhinus canicula*	5.00E-10
CACTIS020018739:23626..32931	4.86	0.0049	*Scyliorhinus canicula*	5.00E-10
CACTIS020019402:131793..140958	4.00	0.0040	*Scyliorhinus canicula*	5.00E-10
CACTIS020017934:52677..61838	3.00	0.0030	*Scyliorhinus canicula*	5.00E-10
CACTIS020014980:317002..326843	2.00	0.0020	*Scyliorhinus canicula*	5.00E-10
CACTIS020011821:50856..60184	1.00	0.0010	*Scyliorhinus canicula*	5.00E-10
FYBX02010699:388666..395609	24.33	0.0730	*Eptatretus burgeri*	1.5E-09
FYBX02009749:146189..155755	15.00	0.0450	*Eptatretus burgeri*	1.5E-09
APJL01068812:4713..12960	13.67	0.0410	*Lethenteron camtschaticum*	1.5E-09
WFAB01001333:6679..21501	10.67	0.0320	*Lethenteron camtschaticum*	1.5E-09
APJL01051162:3542..12101	6.33	0.0190	*Lethenteron camtschaticum*	1.5E-09
WFAB01000452:844674..861915	6.00	0.0180	*Lethenteron camtschaticum*	1.5E-09
WFAB01003073:10447..18542	5.00	0.0150	*Lethenteron camtschaticum*	1.5E-09
JADLOY010000978:220980..227789	2.00	0.0060	*Lethenteron reissneri*	1.5E-09
JADLOY010001726:7510..23435	0.67	0.0020	*Lethenteron reissneri*	1.5E-09
JADLOY010000227:19051..36621	0.33	0.0010	*Lethenteron reissneri*	1.5E-09
JADLOY010000623:174106..191462	0.10	0.0003	*Lethenteron reissneri*	1.5E-09
JAAIYE010000870:135019..151593	2.67	0.0080	*Petromyzon marinus*	1.5E-09
